# A New Approach for Detecting Fundus Lesions Using Image Processing and Deep Neural Network Architecture Based on YOLO Model

**DOI:** 10.3390/s22176441

**Published:** 2022-08-26

**Authors:** Carlos Santos, Marilton Aguiar, Daniel Welfer, Bruno Belloni

**Affiliations:** 1 Computer Center, Federal Institute of Education, Science and Technology Farroupilha, Alegrete 97555-000, Brazil; 2 Postgraduate Program in Computing (PPGC), Federal University of Pelotas, Pelotas 96010-610, Brazil; 3 Postgraduate Program in Computer Science (PPGCC), Departament of Applied Computing (DCOM), Federal University of Santa Maria, Santa Maria 97105-900, Brazil; 4 Federal Institute of Education, Science and Technology Sul-Rio-Grandense, Passo Fundo 99064-440, Brazil

**Keywords:** Diabetic Retinopathy, fundus images, lesions detection, deep learning, YOLO

## Abstract

Diabetic Retinopathy is one of the main causes of vision loss, and in its initial stages, it presents with fundus lesions, such as microaneurysms, hard exudates, hemorrhages, and soft exudates. Computational models capable of detecting these lesions can help in the early diagnosis of the disease and prevent the manifestation of more severe forms of lesions, helping in screening and defining the best form of treatment. However, the detection of these lesions through computerized systems is a challenge due to numerous factors, such as the characteristics of size and shape of the lesions, noise and the contrast of images available in the public datasets of Diabetic Retinopathy, the number of labeled examples of these lesions available in the datasets and the difficulty of deep learning algorithms in detecting very small objects in digital images. Thus, to overcome these problems, this work proposes a new approach based on image processing techniques, data augmentation, transfer learning, and deep neural networks to assist in the medical diagnosis of fundus lesions. The proposed approach was trained, adjusted, and tested using the public DDR and IDRiD Diabetic Retinopathy datasets and implemented in the PyTorch framework based on the YOLOv5 model. The proposed approach reached in the DDR dataset an mAP of 0.2630 for the IoU limit of 0.5 and F1-score of 0.3485 in the validation stage, and an mAP of 0.1540 for the IoU limit of 0.5 and F1-score of 0.2521, in the test stage. The results obtained in the experiments demonstrate that the proposed approach presented superior results to works with the same purpose found in the literature.

## 1. Introduction

Vision is one of the most essential and complex senses; through it, it is possible to see and interact with the world around us. Vision is based on the absorption of light by the photoreceptor cells of the eye [1]. Unfortunately, various diseases can harm the eyes. Thus, taking care of this organ is essential to prevent or even reduce the severity of these diseases. Furthermore, eye health is associated with a better quality of life, so it is necessary to maintain a healthy vision. The human retina is the most complex of eye tissues, having a highly organized structure. The retina receives the visual image produced by the eye’s optical system and converts the light energy into an electrical signal, which, after initial processing, is transmitted through the optic nerve to the visual cortex [2]. It is a thin, semitransparent, multi-layered layer of nervous tissue that lines the inside of the posterior two-thirds of the eyeball wall. It is sensitive to light and can be compared to a film in a photographic camera, being like a screen to project the images seen, which retains the images, translating to the brain through the electrical impulses sent by the optic nerve to the brain [3].

In patients with diabetes, the retina can be affected by the pathology known as Diabetic Retinopathy (DR) [2,4], which occurs when abnormal material is deposited on the blood vessel walls of the retina, which is the known region as the fundus of the eye, causing narrowing and sometimes blockage of the blood vessel, in addition to weakening of the vessel wall and the appearance of fundus lesions [3]. In the United States, between 40 and 45% for people with diabetes have retinopathy. Type 1 diabetics develop a severe form of retinopathy within 20 years in about 60 to 75% cases, even with reasonable diabetes control. In patients with diabetes type 2, generally older, the most frequent retinopathy is non-proliferative [2]. The early stages of DR can be clinically asymptomatic, and if the disease is detected in advanced stages, the treatment can become difficult [5].

Depending on the presence of clinical features, researchers classify DR as Mild Non-Proliferative Diabetic Retinopathy (NPDR); moderate NPDR; severe NPDR; Proliferative Diabetic Retinopathy (PDR); and Diabetic Macular Edema (DME) [6,7,8,9]. DR is usually identified through ophthalmologic examinations that aim to identify retinal lesions, including hard exudate (EX), soft exudate (SE), microaneurysms (MA), and hemorrhages (HE). Microaneurysms are small lesions in the form of small circular red dots that appear on the retina, in saccular shape, caused by a leak from vascular weakness at a certain point and represent the first signs of DR [10,11]. According to the International Council of Ophthalmology [12], microaneurysms are red, isolated spherical dots of varying sizes, which may reflect an abortive attempt to form a new vessel or maybe a weakness of the capillary vessel wall due to loss of normal structural integrity. On the other hand, hemorrhages can occur in the pre-retinal layer, retina, or vitreous and may have a different appearance depending on where they occur [13]. Hard exudates are lipoproteins and other proteins that leak through abnormal retinal vessels [6], being irregularly shaped yellow lesions [10], and may also have a whitish-yellow color, with a shiny appearance and well-defined margins [7], appearing in different dispositions: isolated, in clusters, in confluent trails or a partial or complete ring [13]. Soft exudates are retinal microinfarcts resulting from diabetes, hypertension, retinal vein occlusion, papilledema, collagen diseases, anemia, and leukemia [13], having the form of spots and having a pale appearance, usually in greyish-white tones, and with blurred and irregular edges.

Detection of retinal lesions associated with DR in the early stages is essential for preventing more severe forms of this disease that can cause irreversible vision loss. However, screening patients in the early stages of DR remains challenging as this eye disease is asymptomatic and progresses at a very high rate [14]. Another challenge is the availability of adequate numbers of specialized professionals and infrastructure, especially in developing regions [15], which cannot meet the growing demand due to the increase in diabetes cases in the last two decades [16]. While conventional approaches to identifying DR are effective, their resource demands are high. The level of experience of the health professional who will care for patients and the equipment needed to perform the tests are often insufficient in regions where the rate of diabetes in the local population is high; thus, the detection of DR is more necessary.

Due to these limitations, the severity of DR, and also the impact of this disease on health and quality of life over the people, it is opportune to propose the creation or optimization of procedures, approaches, and computational tools capable of providing fast and accurate support for medical diagnosis, with minimal human intervention. In the literature, approaches based on deep learning have been presented to classify, segment, and detect retinal lesions associated with DR. These works aim to assist in diagnosing DR through deep neural networks, such as [17,18,19,20,21]. However, although these works have achieved promising results, detecting retinal lesions has presented limited results. The limitations are due to the complexities of retinal fundus images (low contrast, irregular lighting, blurred lesion boundaries, shape, and size) and the few examples of the lesions, which cause problems in feature extraction and training approaches based on deep neural networks.

In this context, this work aims to present a state-of-the-art approach based on a pre-trained deep neural network model that, through image pre-processing and data augmentation techniques, can detect fundus lesions and assist in the medical diagnosis and treatment of Diabetic Retinopathy.

The contributions of this work concerning the state-of-the-art can be summarized as follows: (i) presentation of a convolutional neural network structure based on YOLO version 4 and 5 architectures to improve fundus lesions detection and performing real-time inferences on low-cost GPUs; (ii) implementation of a pre-processing block to reduce *outliers* and improve enhancement, to provide a more efficient extraction of features from retinal lesions; and, (iii) method for partial *cropping* of the black background of the fundus images in order to minimize the generation of false positives pixels and the creation of blocks (*tiles*) to increasing the receptive field of the input images and minimizing the loss of information caused by the reduction of the images used at the entrance of the network, especially in the case of small lesions, such as in the case of microaneurysms.

The article is structured as follows. Section 2 describes related works. Section 3 presents the materials and methods used for this work. Section 4 will describe the results obtained through the proposed approach. Section 5 presents the discussions about the results obtained in this work. Finally, in Section 6 we will describe the final considerations.

## 2. Related Work

Li et al. [17] presented a new Diabetic Retinopathy dataset titled *Dataset for Diabetic Retinopathy* (DDR) and evaluated state-of-the-art deep learning models for classification, segmentation, and detection of lesions associated with Diabetic Retinopathy. To evaluate these methods in clinical situations, 13,673 fundus images were collected from 9598 patients. These images were divided into six classes and evaluated by seven specialists to identify the stage of Diabetic Retinopathy. In addition, the authors selected 757 images with Diabetic Retinopathy to annotate the fundus lesions: Microaneurysms, Hemorrhages, Hard Exudates, and Soft Exudates.

The authors used the DDR dataset to evaluate ten deep learning models, including five classification models, two segmentation models, and three detection models. Experimental results demonstrate that research must improve the model performance for microlesion recognition to apply deep learning models to clinical practice. To perform the classification of DR, the authors used the models VGG-16 [22], ResNet-18 [23], GoogLeNet [24], DenseNet-121 [25] and SE-BN-Inception [26]. To perform the segmentation of DR lesions, the authors used the HED [27] and DeepLab-v3+ [28] models, while they used the SSD [29] and YOLO [30] models for the detection single-stage. The authors evaluated the models with the metrics *mean Average Precision* and the mean Intersection over Union (mIoU) [31], obtained in the set of validation and testing of the DDR dataset. Although the authors obtained an accuracy of 0.8284 in Accuracy with the SE-BN-Inception model for the classification of Diabetic Retinopathy, the detection and segmentation models performed poorly in detecting fundus lesions.

The work by Porwal et al. [18] presents results of deep learning models used for segmentation, classification, and detection of fundus lesions during the *IDRiD: Diabetic Retinopathy—Segmentation and Grading Challenge*. The main contribution was the availability of the set of public images of Diabetic Retinopathy called IDRiD. Most of the teams participating in the challenge explored the U-Net architecture for segmenting (microaneurysms, hemorrhages, hard exudates, and soft exudates) [32]. The U-Net architecture is an extended version of the fully convolutional networks (FCN), providing more precise segmentation, even in small training sets [33]. In the classification challenge, they categorized the fundus images according to the severity level of DR and Diabetic Macular Edema. The team that achieved the best results presented a method based on a ResNet [23] architecture and Deep Layer Aggregation (DLA) [34]. Finally, the detection challenge aimed to obtain the location of the Optical Disc and the Fovea. The winning team presented a method based on the Mask R-CNN model to locate and segment the Optical Disc and Fovea simultaneously.

First, the authors pre-processed the fundus images to a fixed size to use them as neural network input. Then, they performed a scan on the image to generate the region proposals. Then, the authors classified proposals into different classes and created a binary mask for each object. Next, they used a ResNet-50 architecture to extract features from the images and a Feature Pyramid Network (FPN) to generate feature maps at different scales and extract regions of interest from the images. A Region Proposal Network (RPN) then traverses the feature maps and locates regions that contain objects. Finally, architectural branches are employed to obtain the label, mask, and bounding box for each region of interest. Transfer learning technique was applied to train the model. The authors started with a learning rate of 0.001 and a *momentum* of 0.9. They trained the network with 20 epochs. In the Optical Disc and Fovea segmentation and detection task, the team that obtained the best result in the test dataset achieved a Jaccard coefficient equal to 0.9338.

Mateen et al. [19] proposed a pre-trained framework based on a convolutional neural network to detect exudates in fundus images through transfer learning. In the proposed structure, the authors combined three pre-trained network architectures to perform the fusion of features since, according to the authors, different architectures could capture various features. The three models used to compose the framework proposed by the authors are Inception-v3 [35], VGG-19 [22] and ResNet-50 [23]. In addition, the collected features are treated as input in the fully connected layers to perform further actions, such as classification, performed through the *Softmax* function. When using the e-Ophtha dataset, the highest accuracy obtained individually by the Inception-v3, ResNet-50, and VGG-19 architectures was 93.67%, 97.80%, and 95.80%, respectively, while the approach proposed by the authors reached a classification accuracy of 98.43%. Moreover, using the DIARETDB1 dataset, the highest accuracy obtained individually by the Inception-v3, ResNet-50, and VGG-19 architectures was 93.57%, 97.90% and 95.50%, respectively, while the approach proposed by the authors reached a classification accuracy of 98.91%

The work by Alyoubi et al. [20] presented a DR diagnostic system that classifies fundus images into five stages (no DR, mild DR, moderate DR, severe DR, and proliferative DR) and the location of lesions on the retinal surface. The system is composed of two models based on deep learning. The first model used is a CNN512, in which the images are used to classify in one of the five stages of DR. The public datasets DDR and Kaggle APTOS 2019 [36] were used. The second model was a YOLOv3 [37], adopted to detect DR lesions. Finally, both proposed frameworks, CNN512 and YOLOv3, were combined to classify DR images and locate DR lesions. The data balancing of the data sets used when performing the training of the models was not performed. In classifying the type of DR, the CNN512 model achieved an accuracy of 88.6% and 84.1% in the DDR and APTOS Kaggle 2019 public datasets, respectively, while the YOLOv3 model, adopted to detect the DR lesions, obtained a mAP of 0.216 in the detection of lesions in the DDR dataset.

The main limitation of the work was not performing the balancing in the retinal image datasets to perform the training of the models, which may have generated a bias in the detection of lesions. For example, there are lesions with a significantly higher number of examples concerning the others, as in the case of hard exudates. This imbalance in the training of models can make the deep neural network tend to classify objects in the majority classes, causing the model to have its generalization ability impaired. In future work, the authors claim that it is necessary to balance the number of examples in the data sets to train the models and conduct experiments with the YOLOv4 and YOLOv5 models to verify the performance of these models in the detection of fundus lesions.

The work by Dai et al. [21] presented a deep learning system, called DeepDR, to detect early and late stages of Diabetic Retinopathy using 466,247 fundus images of 121,342 patients with diabetes. The DeepDR system architecture had three subnetworks: an image quality assessment subnetwork, the subnet for lesion recognition, and the subnet for DR classification. These subnets were developed based on a ResNet [23] architecture and a Mask R-CNN [38] architecture, responsible for performing the detection of lesions in 2 stages: a preliminary stage, in which regions of interest (RoI) are selected and then, in a second stage, check for the presence of lesions in the regions verified in the previous step [39,40,41].

First, the authors used 466,247 images to train the image quality assessment subnet to check for quality problems in terms of artifacts in the retinal images. After this analysis, 415,139 images were used to train the DR classification subnet to classify the images: no DR, mild nonproliferative DR, moderate nonproliferative DR, severe nonproliferative DR, or proliferative DR. The subnetwork for detecting and segmenting the lesions was trained using 10,280 images with annotations of retinal lesions: microaneurysms, soft exudates, hard exudates, and hemorrhages. For microaneurysms, the value obtained from IoU (*Intersection Over Union*) [42,43] was not presented. For soft exudates, hard exudates, and hemorrhages, IoU of 0.941, 0.954, and 0.967 were obtained.

The study had limitations. The first one was using only a private DR dataset to train the deep learning models, making it difficult to reproduce the results obtained by the authors using the same method. In the validation step, the authors used the Kaggle eyePACS [44] public DR dataset only. The second limitation is that the subnet that detects the lesions was tested only on the local validation dataset due to the lack of lesion annotations in the public dataset that the authors used. Therefore, in future work, the authors claim that further validation through public datasets is necessary to assess the proposed deep learning system’s performance in classifying DR and detecting fundus lesions.

The works in the literature applied deep neural networks to identify DR, but the deep learning models presented limitations. The work by Porwal et al. [18] showed results in detecting only the fovea and the optical disc, while the work by Mateen et al. [19] only exudates. The work by Li et al. [17] showed promising results in the classification of DR but limited results in the detection of fundus lesions. The work by Alyoubi et al. [20] showed promising results in detecting lesions but did not balance the datasets used, which possibly impacted the results presented. The work proposed by Dai et al. [21] used a private dataset for training and validating the model responsible for detecting lesions, which makes a fair comparison and validation with the models we propose impossible. Furthermore, the model used by Dai et al. [21] is based on a model that performs the detection of objects in 2 stages, unlike the conception of our work, which performs the *Single-Stage* detection after pre-processing and augmentation of image data. A summarized comparison between related works is presented in Table 1.

## 3. Materials and Methods

**Figure 1:**
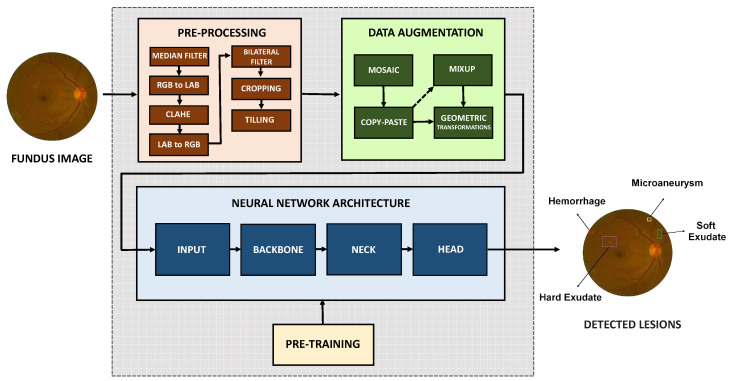
Block diagram of the proposed approach. First, the images are passed to the Pre-processing block for noise filtering, contrast improvement, partial elimination of the black background of the images, and creation of *tiles*. Then, the pre-processed images are transferred to the Data Augmentation block, where sub-images are artificially created that will be used in the neural network input layer for training the proposed approach, which will be carried out after a pre-stage step training the network with the weights fitted to the Common Objects in Context (COCO) dataset.

The *pipeline* of the proposed approach is presented in the form of a block diagram in Figure 1. The proposed approach was based on the YOLOv5 [45,46,47,48,49,50,51] deep neural network model and implemented through the open-source machine learning library PyTorch (https://pytorch.org, accessed on 22 August 2022). The model was trained with 8000 epochs and 32 batches per epoch, with a learning rate of 0.01 and a *momentum* rate of 0.937. The size of the bounding box anchors was adaptively calculated [52], through a genetic algorithm, which optimizes the anchors after a scan performed by the unsupervised K-means algorithm [53] before the step of training.

An anchor set more adjusted improves detection accuracy and speed [54]. Anchors are the initial sizes (width, height) of bounding boxes that will be adjusted to the size closest to the object to be detected using some neural network output (final feature maps). In this way, the network will not predict the final size of the object but only adjust the size of the anchor closest to the object’s size. For this reason, YOLO is regarded as a method that treats object detection as a regression problem, in which a single neural network predicts the bounding boxes and class occurrence probabilities directly on the complete image being evaluated. Moreover, as all detection is performed in one network (*Single-Stage*), the neural network model can be directly optimized end-to-end [30].

In the proposed approach, detection is performed in the final layers and at three scales, as proposed in the YOLOv3 [37] model, allowing learning objects in different sizes, the scales being: 19×19, specialized in object detection of large size; 38×38, which specializes in detecting medium-sized objects; and, 76×76, which specializes in detecting small objects. Each of these outputs or detection “heads” has a separate set of anchor scales. In YOLOv3, 9 anchor sizes are used, with 3 anchors per detection head. After detection, the confidence percentage was reached for each identified lesion. To carry out the experiments a Core i7-7700K 8 × 4.6 GHz device, 32 GB of RAM and an NVIDIA Titan Xp GPU with 12 GB of VRAM were used.

YOLOv5 is a single-stage detection model capable of detecting objects without a preliminary step, as in the case of two-stage detectors, which use a preliminary stage where regions of importance are then classified to check if objects have been detected in those areas. The advantage of a single-stage detector is the speed at which it can make real-time inferences. Furthermore, another feature of this model type is the possibility of working on edge devices and with low-cost hardware, whose training can be performed with just one GPU [55]. Therefore, we intend to present an approach that achieves greater precision than approaches with the same purpose presented in the literature. Next, we methodologically detail each step that makes up the pipeline of the proposed approach.

### 3.1. Dataset

**Figure 2:**
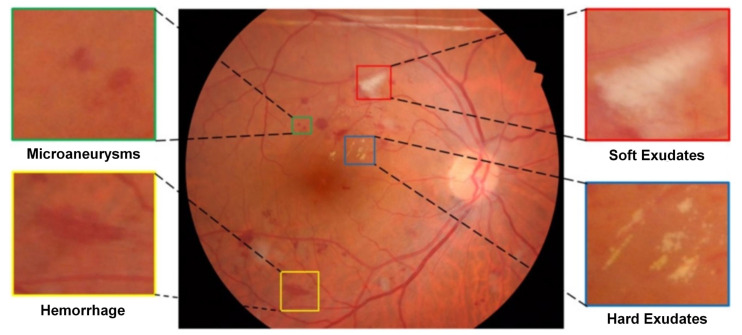
Representation of fundus image with the lesions annotated: Microaneurysms, Hemorrhages, Soft Exudates, and Hard Exudates.

The DDR image dataset was used in this work, which has 757 images labeled in JPEG format with variable sizes. Second [17] to capture these images 45 TRC NW48, Nikon D5200, and Canon CR 2 cameras were used. In addition, the lesions contained in these fundus images contain annotations (*Ground Truth*), as illustrated in Figure 2.

Table 2 presents attributes of the DDR dataset, such as the number of images, the resolution of the images, the type of annotations, the number of images with annotations for MA, HE, EX, and SE, and the total amount of annotations by lesion type before the data augmentation step.

Data were collected using single-view images. The bounding box annotations were generated automatically from the *pixel* level annotations of the lesions [17]. Although the DDR dataset is of good quality, training the deep neural network of the proposed approach has challenges, such as the small number of annotated fundus lesions and the variability in size and shape of these lesions. Another factor that generates problems in training deep learning models to detect retinal lesions is the reduced size of some types of lesions, as in the case of microaneurysms. Data augmentation was performed to circumvent problems related to the sub-sampling of the data set due to the small number of examples by creating artificial images derived from the original images and annotations of the fundus lesions. This data augmentation and the other techniques applied to overcome the challenges mentioned above will be discussed in the following sections.

### 3.2. Pre-Processing and Image Preparation

The use of a pre-processing block aims to (i) improve the quality of images through the elimination of periodic or random patterns through the application of filters and (ii) increase the image enhancement to improve and accentuate the characteristics of the lesions that will be used for training the deep neural network. The treatment of the images aims at (i) filtering noise generated during the capture of fundus images, (ii) correcting lighting deformities, and (iii) improving the contrast and enhancement of the images [56].

For image smoothing, the median filter of size 5×5 was used. The median filter is non-linear and very effective in removing impulsive noise (irregular pulses of large amplitudes), such as *Salt & Pepper* [57,58] noise. Moreover, the contrast-limited histogram adaptive equalization technique (CLAHE) [20,59] was used to enhance the images. This technique was initially developed for low contrast image enhancement and is an evolution of the histogram equalization method [60], and has been used as part of pre-processing *pipelines* to improve image quality. medical images [61]. However, before applying the CLAHE algorithm to the fundus images of the dataset, it was necessary to define the most suitable color space to perform the image enhancement (i.e., RGB, HSV, or LAB).

The background suppression was performed as the last step in the pre-processing and image preparation stage. As the works by El abbadi and Hammod [62] and Alyoubi et al. [20], a pre-processing step for partial *cropping* of the black background of the retinal images was performed, as illustrated in the Supplementary Materials. According to El abbadi and Hammod [62], the importance of removing the black background from the retinal image is related to the generation of false positives during the detection of lesions, especially at the retina border, where there is a similarity of the retinal border with the blood vessels. Furthermore, in the case of fundus images, only the *pixels* of the retina have significant information; the rest is considered the background. Therefore, it is essential to locate the area of interest and remove unwanted features related to the image’s background.

Details about the median filter, CLAHE, and the suppression of the useless retinal background are presented in the Supplementary Materials attached to this article. It is important to note that this additional document presents images where these pre-processing methods are tested and where the used measures are clearly presented and discussed.

Pre-processing techniques were explored to improve the performance of the proposed approach, especially in detecting microlesions for better smoothing and enhancement of the fundus images. In addition, the black background that caused the generation of false positives was partially removed, and the Tilling of the original images was applied to use the resulting image blocks in the training of the deep neural network. Finally, a Data Augmentation step was applied after pre-processing the fundus images, as we will explain in the next section.

### 3.3. Data Augmentation

In the proposed approach, a data augmentation was performed from the images and labeled lesions available in the DDR dataset. Then, for each training batch, the model was configured to pass the images through a data loader that creates the artificial images while they are accessed. The data loader did the following types of augmentations: *Mosaic*, *MixUp*, *Copy-Paste* and Geometric Transformations.

This technique works in real-time, i.e., *on-the-fly* [63,64], and new examples are not generated in advance (before training). Thus, at each training performed, a random number of artificially created examples are generated and passed on for training the neural network. Each data augmentation technique is applied to all images in the batch, except the Mix-Up, which was set to randomly apply to 50% of the images in the batch. Details on the methods applied in the data augmentation block of the fundus images are presented in the Supplementary Materials attached to this article. It is important to note that this additional document discusses the methods used and the images with the results obtained in this step.

After performing the data increase, we also had to deal with the problem related to data imbalance. Models based on deep learning generally try to minimize the number of errors when classifying new data. Therefore, the cost of different errors must be equal. However, the costs of different errors are often unequal in real-world applications. For example, in the area of medical diagnosis, the cost of misdiagnosing a sick patient as healthy (False Negative) can be much higher than accidentally diagnosing a healthy person as sick (False Positive) since the former type of error can result in the loss of a life [65].

There are cases in which data imbalance causes biases in the training of models, including generating uncertainties about the results obtained [66,67]. In the case of eye fundus lesions, an imbalance in the number of examples of different fundus lesions was verified, as shown in Table 2. This imbalance can become even more significant after the application of the data increase step since the number of new examples created after this step is random, and it is impossible to accurately predict the number of new examples generated for each lesion.

To balance the number of examples of each lesion, during training the method *Threshold-Moving* [68,69,70] was used through the parameter image-weights, in which the samples of training set images are weighted by their *mean Average Precision* (mAP) inverse of the previous epoch test. Unlike uniformly sampling the images during training, as in conventional training, sampling during training is based on the weighted images based on the result obtained by a certain evaluation metric calculated in the test of the previous epoch. of training. This method moves the decision boundary so that minority class examples can easily predict correctly [71,72].

The *Threshold-Moving* method was used to minimize the imbalance of the dataset and reduce the possibility of biases during the classification due to the presence of classes with a significantly larger number of examples. Due to the need for many examples to obtain more accurate results, we chose not to use the subsampling technique of the majority classes. Likewise, the oversampling of minority classes was also discarded because the number of examples of these classes to equate them to the majority classes would not reflect the natural incidence of fundus lesions.

Our approach used a method to minimize the imbalance problem in the number of examples of lesions to avoid possible overfitting of the model associated with misclassification of lesions in the majority class. After the step of increasing and balancing the data, our method was trained to perform the detection of fundus lesions. The next section will discuss the architecture of the deep neural network used in our proposed approach.

### 3.4. Deep Neural Network Architecture

After the pre-processing and data augmentation steps described above, the image set of the DDR dataset was split into a training set (50%), validation set (20%), and test set (30%), the same proportion performed in the work by Li et al. [17]. The images of one set are not present in the others to avoid bias during the evaluation of the proposed approach. A validation step to fine-tune the hyperparameters of the architecture and a test step to assess the generalization capability of the neural network was used. In addition, the public Diabetic Retinopathy dataset IDRiD [18] was also used to validate our proposed approach.

In our approach, we used the architecture of the YOLOv5 [20,45,46,47,48,49,50,53,73] model as a basis. This model currently has four versions: *s* (*small*), *m* (*medium*), *l* (*large*) and *x* (*extra-large*). Each version has different features. Before choosing the YOLOv5 *s* as the base architecture for our proposed approach, different versions of YOLOv5 have been tried. The quantities of depth (*depth*) and scale (*width*) multipliers of convolutional cores of the adopted model are 0.33 and 0.50, respectively. According to Iyer et al. [74], YOLOv5s achieves precision equivalent to YOLOv3 (https://pjreddie.com/darknet/yolo/, accessed on 22 August 2022), but with superior performance in performing real-time inferences at a lower computational cost.

The YOLOv5 *s* was used because it achieved the best results in detecting fundus lesions and at a lower computational cost compared to other versions. The explanation for the shorter version of YOLOv5 to have obtained the best results lies in the fact that the fundus microlesions present a gradient dissipation problem when training the versions of YOLOv5 with greater depth and number of parameters. Thus, using a model with a smaller number of parameters allowed us to use fewer hardware resources, thus enabling the detection of fundus lesions through low-cost GPUs without impacting the precision of the proposed approach. The deep neural network structure used in our approach has a total of 283 layers, 7.2 million parameters, and 17.1 GFLOPs. According to Yu et al. [75], GFLOPs represent the 1 billion worth of FLOPs (floating-point math operations). Another advantage of using the YOLOv5 model as the basis of this approach is the possibility of integration and portability with different types of projects, including mobile devices. This feature is associated with the fact that this model has been implemented natively in PyTorch.

The network Backbone was used as a pre-trained feature extractor on an image classification dataset, useful for detecting objects in the last layers of the network. The *Backbone* used in the experiments is composed of a CSP-Darknet-53. The convolutional neural network Darknet-53 was initially used as *Backbone* of the YOLOv3 [37] model, replacing its predecessor Darknet-19 [30], as it includes the use of residual connections as well as more layers since it has 53 depth layers [37]. Furthermore, its architecture is built by consecutive layers of convolution 3×3 and 1×1 followed by a jump connection, which helps activations propagate through deeper layers without gradient dissipation.

The CSP proposed by Wang et al. [76] can be applied in different architectures, such as ResNet [23], ResNeXt [77], DenseNet [25], YOLOv4 [55] and YOLOv5 [45,46] as it not only produces a reduction in computational cost and memory usage of these networks but also brings benefits such as improved inference speed and increased precision. These goals are achieved by partitioning the base layer feature map into two parts. Then, the parts are merged through a hierarchy of crossed stages, whose main idea is to make the gradient propagate through different network paths.

**Figure 3:**
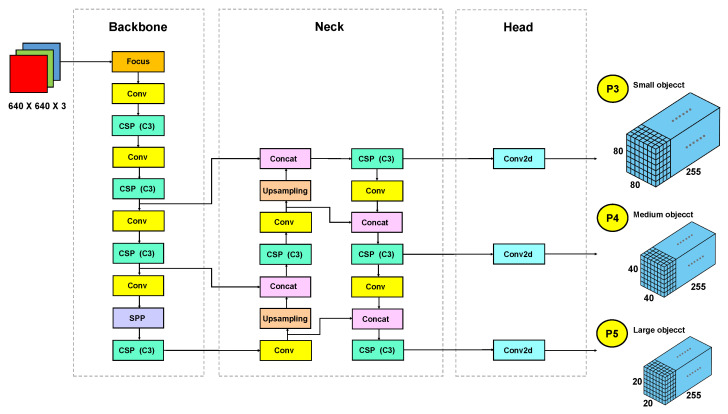
The neural network architecture block diagram composes the proposed approach for detecting fundus lesions. The structure is divided into three main blocks: *Backbone*, *Neck* and *Head*. The *Backbone* block consists of a Focus module, four Conv modules, four CSP modules (C3), and an SPP module. The *Neck* block consists of four Conv modules and four CSP modules (C3). The network input receives images of size 640×640×3, and the output is composed of three detection heads: the P3 layer, responsible for detecting small objects; layer P4, responsible for detecting medium objects; and, finally, the P5 layer, responsible for detecting large objects. CSP (C3), Cross Stage Partial Network C3; SPP, Spatial Pyramid Pooling; Conv, Convolution module; Concat, concatenation; Conv2d, 2D convolution layer.

The block diagram of the neural network architecture that composes the proposed approach, responsible for detecting fundus lesions, is illustrated in Figure 3. As shown in, the network architecture is based on the structure of YOLOv5 version small [45,46,74] and is divided into three main blocks: *Backbone*, *Neck* and *Head*. The network input layer size is 640×640×3, where the first two values correspond to the height and width in *pixels*, and the third value corresponds to the number of channels in the input image. The DDR dataset makes the bounding box annotations of fundus lesions available for deep neural network training. These bounding boxes were used to calculate the initial anchor sizes. In the architectures of the YOLO family, we usually configure the sizes of the initial anchors before training the model.

In training the proposed approach, we produced bounding boxes with predictions based on the lengths of the initial anchors. Then a comparison of the bounding box of the detected object is performed with the bounding box of the object annotated (*Ground Truth*). Then we used the result of this comparison to update the neural network weights during the training stage. Therefore, defining the initial size of the anchors is essential, especially when training the neural network on objects with sizes different from the standard dimensions of anchors, which are typically calculated based on objects from datasets such as COCO, for example. The YOLOv5 model repository has a function that performs the adaptive calculation of the anchors, in which, when training the neural network, it is possible to enable the “auto-anchor” option so that the best values for the docking boxes automatically. This function was used before training the proposed approach to ensure that the anchors were adjusted according to the sizes of fundus lesions present in the dataset used in the experiment.

The *Backbone* structure of the neural network starts with the *Focus* module, responsible for performing a slicing operation. In the case of the neural network structure of the proposed approach, when we insert an image of size 640×640×3 in the module *Focus*, a slicing operation is performed on this image to generate a map of size features 304×304×64. Still in *Backbone*, the modules *Conv* are composed of a 2d convolution, followed by a batch normalization. The batch normalization reduces the number of training cycles needed to train deep networks, providing a regularization and reducing generalization error [78]. After batch normalization, the activation function *Sigmoid Linear Unit* (SiLU) [79], derived from the function *Rectified Linear* (ReLU) [80] is applied.

In network architecture, the CSP module (C3) is used both in the *Backbone* and in the *Neck* of the network. These CSPs were used to connect the front and back layers of the network, aiming to improve the model inference speed without compromising its precision. Also, they allow better integration of different parts that make up the neural network, in addition to a reduction in the size of the model [45]. These C3 modules have in their structure three Conv modules and a *Bottleneck* [23] module. The module *Bottleneck* consists of two Conv modules followed by an addition operation (*add*), responsible for adding tensors without expanding the image dimension.

The *Backbone* of the proposed approach is composed of four CSP modules (C3). After *Bottleneck* module, each C3 module, there is a concatenation module (*Concat*) so that the features that were divided at the beginning of the C3 block are regrouped, expanding the dimensions of the tensors. The flow and constitution of the various modules that make up the *Backbone* were illustrated in Figure 3. Another *Backbone* component of the proposed approach is the SPP module (*Spatial Pyramid Pooling*) [81]. With SPP, it is possible to introduce multiple variable-scale *pools* concatenated to form a 1 dimension vector for the FC layer. As in He et al. [81], the MaxPool method was used with groupings of size equal to 1×1, 5×5, 9×9 and 13×13, followed by the *Concat* operation to concatenate feature maps at different scales.

**Figure 4:**
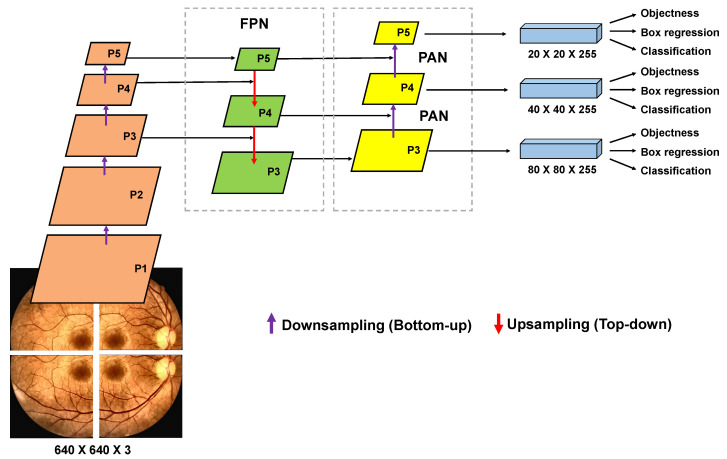
FPN+PAN structure used in the *Neck* of the neural network architecture of the proposed approach. FPN has a *Top-Down* structure and lateral connections that enable it to build feature maps with high-level semantic meaning, which are used to detect objects at different scales. The PAN architecture conveys strong localization features from the lower-feature maps to the upper-feature maps (*Bottom-up*). The two structures combined to reinforce the ability to merge characteristics of the *Neck* structure. The detection of lesions is performed in layers P3, P4 and P5 of the FPN+PAN structure, having outputs with sizes of 80×80×255, 40×40×255 and 20×20×255, respectively. FPN, Feature Pyramid Network; PAN, Path Aggregation Network.

The *Backbone* structure is responsible for extracting feature maps of different sizes from the input image through multiple convolutions and clusters [82]. The *Neck* structure, in turn, is responsible for merging these feature maps obtained from different levels of the architecture to obtain more contextual information and reduce problems related to information loss during the process of extracting features from the images [45]. In the process of merging the feature maps from the *Backbone*, the *Feature Pyramid Network* (FPN) [83] and the PAN (*Path Aggregation Network*) [84] are used as illustrated in Figure 4. The structure of the FPN itself can be extensive, as the spatial information may need to be propagated to hundreds of layers. In this context, an FPN in conjunction with a PAN was used. The PAN structure follows an additional upward path than the downward path taken by the FPN, helping to shorten this path by using lateral connections as a shortcut.

In the structure of *Neck* four CSP modules (C3) were used, as illustrated in Figure 3. These C3 modules were adopted to strengthen the ability to integrate the characteristics extracted from the lesions during the propagation of this information in the neural network. In Figure 4 it is possible to observe that the detection of lesions is performed in layers P3 (small objects), P4 (medium objects) and P5 (large objects), with sizes of 80×80×255, 40×40×255 and 20×20×255, respectively.

Finally, the *Head* part of the neural network is responsible for making the dense prediction (final prediction). This part is composed of a vector that contains the predicted bounding box (central coordinates, height, width), the confidence score of the prediction, and the label of the class to which the detected object belongs. The prediction mechanism used in the *Head* of the deep neural network architecture of the proposed approach is equivalent to the one used in YOLOv3 [37]. A bounding box predicts each object, and in case several bounding boxes are detected for the same object, then we apply the NMS technique, which allows us to discard bounding boxes with an IoU below a predefined threshold as shown in Table 3. The *Head* structure used in our approach is composed of 3 layers responsible for performing the detection of fundus lesions, each of these layers dividing the image into grid cells of sizes 20×20×255, 40×40×255 and 80×80×255, as illustrated in Figure 3 and Figure 4. The smaller the size of feature maps, the larger the image area to which each grid unit in the feature map corresponds, indicating that it is suitable for detecting large objects from feature maps of size 20×20×255. In contrast, feature maps of size 80×80×255 are better suited for detecting small objects.

The final loss function used in the proposed approach is calculated based on the confidence score (*Objectness*), the classification score (*Class Probability*) and the bounding box regression score (*Bounding Box Regression*), according to Equation (1). *Objectness* determines whether there are objects in the grid cell, *Class Probability* determines which category objects that are in a grid cell belong to, and *Bounding Box Regression* is just calculated when the box predicted contains objects. In this case, the *Bounding Box Regression* calculation is performed by comparing the predicted box with the box associated with the *Ground Truth* of the detected object.

(1) $$Loss=L_{Objectness}+L_{ClassProbability}+L_{BoundingBoxRegression}$$

To calculate the confidence score loss (*objecteness*) and classification score (*class probability*) functions, the *Binary Cross-Entropy* with the PyTorch Logits function [85] was used. To calculate the loss function referring to the bounding box regression, the loss function *Generalized Intersection over Union* (GIoU) [86,87,88] was used.

In the post-processing of the detection of fundus lesions, it was necessary to perform the screening and removal of duplicated bounding boxes representing the same object. The NMS technique was used for that, which keeps the bounding box detected with a higher precision index. Therefore, the NMS method used is based on the obtained IoU values (IoU_nms), in which a threshold of 0.45 [48] was set for the training step.

### 3.5. Pre-Training

Transfer learning is a method employed in the area of machine learning that consists of reusing information learned in a given task as a starting point for the solution of a new task [89]. This method is often used when it is not possible to obtain a large-scale dataset with labeled objects to solve a particular Computer Vision task [90]. In this context, as the public DR datasets do not contain a large number of labeled lesions, in addition to a data augmentation step, the proposed approach has a pre-training step. In this step, the *Transfer Learning* with pre-trained weights on the COCO [91] followed by the *Fine-Tuning* dataset from the last layers of the neural network were applied. To fine-tune the proposed approach, we kept the weights of the first layers and changed only the weights of the last layers of the neural network.

COCO provides a large dataset with labeled images for object detection tasks. The neural network output of the proposed approach was modified to suit the problem of detecting fundus lesions, preserving the knowledge (weights) of the initial layers. The reuse of information from these initial layers is essential to obtain the most basic characteristics of fundus lesions, such as contours, edges, etc. In addition, pre-training enabled a reduction in computational cost and training time of the proposed approach. The method adopted to carry out the transfer of learning was based on the work proposed by Franke et al. [92] and consists of the four steps presented below:

1. The initial layers of the architecture of the proposed approach, focused on detecting the most fundamental characteristics of objects, were pre-trained with the weights of the COCO dataset, composed of 80 categories.
2. The last three layers (out of a total of 283) that make up the *Head* of the architecture of the proposed approach are cut and replaced by new layers.
3. The new layers added are adjusted by training the neural network on the DR dataset, while the weights of the initial layers are frozen.
4. After fine-tuning the *Head* layers of the architecture, the entire neural network is unfrozen and retrained so that minor adjustments to the weights are performed across the entire network.

The fine-tuning of the neural network aimed to optimize the proposed approach to achieve more accurate results. So, hyperparameters, such as batch size, number of epochs, and learning rate, were adjusted. According to Franke et al. [92], the optimization of hyperparameters aims to find a set of values that produces an ideal model in which a predefined loss function is minimized. As in work proposed by Franke et al. [92], the methodology adopted to fine-tune the proposed approach consisted of the following steps:

1. For each adjustment performed, a hyperparameter value is varied, and the proposed approach is retrained, keeping the other hyperparameter values constant.
2. The effect of this change is analyzed through the performance evaluation of the proposed approach with the metrics *Average Precision* (AP) and *mean Average Precision* (mAP), which will be presented and discussed in the next section of this article.
3. If there is an improvement in the metric values, the hyperparameter value is further adjusted (increased or decreased) until the local maximum is reached.
4. The exact process is carried out for the other hyperparameters until an optimal set of values is obtained that produces the maximum results of AP and mAP for the detection of the investigated fundus lesions.

After performing these steps using the validation dataset from the DDR, the ideal fit for hyperparameters was found, as shown in Table 3. With the hyperparameter values adequately adjusted, the next step was to evaluate the proposed approach in the test set of the dataset used in the experiments. In the next section, the metrics used to evaluate the performance of the proposed approach will be presented and discussed.

### 3.6. Performance Metrics

Typically, these models are evaluated by their performance on a dataset’s validation/test set, measured using different metrics. The metrics adopted to evaluate a model must follow the type of task being investigated so that it is possible to adequately and quantitatively compare the performance of this model. For example, quantitative evaluation is performed for object detection tasks in images by estimating overlapping regions between detected images and annotating bounding boxes of objects in original images (*Ground Truth*).

Metrics typically used to evaluate problems involving object detection and segmentation of instances in images are the Intersection over Union (IoU) [31,86], the *Average Precision* (AP ) [38,55,93,94] and the *mean Average Precision* (mAP) [30,31,37,40]. The IoU, also identified by the similarity coefficient of Jaccard [42,43] is a statistic to estimate the similarity between two sets of samples. The Intersection over Union is obtained by the ratio between the Area of Overlap and the Area of Union of the predicted bounding boxes and the Ground Truth bounding boxes.

The *Average Precision* corresponds to the *Area Under the Curve* (AUC) of *Precision* ×*Recall*, also called the PR [95] curve. With the Precision and Recall values, it is possible to plot a graph, where the *y* axis is the *Precision* and the *x* axis is the *Recall*. Recall, and Precision are then calculated for each class by applying the formulas for each image, as shown in Equations (2) and (3), respectively:

(2) $$Recall=\frac{TP}{TP+FN}=\frac{\mathrm{Objects detected correctly}}{\mathrm{All Ground Truth objects}}$$

(3) $$Precision=\frac{TP}{TP+FP}=\frac{\mathrm{Objects detected correctly}}{\mathrm{All objects detected}}$$

*Precision* and *Recall* are useful measures to assess the efficiency of a model in predicting classes when they are unbalanced. The Precision×Recall (PR curve) presents the trade-off between *Precision* and *Recall* for different thresholds. The PR curve is an important tool for analyzing the results of a predictor. The PR curve is an important tool for analyzing the results of a predictor.

The same approach used in the COCO [96] challenge were used to perform the AP calculation, a range of threshold values of IoU, calculate the average of the AP for each IoU, and then obtain a final average of AP. Another critical aspect of calculating the AP in the COCO challenge is that 101 recovery points are used in the PR [96] curve.

Another way to evaluate models that perform object detection is through mAP, a metric widely used to evaluate deep learning models [30,31,37,40]. Its main feature is the ability to compare different models, contrasting precision with recall. The definition of the mAP metric for object detection was first formalized in the PASCAL VOC challenge. To calculate mAP, just average the *Average Precision* calculated for all object classes [95], as shown in Equation (4). Although it is not simple to quantify and interpret the results of a model, mAP is a metric that helps evaluate deep learning models that perform object detection.

(4) $$mAP=\frac{1}{N}\sum_{i=1}^{N}AP_{i}$$

## 4. Experiments and Results

The performance evaluation of the proposed approach experiments was performed using the public DDR Diabetic Retinopathy dataset. To avoid biasing the results, divided the data set into training, validation, and test sets in a proportion of 50%, 20%, and 30%, respectively. The architecture was implemented and trained based on the YOLOv5 model. First, we perform transfer learning based on the pre-trained weights in the COCO dataset, then fine-tune the detection layers of the architecture, and finally, we retrain the entire neural network.

During the training of the proposed approach was used the regularization method *Early Stopping* [97]. With this technique, it was not necessary to statically define the number of epochs necessary for training the proposed approach since the classification precision is calculated in the validation data at the end of each epoch. When the precision stops improving, the training is finished. Therefore, with the use of *Early Stopping*, it was possible to avoid problems such as *underfitting*, in which the neural network cannot extract enough features from the images during training due to an insufficient number of epochs; and, *overfitting*, where the neural network overfits the training data due to an excessive amount of epochs [98]. To do so, a parameter was defined to terminate training if the classification precision has not improved during the last 100 epochs, as shown in Table 3.

In addition to the *Early Stopping* method, the *Dropout* [99] technique was used to help regularize the proposed approach. This technique is widely used to regularize the training of deep neural networks [100]. *Dropout* helps to regularize the model [101], without modifying the cost function. Also, with the use of *Dropout* some hidden neurons of the neural network are randomly and temporarily turned off without changing the input and output neurons. Therefore, this technique causes some neurons not to function according to a certain probability during training.

*Dropout* helps regularization because it reduces complex co-adaptations of neurons, causing some neurons to be forced to learn features that *a priori* would be learned by other neurons in the architecture. In short, the main idea is to drop units (neurons) randomly (along with their connections) from the neural network during training, preventing the units from adapting too much to the data [99], reducing the possibility of problems related to *overfitting* of the neural network after data augmentation, for example. The parameter defined in the proposed approach for using *Dropout* is shown in Table 3.

The proposed approach was evaluated with the AP and mAP metrics to compare the results. These metrics are often used to measure the precision of deep learning algorithms that perform object detection [29,37]. The proposed approach was compared with related approaches found in the literature, including, SSD [17], YOLO [17], YOLOv3 [20], YOLOv4 [59] and YOLOv5 (unmodified) as shown in Table 4. After the experiments carried out during the validation step of the proposed approach in the DDR dataset using the Stochastic Gradient Descent (SGD) optimizer, the best result was obtained using the *Tilling* method, with a mAP of 0.2490 (indicated in bold font) and values of AP with a limit of IoU of 0.5 equal to 0.2290, 0.3280, 0.1050 and 0.3330, for Hard Exudates (EX), Soft Exudates (SE), Microaneurysms (MA) and Hemorrhages (HE), respectively, as shown in Table 4.

To investigate the results of our proposed approach, the PR curve instead of the ROC curve (*Receiver Operating Characteristic*)  [102] was chosen to be analyzed. It is important to observe that the ROC curve is not recommended for situations where the dataset presents an imbalance in the number of examples among the investigated classes. In these cases, the ROC curve usually presents a very high AUC due to the predictor correctly classifying the class with the highest number of examples (majority class) [103,104].

*Precision* and *Recall* were used to evaluate the results obtained, which are performance metrics commonly used to evaluate image classification and information retrieval systems. Generally speaking, Precision and Recall are not discussed in isolation, and some issues may require a higher *Recall* over *Precision*, or vice versa, depending on the importance given to false positives versus false negatives. In classification problems involving medical images, for example, what is generally desired is to minimize the incidence of false negatives; therefore, a high *Recall* becomes more important than a high *Precision* since a false negative can imply a wrong medical diagnosis and, therefore, patient risks.

**Figure 5:**
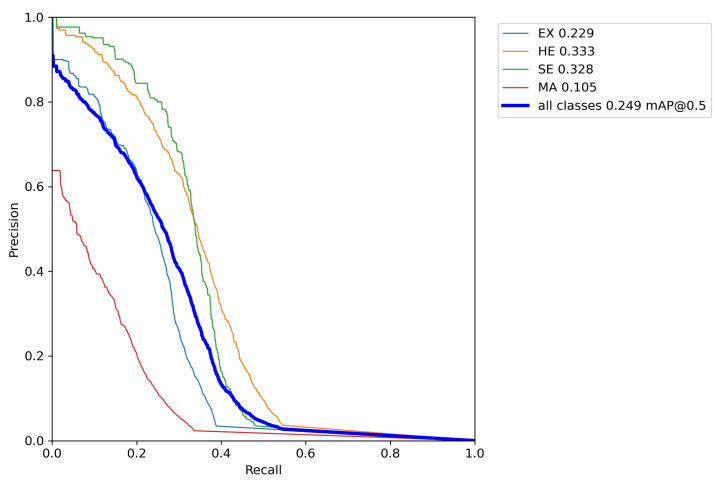
Graph with Precision×Recall curve with a limit of Intersection over Union of 0.5 obtained during the validation step of the proposed approach with Stochastic Gradient Descent (SGD) optimizer and *Tilling* in the Dataset for Diabetic Retinopathy (DDR). EX, hard exudates; HE, hemorrhages; SE, soft exudates; MA, microaneurysms; mAP, mean Average Precision.

Figure 5 presents the graph referring to the PR curve with a limit of IoU of 0.5 obtained during the validation stage using the proposed approach with the SGD optimizer and *Tilling* in the DDR dataset. The AP values obtained by the fundus lesions are plotted on the graph, according to the results presented in Table 4, whose *mean Average Precision* value obtained by all the classes corresponds to 0.249.

The *x* axis of the PR curve represents *Recall* while the *y* axis represents *Precision*. This curve mainly focuses on the performance of positive classes, which is critical when dealing with unbalanced classes. Thus, in the PR space, the goal is to be in the upper right corner (1,1), meaning that the predictor classified all positives as positive (Recall=1) and that everything that was classified as positive is true positive (Precision=1).

**Figure 6:**
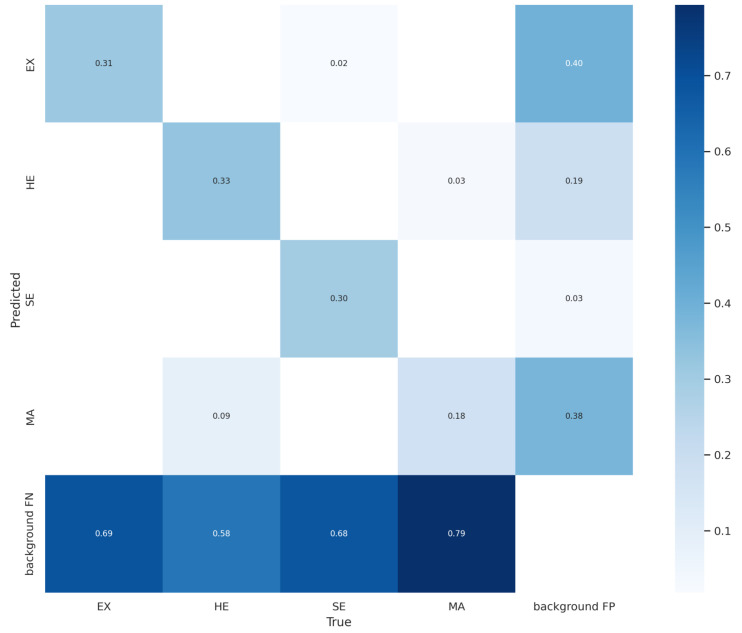
Confusion matrix obtained by the proposed approach with the Stochastic Gradient Descent (SGD) optimizer and *Tilling* during the validation step on the Dataset for Diabetic Retinopathy (DDR). EX, hard exudates; HE, hemorrhages; SE, soft exudates; MA, microaneurysms; FN, False Negative; FP, False Positive.

It is possible to verify, based on the analysis of the PR curve graph, that the proposed approach found greater difficulty in predicting Microaneurysms (MA) (red curve) followed by Hard Exudates (EX) (cyan curve), with the best results obtained with the prediction of Soft Exudates (SE) (green curve) and Hemorrhages (HE) (orange curve), respectively. The low precision obtained in the detection of MA is mainly related to the size of these microlesions and the gradient dissipation of these objects when the neural network is trained, causing a high rate of errors. This fact can be noted in the confusion matrix (as shown in Figure 6), with 79% of *background* FN and 38% of *background* FP, second only to hard exudates, with 40%. The fact that the proposed approach has achieved better results in predicting SE is associated with the morphological characteristics of these lesions since they generally have larger sizes than other lesions.

The confusion matrix is a table containing data from experiments with the proposed approach. Based on these data, it was possible to summarize the information related to the performance of the proposed approach and compare it with the results obtained with similar works in state-of-the-art. Figure 6 presents the confusion matrix obtained by the proposed approach with the SGD optimizer and *Tilling* during the validation step on the DDR dataset. It should be noted that the confusion matrix resulting from the detection of objects presents different characteristics when compared to problems that only involve the classification of objects in images since most model errors are associated with the background class and not with the other classes. In addition, the results presented in the confusion matrix will vary according to the defined confidence limit.

When detecting objects, it is common for information regarding false positives (FP) and false negatives (FN) to be presented in the confusion matrix (*background*). In this sense, the confidence limit established for detecting objects present in these images will directly impact the results obtained from *background* FP and *background* FN.

Therefore, the last row of the confusion matrix refers to *Ground Truth* objects that were not detected by the approach (*background* FN) and therefore considered as background. The last column of the confusion matrix is the detections performed by the approach that does not have any corresponding label in the *Ground Truth* (*background* FP), that is, the image background detected as a lesion.

A confidence limit is applied to filter the bounding boxes of a possible object to eliminate bounding boxes with low confidence scores through a Non-Maximum Suppression algorithm, which disregards detected objects with IoU less than the defined threshold. Thus, if a high confidence limit is defined, such as 0.90, there will be little confusion between the classes and low *background* FP results, but there will be a marked elimination of correctly detected fundus lesions (although with a low confidence limit), but with a confidence limit lower than 0.90. On the other hand, if a confidence limit of 0.25 is defined, there will be a more significant generation of *background* FP and *background* FN since it increases the probability of the model detecting the background as a lesion and vice versa.

Therefore, as the confidence limit tends to 1, the fund FPs will tend to 0. The results presented in the confusion matrix were calculated using a fixed confidence limit of 0.25, which is in line with the default inference configuration contained in the detect.py file of the proposed approach. In summary, with lower confidence limits, the results of mAP will be improved but will also produce a more significant amount of *background* FP that will appear in the confusion matrix, while if you increase the confidence limit, there will be a decrease in *background* FP in the confusion matrix, however, with a loss in mAP since more lesions are lost.

Cells with darker shades of blue indicate a greater number of samples. The confusion matrix presents the hits in the prediction of fundus lesions on the main diagonal, while the values off the main diagonal correspond to prediction errors. It is possible to verify that the highest incidence of *background* FN occurred in Microaneurysms (with 79%), followed by Hard Exudates (with 69%), Soft Exudates (with 68%), and Hemorrhages (with 58%). As for FP *background* errors, the highest incidence occurred in Hard Exudates (with 40%), followed by Microaneurysms (with 38%), Hemorrhages (with 19%), and Soft Exudates (with 3%). Also, it can be seen that 9% of Hemorrhages were incorrectly detected as Microanerysms, 2% of Soft Exudates were incorrectly detected as Hard Exudates, and 3% of Microaneurysms were incorrectly detected as Hemorrhages.

Thus, the results presented in the confusion matrix cannot be directly compared with the results of AP presented in this work, because the values are associated with the area under the PR curve, as shown in Figure 5. A good mAP produced by a low confidence limit, for example, will necessarily contain thousands of FPs, pushed to the lower right corner of the PR curve, with trends from *Recall* to 1 and *Precision* to 0, as shown in Figure 5.

During the test stage of the proposed approach in the DDR dataset using the SGD optimizer, the best result obtained reached a mAP of 0.1430 (indicated in bold font) and values of AP with a limit of IoU of 0.5 equal to 0.2100, 0.1380, 0.0530 and 0.1710, for Hard Exudates (EX), Soft Exudates (SE), Microaneurysms (MA) and Hemorrhages (HE), respectively, as presented in Table 5. Furthermore, both results obtained by the proposed approach, with and without the use of *Tilling*, achieved superior results to related works, which also detected fundus lesions in images from the test set of the DDR dataset.

Experiments were carried out during the validation stage of the proposed approach in the DDR dataset using the Adam optimizer, in which the best result was obtained using the *Tilling* method, with a mAP of 0.2630, and AP amounts with a IoU limit of 0.5 equal to 0.2240, 0.3650, 0.1110 and 0.3520, for Hard Exudates (EX), Soft Exudates (SE), Microaneurysms (MA) and Hemorrhages (HE), respectively, as shown in Table 6.

**Figure 7:**
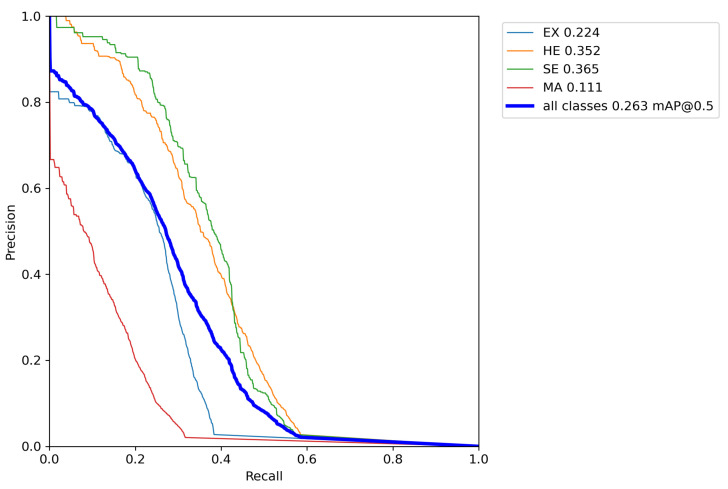
Graph of the Precision×Recall curve with a limit of Intersection over Union of 0.5 obtained during the validation step of the proposed approach with Adam optimizer and *Tilling* in the Dataset for Diabetic Retinopathy (DDR). EX, hard exudates; HE, hemorrhages; SE, soft exudates; MA, microaneurysms; mAP, mean Average Precision.

The use of the Adam optimizer resulted in a higher mAP than the result obtained by the proposed approach with the SGD optimizer, presented in Table 4. The proposed approach presented results superior to all works with the same purpose found in the literature. Figure 7 presents the graph of the PR curve with a limit of IoU of 0.5 obtained during the validation step using the proposed approach with the optimizer Adam and *Tilling* in the DDR dataset. The AP values obtained by the fundus lesions are plotted on the graph, according to the results presented in Table 6, whose *mean Average Precision* value obtained for all classes corresponds to 0.2630 (indicated in bold font). Analyzing the PR curve, it appears that using the Adam optimizer, the proposed approach presented similar results to those obtained using the SGD optimizer, i.e., there was a high rate of errors in detecting Microaneurysms (MA) (curve in red).

**Figure 8:**
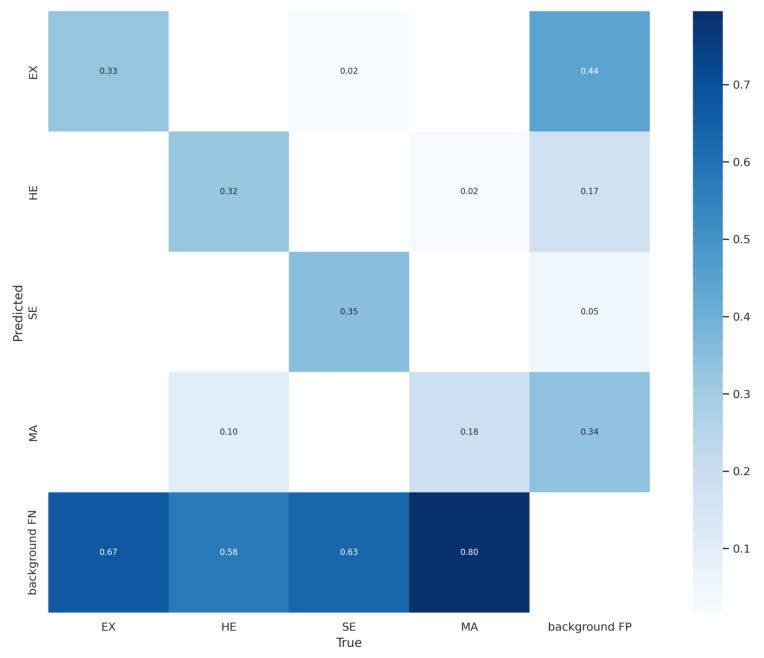
Confusion matrix obtained by the proposed approach with the Adam optimizer and *Tilling* during the validation step on the Dataset for Diabetic Retinopathy (DDR). EX, hard exudates; HE, hemorrhages; SE, soft exudates; MA, microaneurysms; FN, False Negative; FP, False Positive.

The best results were achieved in the prediction of Soft Exudates (SE) (curve in green color) and Hemorrhages (HE) (curve in orange color), respectively. It is possible to verify in the confusion matrix (as shown in Figure 8) the high rate of *background* FN (89%) and high rate of *background* FP (34%) of microaneurysms. The rate of *background* FP of hard exudates also stands out from the other classes of lesions, reaching 44%. The reasons that led to the high rates of FN and FP, both in detecting microaneurysms and hard exudates, have already been discussed.

Figure 8 presents the confusion matrix obtained by the proposed approach with the Adam optimizer and *Tilling* during the validation step on the DDR dataset. It is possible to verify that the highest incidence of *background* FN occurred in Microaneurysms (with 80%), followed by Hard Exudates (with 67%), Soft Exudates (with 63%), and Hemorrhages (with 58%). As for FP *background* errors, the highest incidence occurred in Hard Exudates (with 44%), followed by Microaneurysms (with 34%), Hemorrhages (with 17%), and Soft Exudates (with 5%). Also, it can be seen that 10% of Hemorrhages were incorrectly detected as Microaneurysms, 2% of Soft Exudates were incorrectly detected as Hard Exudates, and 2% of Microaneurysms were incorrectly detected as Hemorrhages.

**Figure 9:**
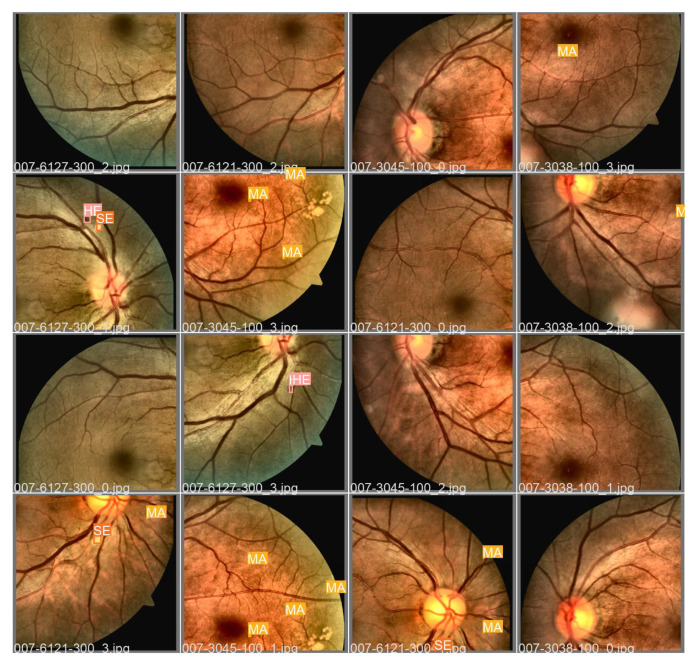
Batch example with fundus images of the Dataset for Diabetic Retinopathy (DDR) along with annotations (*Ground Truth*) of the fundus lesions after the pre-processing and data augmentation steps that were used to validate the proposed approach. MA, microaneurysms; HE, hemorrhages; SE, soft exudates.

**Figure 10:**
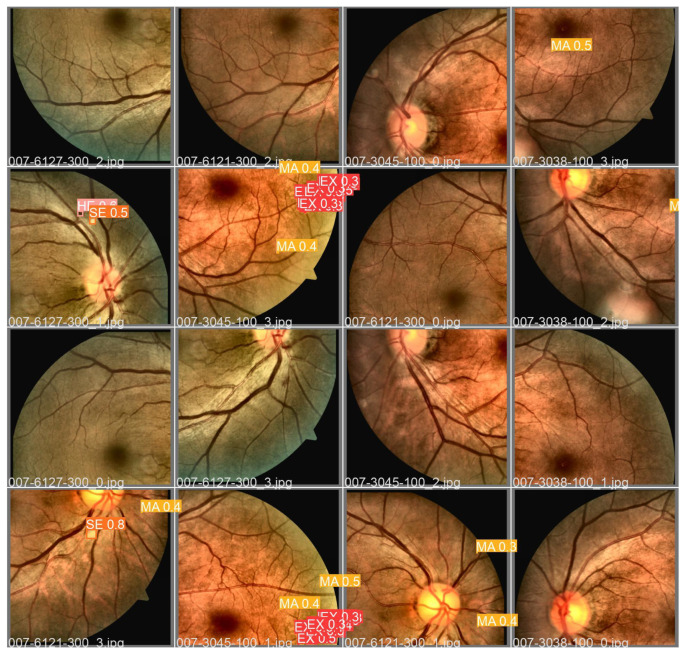
Batch with fundus images from the Dataset for Diabetic Retinopathy (DDR) with fundus lesions detected by the proposed approach during the validation step. MA, microaneurysms; HE, hemorrhages; SE, soft exudates; EX, hard exudates.

Figure 9 presents a batch with fundus images from the DDR dataset along with annotations (*Ground Truth*) of the fundus lesions after the preprocessing steps and augmentation of data that were used to validate the proposed approach using Adam optimizer and *Tilling*. Figure 10 shows the detections of fundus lesions performed on the same batch of fundus images described above.

The proposed approach was able to satisfactorily detect fundus lesions, such as the microaneurysm located in the image “007-3038-100_3.jpg”, or the hemorrhage and soft exudate in the image “007-6127-300_1.jpg”, or the microaneurysm and soft exudate in the image “007-6121-300_3.jpg”, and microaneurysms in the image “007-6121-300_1.jpg”. However, there were cases in which the proposed approach failed to detect the lesions or detected them wrongly, as in the case of one of the microaneurysms not located in the image “007-3045-100_3.jpg”, the hemorrhage in the image “007 -6127-300_3.jpg”, and two microaneurysms from image “007-3045-100_1.jpg”.

Even so, there were also situations where the proposed approach detected objects in the “007-3045-100_3.jpg” image as hard exudates, even though there were no annotations of these lesions in the *Ground Truth* of the DDR dataset. As this image presents microaneurysms in the same sector as the localized hard exudates, it is possible that these exudates were correctly detected, even though they were not originally located in the dataset. However, there is no way to confirm this information since only by verifying the absence of luminescence in these lesions, confirmed by image angiography (not available in the dataset), would it be possible to be sure about the diagnosis. In any case, based on the lesions detected and the generalization capacity verified after the predictions made on unknown images *a priori*, the proposed approach proved to be an essential tool to aid in medical diagnosis.

During the test stage of the proposed approach in the DDR dataset using the Adam optimizer, the best result obtained reached a mAP of 0.1540 (indicated in bold font) and values of AP with a limit of IoU of 0.5 equal to 0.2210, 0.1570, 0.0553 and 0.1840, for Hard Exudates (EX), Soft Exudates (SE), Microaneurysms (MA) and Hemorrhages (HE), respectively, as shown in Table 7. As in the validation set, the proposed approach (with and without the use of *Tilling*) obtained better results than the related works tested in the test set of the DDR dataset.

It is possible to verify that the optimization method that presented the best results was Adam. Thus, we can conclude that this optimizer has excellent potential for application in problems involving the detection of fundus lesions. However, in future works, we intend to conduct experiments with other optimization methods in state-of-the-art using different variations of hyperparameters.

The results obtained with the metrics are presented below: *Precision*, which considers, among all the positive classifications made by the model, how many are correct; the *Recall*, which assumes, among all situations of the positive class as expected value, how many are correct; and, the F1-*score*, which calculates the harmonic mean between Precision and Recall.

The best results achieved by the approach proposed in the DDR dataset were obtained using the Adam optimizer and the *Tilling* method, according to the F1-*score* metric obtained in the Validation and Testing stages, with values of 0.3485 (indicated in bold font) and 0.2521 (indicated in bold font), respectively, as shown in Table 8.

The mean inference time to detect fundus lesions per image in the DDR dataset in the Validation and Testing steps of the proposed approach is presented in Table 9. The approach proposed without *Tilling* had the lowest average inference time per image with the Adam optimizer, with 14.1 ms, while with *Tilling* the lowest average inference time per image was achieved with the SGD optimizer, with 4.6 ms (indicated in bold font). However, the highlight is the inference time of the proposed approach using *Tilling*, which was around 3 times faster than the inference time of the proposed approach applied to the images without performing from *Tilling*. Therefore, in addition to increasing the precision of the proposed approach in detecting fundus lesions, the *Tilling* method made the prediction process faster.

To assess the precision of the proposed approach in different public DR datasets, we also performed experiments with the Diabetic Retinopathy image set IDRiD [18]. During the validation step on the IDRiD dataset, the best result obtained by the proposed approach was using the SGD optimizer with the *Tilling* method, with a mAP of 0.3280 (indicated in bold font) and values of AP with a limit of IoU of 0.5 equal to 0.2630, 0.5340, 0.2170 and 0.2980, for Hard Exudates (EX), Soft Exudates (SE), Microaneurysms (MA) and Hemorrhages (HE), respectively, as shown in Table 10.

The best result of mAP obtained by the proposed approach during the validation using the IDRiD dataset surpassed the results obtained in the DDR dataset, thus attesting to the generalization capacity of the method adopted by the proposed approach for the detection of fundus lesions.

In the test stage of the proposed approach using the IDRiD dataset, the best result was obtained with the Adam optimizer and the use of *Tilling*, reaching a mAP of 0.2950 (indicated in bold font), and values of AP with a limit of IoU of 0.5 equal to 0.2530, 0.4090, 0.2210 and 0.2970, for Hard Exudates (EX), Soft Exudates (SE), Microaneurysms (MA) and Hemorrhages (HE), respectively, as shown in Table 11. The results obtained by the proposed approach in the test set of the IDRiD dataset were superior to those obtained in the test set of the DDR dataset.

**Figure 11:**
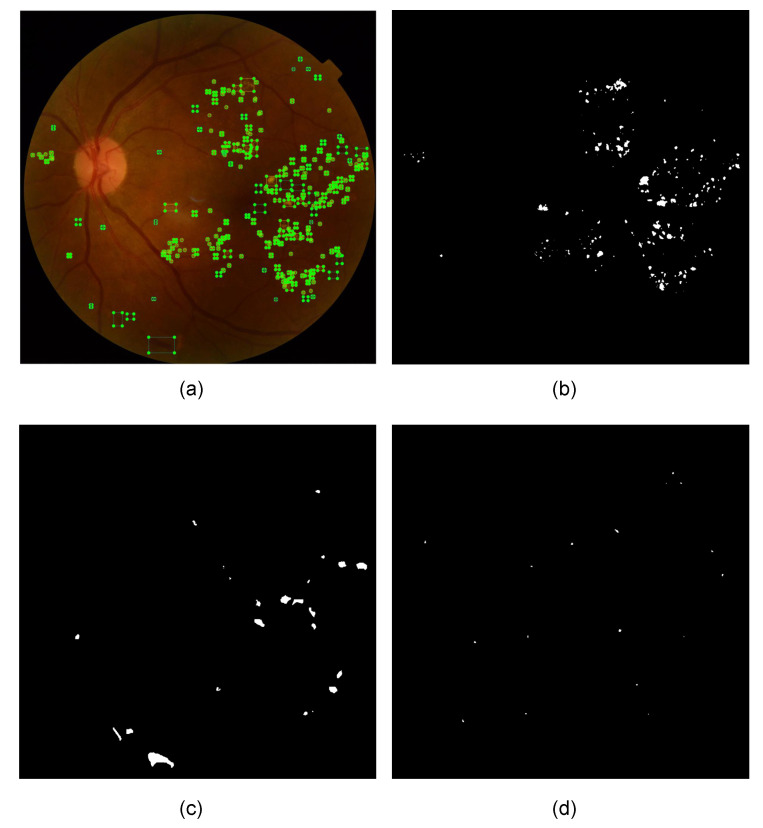
Example of fundus image of the dataset accompanied by the segmentation masks of the lesions present in the image. In (**a**), the fundus image “007-3711-200.jpg” of the test set from the Dataset for Diabetic Retinopathy (DDR), along with annotations (*Ground Truth*) of the fundus lesions; (**b**) segmentation mask of hard exudates; (**c**) hemorrhage segmentation mask; and (**d**) microaneurysm segmentation masks.

Figure 11a corresponds to the fundus image “007-3711-200.jpg” from the test set of the DDR dataset, along with the annotations (*Ground Truth*) of fundus lesions; Figure 11b is the segmentation mask of hard exudates; Figure 11c is the hemorrhage segmentation mask; and, Figure 11d is the segmentation mask of microaneurysms. It is important to note that there is no presence of soft exudates in this fundus image.

**Figure 12:**
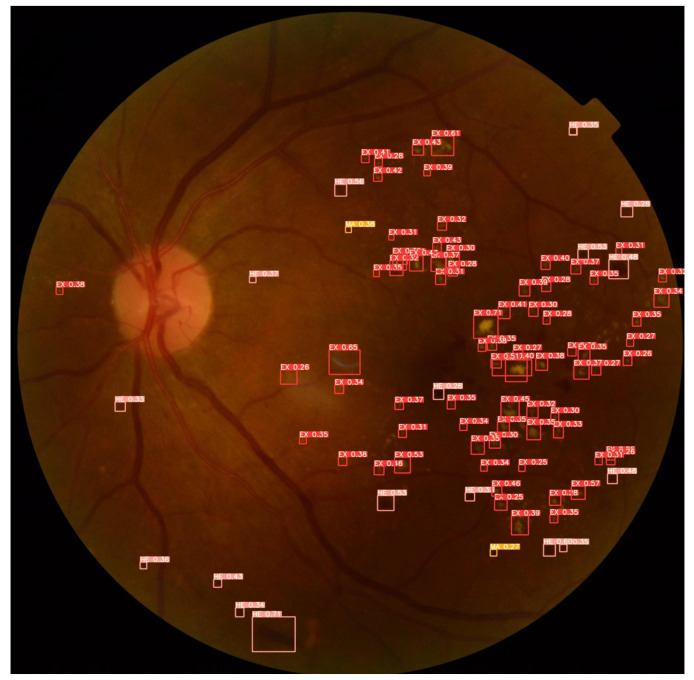
Detection of fundus lesions performed by the proposed approach and the percentage of confidence obtained in each object located in the fundus image “007-3711-200.jpg” of the test set of the Dataset for Diabetic Retinopathy (DDR). EX, hard exudates; HE, hemorrhages; MA, microaneurysms.

Figure 12 demonstrates the detection of fundus lesions performed by the proposed approach and the percentage of confidence obtained in each object located in the fundus image “007-3711-200.jpg” of the set test of the DDR dataset. This retinal image has a darker background, a recurrent feature in several fundus images of the investigated public datasets, which frequently causes problems in detecting lesions (mainly microaneurysms and hemorrhages). In addition, this feature generates high rates of errors during classification, in which a lesion is erroneously considered as background (*background* FN) or, conversely, where the background is erroneously considered as a lesion (*background* FP). For these cases, the image processing techniques applied in the pre-processing block of the proposed approach play an important role, as they aim to minimize these problems by reducing noise and improving the contrast of these images, for example.

Another aspect that can also be seen in this fundus image is the identified microlesions, as in the case of microaneurysms. These are extremely small lesions that end up making them challenging to detect. For these cases, the importance of, for example, the application of the *Tilling* method (pre-processing block of the proposed approach), the application of geometric transformations (data enhancement block of the proposed approach) and the architectural characteristics of the neural network of the proposed approach, such as the use of CSPs integrating *Backbone* and *Neck*, which aim to minimize problems of gradient dissipation of these microlesions during neural network training. Even so, with all these characteristics observed in this fundus image that make it challenging to identify the lesions, it is possible to verify that the proposed approach accurately performed the detection of most fundus lesions, localizing hard exudates (EX), hemorrhages (HE) and microaneurysms (MA) present in the image.

**Figure 13:**
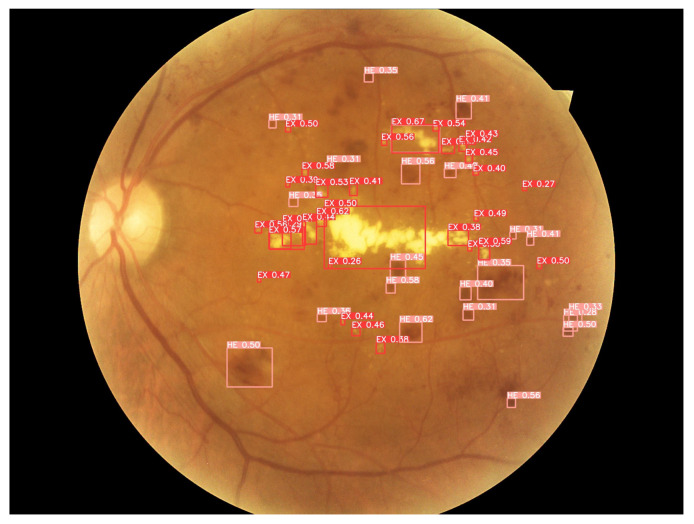
Detection of fundus lesions in the “007-3892-200.jpg” image of the test set of the Dataset for Diabetic Retinopathy (DDR). It is possible to observe different morphological aspects of the identified lesions, as in the case of hard exudates in the image’s central region and distributed in other regions of the retina, or hemorrhages, which, like the hard exudates detected. Also, they assume different shapes and sizes, in addition to being able to manifest themselves in different regions of the retina. EX, hard exudates; HE, hemorrhages.

Figure 13 shows the detection of fundus lesions in the “007-3892-200.jpg” image of the test set of the DDR dataset. With a higher level of detail, it is possible to observe the different morphological aspects of some identified lesions, such as hard exudates and Hemorrhages (that often produce classification errors due to similarities with microaneurysms). How shown the results presented in the confusion matrices of the experiments (Figure 6 and Figure 8).

## 5. Discussion

The works proposed by Alyoubi et al. [20] and Dai et al. [21] presented results in the validation set. Unlike these works, the same methodology as the work proposed by Li et al. [17] was adopted, which evaluated the proposed approach through the analysis of the results obtained both in the validation stage and in the test stage using the public dataset of DDR Diabetic Retinopathy. This method was adopted to avoid the evaluation of the proposed approach being carried out only in the validation set since this evaluation could give a false impression that the proposed approach is accurate in detecting fundus lesions.

Evaluating the approach on a validation set (where the neural network model is fitted) and then on a test set (where the data is not known *a priori*) allowed the generalization capability of the proposed approach to be properly verified, without the risk of biases produced by possible overfitting of the model during the validation. To validate the predictive capacity of the proposed approach regarding the detection of fundus lesions, we also evaluated it in the IDRiD dataset, in which we achieved results equivalent to those obtained in the DDR dataset.

The work by Dai et al. [21] was not compared with the proposed approach, as the authors used a 2-stage architecture while we used a single-stage architecture. The authors did not present the results of AP or mAP, unlike other works with a similar purpose found in the literature, which makes an adequate comparison impossible. In addition, the authors used a private DR dataset to train the deep learning models, which makes it difficult to reproducible the results obtained using the same method.

In the work proposed by Li et al. [17], the best results obtained regarding the detection of fundus lesions in the DDR dataset, using a Single-Stage model, was 0.0059 of mAP in the validation step with SSD and 0.0030 in the test step with YOLO. Santos et al. [59], using the DDR dataset, obtained a mAP of 0.0716 with the YOLOv4 model in the validation step. In work presented by Alyoubi et al. [20], the best result obtained by the authors in the validation step with the DDR dataset was a mAP of 0.1710, using the YOLOv3 model. The approach based on the YOLOv5 model proposed in this work, obtained a mAP of 0.2630 in the validation step and 0.1540 in the test step, both in DDR dataset.

With a confidence limit set at 0.25, the lesions were identified with their respective confidence percentages. The experimental results showed that the proposed approach obtained greater precision than similar works found in the literature. Another aspect observed during the experiments is that the proposed approach obtained greater precision in detecting Soft Exudates, Hemorrhages, and Hard Exudates, and, in contrast, a lower precision in detecting Microaneurysms was reached.

The detection of these lesions through computerized systems is a challenge due to numerous factors, among which: are the characteristics of size and shape of these lesions; noise and image contrast available in public DR datasets; the number of annotated examples of these lesions available in public DR datasets; and, the difficulty of deep learning algorithms in detecting very small objects. These problems were reported in the literature and observed during the experiments performed. Thus, a new image processing-based approach techniques and a state-of-the-art *Single-Stage* deep neural network architecture were proposed to overcome some of these problems to detect lesions in fundus images.

For the problem related to the shape and size of objects, in which very small lesions such as microaneurysms are more challenging to detect, techniques were applied to increase the receptive field of these lesions, such as partial *Cropping* of the black background of the images, and the *Tilling* of the input images for training the neural network. Also, a data augmentation technique based on the *Scale* geometric transformation was applied. In this case, a 50% zoom is randomly performed on the input images so that new images are artificially created for training. Thus, the neural network can extract more features, making it more efficient in detecting microlesions. A pre-processing block was developed in which we first filter the images to remove *outliers* from capturing these images and then apply the contrast-limited adaptive histogram equalization method to increase the local contrast of fundus images and improve lesion enhancement.

A block responsible for data augmentation was developed to minimize the problem of the small number of lesion examples noted in the public DR datasets. In this block, different state-of-the-art methods were applied, such as *Mosaic*, *MixUp*, *Copy-Paste* and geometric transformations (*flipping*, *scaling*, *perspective*, *translation* and *shearing*). The purpose of this step was to artificially create a greater number of example images with annotated lesions for training the proposed approach, to allow the deep neural network to extract a greater amount of lesion characteristics and, consequently, to increase the generalization capacity of the proposed approach.

In comparison to similar works found in the literature, it is essential to highlight the contributions of the proposed approach related to the structure of the deep neural network used, such as the use of CSP modules (C3) in the *Backbone* and *Neck* of the architecture, which minimized gradient dissipation problems, caused by the number of dense layers. In addition, through these modules, there was an improvement in inference speed and precision in lesion detection, as well as a reduction in computational cost and memory usage. Another innovation in the proposed approach’s structure was using the SiLU activation function throughout the neural network to simplify the architecture and reduce the number of hyperparameters. The *Threshold-Moving* method was applied during neural network training so that the image samples were weighed using a precision metric to minimize the imbalance in the number of examples of the different classes of lesions investigated and avoid classification biases in significant classes. Finally, the adjustments and tests were performed on the proposed approach through different public datasets on Diabetic Retinopathy. These datasets were split into Training, Validation, and Testing to evaluate the proposed approach according to the results of the different performance metrics.

The main limitation of our proposed approach focuses on identifying some lesions, as in the case of hard exudates, which have characteristics similar to drusen other than ocular signs caused by DR, for example. For this reason, the proposed approach made classification errors considering these drusen as hard exudates. Thus, a public dataset, with a broader range of eye signals, with lesions labeled and with a reasonable number of examples, associated or not with Diabetic Retinopathy, could support the training of deep learning models and the distinction more accurate of these different types of eye signals.

Another limitation observed during the experiments is associated with detecting microaneurysms. Although the results are better than similar works found in the literature, they are still low due to the size of these microlesions and have gradient dissipation problems. In this sense, we aimed to increase the detection accuracy of these microlesions by exploring architectures that perform the detection in two stages. Furthermore, we intend to explore different strategies, namely: (1) augmenting data to increase the number of examples of these microlesions; (2) improving the process of creating tiles from fundus images to provide the neural network images with the highest possible level of detail for extracting features from fundus lesions.

## 6. Conclusions

This article presented a new approach to fundus lesion detection using image processing techniques and a deep neural network based on a state-of-the-art YOLO architecture. Two public datasets of Diabetic Retinopathy images were used to train and evaluate the proposed approach’s precision: DDR and IDRiD. Only the images with annotated lesions in the datasets were used to perform the training and evaluation of the proposed approach. These datasets were partitioned into training, validation, and testing sets in a ratio of 50:20:30, respectively. The best results were achieved in the DDR dataset using the Adam optimizer and the *Tilling* method, reaching in the validation stage the mAP of 0.2630 for the limit of IoU of 0.5 and F1-*score* of 0.3485, and in the test step the mAP of 0.1540 for the limit of IoU of 0.5 and F1-*score* of 0.2521. The results obtained in the experiments demonstrate that the proposed approach presented results superior to equivalent works found in the literature.

The deep neural network architecture was implemented based on the YOLOv5 framework and the framework PyTorch, reaching 22.4% in the validation stage *Average Precision* for Hard Exudates, 36.5% for Soft Exudates, 11.1% for Microaneurysms, and 35.2% for Hemorrhages, in the DDR dataset. Different state-of-the-art image processing and data augmentation techniques were explored, such as CLAHE, *Tilling*, *Mosaic*, *Copy-Paste* and *MixUp*. In this way, it was possible to increase the precision of the proposed approach in detecting fundus lesions because, with the help of these techniques, the deep neural network architecture extracted a greater and more representative amount of characteristics of the lesions investigated during the training stage.

The experiments achieved state-of-the-art results, surpassing related works found in the literature with similar purpose and application and demonstrating that the detection of fundus lesions can be performed effectively through a deep learning-based approach. Furthermore, for the problem related to the size of objects, in which very small lesions are more difficult to detect, techniques were applied to increase the receptive field of these lesions, such as partial *Cropping* of the black background of the images and *Tilling* of the input images for training the neural network. However, the results presented in this work indicate that detecting microlesions such as microaneurysms in fundus images remains challenging for future research.

In future work, we intend to explore state-of-the-art architectures that perform instance segmentation to investigate and compare the trade-off between the precision and inference speed of these architectures with the approach proposed in this work. Furthermore, we intend to explore new data augmentation strategies and structures for the *Backbone*, *Neck* and *Head* of the deep neural network architecture implemented in the proposed approach, as well as to carry out experiments with other sets of public data on Diabetic Retinopathy.

## Figures and Tables

**Table 1:** Comparison between related work that use deep learning to classify, segment and detect objects in diabetic retinopathy images.

Ref.	Dataset	# Images	# of Images with Lesions Annotated				Data Augmentation	Unbalanced Data	Model	Performance Measure	Limitations
			MA	HE	EX	SE					
[18]	IDRiD	516	81	80	81	40	Applied	Yes	Mask R-CNN	IoU = 0.9338	EX, MA, SE e HE not detected
[17]	DDR	12,522	570	601	486	239	Not applied	Yes	SSD, YOLO	mAP = 0.0059 mAP = 0.0035	Low performance in detecting MA and SE
[20]							Applied	Yes	YOLOv3	mAP = 0.216	Imbalance of data used in training
[19]	DIARETDB1	89	80	54	48	36	Applied	Yes	CNN	Accuracy = 98.91%	MA e HE not detected
[21]	Private dataset	666,383	-	-	-	-	Not applied	Yes	Mask R-CNN	AUC = 0.954 AUC = 0.901 AUC = 0.941 AUC = 0.967	The dataset with fundus images used for training is private Validation of detection of lesions performed only in images from the private dataset

Definitions in Table 1: MA, microaneurysms; HE, hemorrhages; EX, hard exudates; SE, soft exudates; IDRiD, Indian Diabetic Retinopathy Image Dataset; DDR, Dataset for Diabetic Retinopathy; DIARETDB1, Standard Diabetic Retinopathy Database Calibration level 1; Mask R-CNN, Mask Regions with Convolutional Neural Network features; SSD, Detector MultiBox Single Shot; YOLO, You Only Look Once; YOLOv3, You Only Look Once version 3; CNN, Convolutional Neural Network; IoU, Intersection Over Union; mAP, mean Average Precision; AUC, Area Under The Curve.

**Table 2:** Number of annotated images for Microaneurysms, Hemorrhages, Hard Exudates, and Soft Exudates and the total amount of annotations by lesion type in the Dataset for Diabetic Retinopathy (DDR) before the data augmentation step.

# Images	Resolution	MA	HE	EX	SE	Notes of Lesions at *Pixel*	Multiple Experts
12,522	Variable	**# of images with lesion annotations**				Yes	Yes
		570	601	486	239		
		**# of lesion annotations**					
		10,388	13,093	23,713	1558		

Definitions in the Table 2: MA, microaneurysms; HE, hemorrhages; EX, hard exudates; SE, soft exudates.

**Table 3:** Hyperparameters adjusted during the validation step using the Dataset for Diabetic Retinopathy (DDR).

Parameters	Value
Batch Size	32
Number of Epochs	8000
Learning Rate	0.01
*Momentum*	0.937
Activation Function	SiLU
Optimizer	SGD and Adam
*Weight Decay*	0.0005
*Dropout*	10%
Threshold IoU NMS	0.45
Confidence Limit	0.25
Size of initial anchors (COCO)	(10, 13), (16, 30), (33, 23)—P3 (30, 61), (62, 45), (59, 119)—P4 (116, 90), (156, 198), (373, 326)—P5
Adjusted anchor size	(3, 3), (4, 4), (7, 7)—P3 (10, 10), ( 15, 15), (23, 28)—P4 (33, 24), (44, 49), (185, 124)—P5
*Early Stopping*	*Patience value* =100

Definitions in Table 3: SiLU, Sigmoid Linear Unit; SGD, Stochastic Gradient Descent; IoU NMS, Intersection Over Union Non-max Suppression; COCO, Common Objects in Context.

**Table 4:** Results obtained by the proposed approach with SGD optimizer compared to works related to the metrics AP and mAP for the limit of Intersection over Union of 0.5 in the validation set of the Dataset for Diabetic Retinopathy (DDR).

Models	AP				mAP
	EX	SE	MA	HE	
SSD [17]	0	0.0227	0	0.0007	0.0059
YOLO [17]	0.0039	0	0	0.0101	0.0035
YOLOv3+SGD [20]	-	-	-	-	0.1100
YOLOv3+SGD+*Dropout* [20]	-	-	-	-	0.1710
YOLOv4 [59]	0.0370	0.1493	0.0193	0.0849	0.0716
YOLOv5 (unmodified)	0.0306	0.2500	0.0047	0.1300	0.1040
Proposed Approach+SGD without *Tilling*	0.1490	0.4060	0.0454	0.2780	0.2200
**Proposed Approach+SGD with ** ***Tilling***	0.2290	0.3280	0.1050	0.3330	**0.2490**

Definitions in Table 4: AP, Average Precision; EX, hard exudates; SE, soft exudates; MA, microaneurysms; HE, hemorrhages; mAP, mean Average Precision; SSD, Single Shot MultiBox Detector; YOLO, You Only Look Once; YOLOv3, You Only Look Once version 3; YOLOv4, You Only Look Once version 4; YOLOv5, You Only Look Once version 5; SGD, Stochastic Gradient Descent.

**Table 5:** Results obtained by the proposed approach with SGD optimizer compared to works related to the metrics AP and mAP for the limit of Intersection over Union of 0.5 in the test set of the Dataset for Diabetic Retinopathy (DDR).

Models	AP				mAP
	EX	SE	MA	HE	
SSD [17]	0.0002	0	0.0001	0.0056	0.0015
YOLO [17]	0.0012	0	0	0.0109	0.0030
YOLOv5 (unmodified)	0.0342	0.1000	0.0028	0.0590	0.0511
Proposed Approach+SGD without *Tilling*	0.1430	0.2040	0.0280	0.1480	0.1310
**Proposed Approach+SGD with ** ***Tilling***	0.2100	0.1380	0.0530	0.1710	**0.1430**

Definitions in Table 5: AP, Average Precision; EX, hard exudates; SE, soft exudates; MA, microaneurysms; HE, hemorrhages; mAP, mean Average Precision; SSD, Single Shot MultiBox Detector; YOLO, You Only Look Once; YOLOv5, You Only Look Once version 5; SGD, Stochastic Gradient Descent.

**Table 6:** Results obtained by the proposed approach with Adam optimizer compared to works related to the metrics AP and mAP for the Intersection over Union limit of 0.5 in the validation set of the Dataset for Diabetic Retinopathy (DDR).

Models	AP				mAP
	EX	SE	MA	HE	
SSD [17]	0	0.0227	0	0.0007	0.0059
YOLO [17]	0.0039	0	0	0.0101	0.0035
YOLOv3+Adam+*Dropout* [20]	-	-	-	-	0.2160
Proposed Approach+Adam without *Tilling*	0.1640	0.4020	0.0610	0.3290	0.2390
**Proposed Approach+Adam with ** ***Tilling***	0.2240	0.3650	0.1110	0.3520	**0.2630**

Definitions in Table 6: AP, Average Precision; EX, hard exudates; SE, soft exudates; MA, microaneurysms; HE, hemorrhages; mAP, mean Average Precision; SSD, Single Shot MultiBox Detector; YOLO, You Only Look Once; YOLOv3, You Only Look Once version 3.

**Table 7:** Results obtained by the proposed approach with Adam optimizer compared to works related to the AP and mAP metrics for the Intersection over Union limit of 0.5 in the test set of the DDR (Dataset for Diabetic Retinopathy).

Models	AP				mAP
	EX	SE	MA	HE	
SSD [17]	0.0002	0	0.0001	0.0056	0.0015
YOLO [17]	0.0012	0	0	0.0109	0.0030
Proposed Approach+Adam without *Tilling*	0.1540	0.2110	0.0296	0.1590	0.1380
**Proposed Approach+Adam with ** ***Tilling***	0.2210	0.1570	0.0553	0.1840	**0.1540**

Definitions in Table 7: AP, Average Precision; EX, hard exudates; SE, soft exudates; MA, microaneurysms; HE, hemorrhages; mAP, mean Average Precision; SSD, Single Shot MultiBox Detector; YOLO, You Only Look Once.

**Table 8:** Results obtained with the metrics: *Precision*, *Recall* and F1-*score* with the Stochastic Gradient Descent (SGD) and Adam optimizers during the validation and testing steps using the Dataset for Diabetic Retinopathy (DDR).

Models	Precision Validation	Recall Validation	*F1-Score* Validation	Precision Test	Recall Test	*F1-Score* Test
Proposed Approach+SGD without *Tilling*	0.4533	0.2233	0.2992	0.3270	0.1540	0.2094
Proposed Approach+Adam without *Tilling*	0.4618	0.2484	0.3231	0.3060	0.1710	0.2194
Proposed Approach+SGD com *Tilling*	0.4775	0.2653	0.3411	0.3390	0.1820	0.2368
**Proposed Approach+Adam** **with ** ***Tilling***	0.4462	0.2859	**0.3485**	0.3410	0.2000	**0.2521**

**Table 9:** Mean inference time to detect fundus lesions per image in the Dataset for Diabetic Retinopathy (DDR) in the Validation and Testing stages of the proposed approach.

Models	Inference Time (ms) Validation	Inference Time (ms) Test
Proposed Approach+SGD without *Tilling*	15.7	13.0
Proposed Approach+Adam without *Tilling*	14.1	21.1
**Proposed Approach+SGD with ** ***Tilling***	**4.6**	5.9
Proposed Approach+Adam with *Tilling*	5.5	7.5

Definitions in Table 9: ms, millisecond; SGD, Stochastic Gradient Descent.

**Table 10:** Results obtained by the proposed approach with *Tilling* and the SGD and Adam optimizers with the metrics AP and mAP for the Intersection over Union limit of 0.5 in the validation set of the Indian Diabetic Retinopathy Image Dataset (IDRiD).

Models	AP				mAP
	EX	SE	MA	HE	
Proposed Approach+SGD without *Tilling*	0.1030	0.2940	0.0601	0.2460	0.1760
Proposed Approach+Adam without *Tilling*	0.1040	0.1810	0.0723	0.1350	0.1230
**Proposed Approach+SGD with ** ***Tilling***	0.2630	0.5340	0.2170	0.2980	**0.3280**
Proposed Approach+Adam with *Tilling*	0.2670	0.2740	0.2100	0.3200	0.2680

Definitions in Table 10: AP, Average Precision; EX, hard exudates; SE, soft exudates; MA, microaneurysms; HE, hemorrhages; mAP, mean Average Precision; SGD, Stochastic Gradient Descent.

**Table 11:** Results obtained by the proposed approach with *Tilling* and the SGD and Adam optimizers with the metrics AP and mAP for the Intersection over Union threshold of 0.5 in the test set of the Indian Diabetic Retinopathy Image Dataset (IDRiD).

Models	AP				mAP
	EX	SE	MA	HE	
Proposed Approach+SGD without *Tilling*	0.1260	0.3000	0.0787	0.2630	0.1920
Proposed Approach+Adam without *Tilling*	0.0993	0.2640	0.0661	0.1380	0.1420
Proposed Approach+SGD with *Tilling*	0.2390	0.3940	0.2010	0.2890	0.2810
**Proposed Approach+Adam with ** ***Tilling***	0.2530	0.4090	0.2210	0.2970	**0.2950**

Definitions in Table 11: AP, Average Precision; EX, hard exudates; SE, soft exudates; MA, microaneurysms; HE, hemorrhages; mAP, mean Average Precision; SGD, Stochastic Gradient Descent.

## Data Availability

We used publicly available datasets in this study. DDR dataset at https://doi.org/10.1016/j.ins.2019.06.011, accessed 9 May 2020 and IDRiD at https://ieee-dataport.org/open-access/indian-diabetic-retinopathy-image-dataset-idrid (accessed 5 August 2020).

## Supplementary Materials

The following supporting information can be downloaded at: https://www.mdpi.com/article/10.3390/s22176441/s1, Supplementary Materials: Pre-Processing, Image Preparation, and Data Augmentation in Details. References [105,106,107,108,109,110,111,112,113,114,115,116,117,118,119,120,121,122,123,124,125,126,127,128,129,130,131,132,133,134,135,136,137,138,139,140,141,142,143,144,145,146,147,148,149,150,151,152,153,154,155,156,157,158,159,160,161] are cited in Supplementary Materials File.

## References

[1] Delgado-Bonal A., Martín-Torres J. (2016). Human vision is determined based on information theory. Sci. Rep..

[2] Riordan-Eva P., Augsburger J.J. (2018). General Ophthalmology.

[3] IORJ (2021). O que é Retina. https://iorj.med.br/o-que-e-retina/.

[4] Mookiah M.R.K., Acharya U.R., Chua C.K., Lim C.M., Ng E.Y., Laude A. (2013). Computer-aided diagnosis of diabetic retinopathy: A review. Comput. Biol. Med..

[5] Yen G.G., Leong W.F. (2008). A sorting system for hierarchical grading of diabetic fundus images: A preliminary study. IEEE Trans. Inf. Technol. Biomed..

[6] Alghadyan A.A. (2011). Diabetic retinopathy—An update. Saudi J. Ophthalmol..

[7] ETDRSR (1991). Grading Diabetic Retinopathy from Stereoscopic Color Fundus Photographs—An Extension of the Modified Airlie House Classification. Ophthalmology.

[8] Philip S., Fleming A.D., Goatman K.A., Fonseca S., Mcnamee P., Scotland G.S., Prescott G.J., Sharp P.F., Olson J.A. (2007). The efficacy of automated “disease/no disease” grading for diabetic retinopathy in a systematic screening programme. Br. J. Ophthalmol..

[9] ETDRSR (1991). Classification of Diabetic Retinopathy from Fluorescein Angiograms. Ophthalmology.

[10] Hendrick A.M., Gibson M.V., Kulshreshtha A. (2015). Diabetic Retinopathy. Prim. Care-Clin. Off. Pract..

[11] Williams R., Airey M., Baxter H., Forrester J., Kennedy-Martin T., Girach A. (2004). Epidemiology of diabetic retinopathy and macular oedema: A systematic review. Eye.

[12] International Council of Ophthalmology (2017). Updated 2017 ICO Guidelines for Diabetic Eye Care. ICO Guidelines for Diabetic Eye Care.

[13] Cardoso C.d.F.d.S. (2019). Segmentação Automática do Disco óptico e de vasos Sanguíneos em Imagens de Fundo de Olho. Ph.D. Thesis.

[14] Lecaire T.J., Palta M., Klein R., Klein B.E., Cruickshanks K.J. (2013). Assessing progress in retinopathy outcomes in type 1 diabetes. Diabetes Care.

[15] Chakrabarti R., Harper C.A., Keeffe J.E. (2012). Diabetic retinopathy management guidelines. Expert Rev. Ophthalmol..

[16] Vocaturo E., Zumpano E. The contribution of AI in the detection of the Diabetic Retinopathy. Proceedings of the—2020 IEEE International Conference on Bioinformatics and Biomedicine, BIBM 2020.

[17] Li T., Gao Y., Wang K., Guo S., Liu H., Kang H. (2019). Diagnostic assessment of deep learning algorithms for diabetic retinopathy screening. Inf. Sci..

[18] Porwal P., Pachade S., Kokare M., Deshmukh G., Son J., Bae W., Liu L., Wang J., Liu X., Gao L. (2020). IDRiD: Diabetic Retinopathy—Segmentation and Grading Challenge. Med. Image Anal..

[19] Mateen M., Wen J., Nasrullah N., Sun S., Hayat S. (2020). Exudate Detection for Diabetic Retinopathy Using Pretrained Convolutional Neural Networks. Complexity.

[20] Alyoubi W.L., Abulkhair M.F., Shalash W.M. (2021). Diabetic Retinopathy Fundus Image Classification and Lesions Localization System Using Deep Learning. Sensors.

[21] Dai L., Wu L., Li H., Cai C., Wu Q., Kong H., Liu R., Wang X., Hou X., Liu Y. (2021). A deep learning system for detecting diabetic retinopathy across the disease spectrum. Nat. Commun..

[22] Simonyan K., Zisserman A. Very deep convolutional networks for large-scale image recognition. Proceedings of the 3rd International Conference on Learning Representations, ICLR 2015—Conference Track Proceedings.

[23] He K., Zhang X., Ren S., Sun J. Deep residual learning for image recognition. Proceedings of the IEEE Computer Society Conference on Computer Vision and Pattern Recognition.

[24] Szegedy C., Liu W., Jia Y., Sermanet P., Reed S., Anguelov D., Erhan D., Vanhoucke V., Rabinovich A. Going deeper with convolutions. Proceedings of the 2015 IEEE Conference on Computer Vision and Pattern Recognition (CVPR).

[25] Huang G., Liu Z., Van Der Maaten L., Weinberger K.Q. Densely connected convolutional networks. Proceedings of the—30th IEEE Conference on Computer Vision and Pattern Recognition, CVPR 2017.

[26] Hu J., Shen L., Sun G. Squeeze-and-Excitation Networks. Proceedings of the 2018 IEEE/CVF Conference on Computer Vision and Pattern Recognition.

[27] Xie S., Tu Z. Holistically-nested edge detection. Proceedings of the IEEE International Conference on Computer Vision.

[28] Chen L.C., Zhu Y., Papandreou G., Schroff F., Adam H. (2018). Encoder-decoder with atrous separable convolution for semantic image segmentation. Lect. Notes Comput. Sci..

[29] Konishi Y., Hanzawa Y., Kawade M., Hashimoto M. (2016). SSD: Single Shot MultiBox Detector. Eccv.

[30] Redmon J., Divvala S., Girshick R., Farhadi A. You only look once: Unified, real-time object detection. Proceedings of the IEEE Computer Society Conference on Computer Vision and Pattern Recognition.

[31] Melo R., Lima G., Corrêa G., Zatt B., Aguiar M., Nachtigall G., Araújo R., Cerri R., Prati R.C. (2020). Diagnosis of Apple Fruit Diseases in the Wild with Mask R-CNN. Intelligent Systems.

[32] Ronneberger O., Fischer P., Brox T. (2015). U-net: Convolutional networks for biomedical image segmentation. Lect. Notes Comput. Sci..

[33] Shelhamer E., Long J., Darrell T. (2017). Fully Convolutional Networks for Semantic Segmentation. IEEE Trans. Pattern Anal. Mach. Intell..

[34] Yu F., Wang D., Shelhamer E., Darrell T. Deep Layer Aggregation. Proceedings of the IEEE Computer Society Conference on Computer Vision and Pattern Recognition.

[35] Szegedy C., Vanhoucke V., Ioffe S., Shlens J., Wojna Z. Rethinking the Inception Architecture for Computer Vision. Proceedings of the IEEE Computer Society Conference on Computer Vision and Pattern Recognition.

[36] Tsiknakis N., Theodoropoulos D., Manikis G., Ktistakis E., Boutsora O., Berto A., Scarpa F., Scarpa A., Fotiadis D.I., Marias K. (2021). Deep learning for diabetic retinopathy detection and classification based on fundus images: A review. Comput. Biol. Med..

[37] Redmon J., Farhadi A. (2018). YOLOv3: An Incremental Improvement. arXiv.

[38] He K., Gkioxari G., Dollár P., Girshick R. (2020). Mask R-CNN. IEEE Trans. Pattern Anal. Mach. Intell..

[39] Ramcharan A., McCloskey P., Baranowski K., Mbilinyi N., Mrisho L., Ndalahwa M., Legg J., Hughes D. (2018). Assessing a mobile-based deep learning model for plant disease surveillance. arXiv.

[40] Ren S., He K., Girshick R., Sun J. (2017). Faster R-CNN: Towards Real-Time Object Detection with Region Proposal Networks. IEEE Trans. Pattern Anal. Mach. Intell..

[41] Ojha A., Sahu S.P., Dewangan D.K. Vehicle Detection through Instance Segmentation using Mask R-CNN for Intelligent Vehicle System. Proceedings of the 5th International Conference on Intelligent Computing and Control Systems (ICICCS).

[42] Iacovacci J., Wu Z., Bianconi G. (2015). Mesoscopic structures reveal the network between the layers of multiplex data sets. Phys. Rev.-Stat. Nonlinear Soft Matter Phys..

[43] Bertels J., Eelbode T., Berman M., Vandermeulen D., Maes F., Bisschops R., Blaschko M.B. (2019). Optimizing the Dice Score and Jaccard Index for Medical Image Segmentation: Theory and Practice. Lect. Notes Comput. Sci..

[44] Kaggle (2015). Diabetic Retinopathy Detection. https://www.kaggle.com/c/diabetic-retinopathy-detection.

[45] Zhu L., Geng X., Li Z., Liu C. (2021). Improving YOLOv5 with Attention Mechanism for Detecting Boulders from Planetary Images. Remote Sens..

[46] Xu R., Lin H., Lu K., Cao L., Liu Y. (2021). A forest fire detection system based on ensemble learning. Forests.

[47] Qi D., Tan W., Yao Q., Liu J. (2021). YOLO5Face: Why Reinventing a Face Detector. arXiv.

[48] Zhu X., Lyu S., Wang X., Zhao Q. TPH-YOLOv5: Improved YOLOv5 Based on Transformer Prediction Head for Object Detection on Drone-captured Scenarios. Proceedings of the IEEE/CVF International Conference on Computer Vision.

[49] Rahman R., Azad Z.B., Hasan M.B. Densely-Populated Traffic Detection using YOLOv5 and Non-Maximum Suppression Ensembling. Proceedings of the International Conference on Big Data, IoT, and Machine Learning.

[50] Zheng Z., Zhao J., Li Y. (2021). Research on Detecting Bearing-Cover Defects Based on Improved YOLOv3. IEEE Access.

[51] Xie J., Zheng S. (2021). ZSD-YOLO: Zero-Shot YOLO Detection using Vision-Language KnowledgeDistillation. arXiv.

[52] Solawetz J. (2020). YOLOv5: The Latest Model for Object Detection. YOLOv5 New Version—Improvements and Evaluation. https://blog.roboflow.com/yolov5-improvements-and-evaluation/.

[53] Couturier R., Noura H.N., Salman O., Sider A. (2021). A Deep Learning Object Detection Method for an Efficient Clusters Initialization. arXiv.

[54] Li J., Guo S., Kong L., Tan S., Yuan Y. (2021). An improved YOLOv3-tiny method for fire detection in the construction industry. E3S Web Conf..

[55] Bochkovskiy A., Wang C.Y., Liao H.Y.M. (2020). YOLOv4: Optimal Speed and Accuracy of Object Detection. arXiv.

[56] Walter T., Klein J.C., Massin P., Erginay A. (2002). A contribution of image processing to the diagnosis of diabetic retinopathy—Detection of exudates in color fundus images of the human retina. IEEE Trans. Med. Imaging.

[57] Jasim M.K., Najm R., Kanan E.H., Alfaar H.E., Otair M. (2019). Image Noise Removal Techniques: A Comparative Analysis. http://www.warse.org/IJSAIT/static/pdf/file/ijsait01862019.pdf.

[58] Gonzalez R., Woods R. (2010). Processamento Digital de Imagens.

[59] Santos C., De Aguiar M.S., Welfer D., Belloni B. Deep Neural Network Model based on One-Stage Detector for Identifying Fundus Lesions. Proceedings of the 2021 International Joint Conference on Neural Networks (IJCNN).

[60] Rai R., Gour P., Singh B. (2012). Underwater Image Segmentation using CLAHE Enhancement and Thresholding. Int. J. Emerg. Technol. Adv. Eng..

[61] Horry M.J., Chakraborty S., Paul M., Ulhaq A., Pradhan B., Saha M., Shukla N. (2020). COVID-19 Detection Through Transfer Learning Using Multimodal Imaging Data. IEEE Access.

[62] El abbadi N., Hammod E. (2014). Automatic Early Diagnosis of Diabetic Retinopathy Using Retina Fundus Images Enas Hamood Al-Saadi-Automatic Early Diagnosis of Diabetic Retinopathy Using Retina Fundus Images. Eur. Acad. Res..

[63] Nguyen T.S., Stueker S., Niehues J., Waibel A. Improving sequence-to-sequence speech recognition training with on-the-fly data augmentation. Proceedings of the IEEE International Conference on Acoustics, Speech and Signal Processing (ICASSP).

[64] Lam T.K., Ohta M., Schamoni S., Riezler S. (2021). On-the-Fly Aligned Data Augmentation for Sequence-to-Sequence ASR. arXiv.

[65] Liu C., Jin S., Wang D., Luo Z., Yu J., Zhou B., Yang C. (2020). Constrained Oversampling: An Oversampling Approach to Reduce Noise Generation in Imbalanced Datasets with Class Overlapping. IEEE Access.

[66] Japkowicz N. Learning from imbalanced data sets: A comparison of various strategies. Proceedings of the AAAI Workshop on Learning from Imbalanced Data Sets.

[67] Provost F. Machine learning from imbalanced data sets 101. Proceedings of the AAAI’2000 Workshop on Imbalanced Data Sets.

[68] Zhou Z.H., Liu X.Y. (2006). Training cost-sensitive neural networks with methods addressing the class imbalance problem. IEEE Trans. Knowl. Data Eng..

[69] Buda M., Maki A., Mazurowski M.A. (2018). A systematic study of the class imbalance problem in convolutional neural networks. Neural Netw..

[70] Zhang X., Gweon H., Provost S. (2020). Threshold Moving Approaches for Addressing the Class Imbalance Problem and their Application to Multi-label Classification. Pervasivehealth Pervasive Comput. Technol. Healthc..

[71] He H., Garcia E.A. (2009). Learning from Imbalanced Data. IEEE Trans. Knowl. Data Eng..

[72] Fernández A., García S., Galar M., Prati R.C. (2019). Learning from Imbalanced Data Sets.

[73] Ge Z., Liu S., Wang F., Li Z., Sun J. (2021). YOLOX: Exceeding YOLO Series in 2021. arXiv.

[74] Iyer R., Shashikant Ringe P., Varadharajan Iyer R., Prabhulal Bhensdadiya K. (2021). Comparison of YOLOv3, YOLOv5s and MobileNet-SSD V2 for Real-Time Mask Detection Comparison of YOLOv3, YOLOv5s and MobileNet-SSD V2 for Real-Time Mask Detection View project Comparison of YOLOv3, YOLOv5s and MobileNet-SSD V2 for Real-Time Mask Detection. Artic. Int. J. Res. Eng. Technol..

[75] Yu Y., Zhao J., Gong Q., Huang C., Zheng G., Ma J. (2021). Real-Time Underwater Maritime Object Detection in Side-Scan Sonar Images Based on Transformer-YOLOv5. Remote Sens..

[76] Wang C.Y., Mark Liao H.Y., Wu Y.H., Chen P.Y., Hsieh J.W., Yeh I.H. CSPNet: A new backbone that can enhance learning capability of CNN. Proceedings of the IEEE Computer Society Conference on Computer Vision and Pattern Recognition Workshops.

[77] Xie S., Girshick R., Dollár P., Tu Z., He K. Aggregated residual transformations for deep neural networks. Proceedings of the 30th IEEE Conference on Computer Vision and Pattern Recognition, CVPR 2017.

[78] Ioffe S., Szegedy C. Batch Normalization: Accelerating Deep Network Training by Reducing Internal Covariate Shift. Proceedings of the International Conference on Machine Learning.

[79] Elfwing S., Uchibe E., Doya K. (2017). Sigmoid-Weighted Linear Units for Neural Network Function Approximation in Reinforcement Learning. Neural Netw..

[80] Agarap A.F. (2019). Deep Learning using Rectified Linear Units (ReLU). arXiv.

[81] He K., Zhang X., Ren S., Sun J. (2014). Spatial Pyramid Pooling in Deep Convolutional Networks for Visual Recognition. Lect. Notes Comput. Sci..

[82] Zeiler M.D., Taylor G.W., Fergus R. Adaptive deconvolutional networks for mid and high level feature learning. Proceedings of the 2011 International Conference on Computer Vision.

[83] Li X., Lai T., Wang S., Chen Q., Yang C., Chen R. Feature Pyramid Networks for Object Detection. Proceedings of the 2019 IEEE International Conference on Parallel and Distributed Processing with Applications, Big Data and Cloud Computing, Sustainable Computing and Communications, Social Computing and Networking, ISPA/BDCloud/SustainCom/SocialCom 2019.

[84] Liu S., Qi L., Qin H., Shi J., Jia J. Path Aggregation Network for Instance Segmentation. Proceedings of the IEEE Computer Society Conference on Computer Vision and Pattern Recognition.

[85] Jadon S. A survey of loss functions for semantic segmentation. Proceedings of the 2020 IEEE Conference on Computational Intelligence in Bioinformatics and Computational Biology, CIBCB 2020.

[86] Rezatofighi H., Tsoi N., Gwak J., Sadeghian A., Reid I., Savarese S. Generalized intersection over union: A metric and a loss for bounding box regression. Proceedings of the IEEE Computer Society Conference on Computer Vision and Pattern Recognition.

[87] Lin K., Zhao H., Lv J., Zhan J., Liu X., Chen R., Li C., Huang Z. (2019). Face Detection and Segmentation with Generalized Intersection over Union Based on Mask R-CNN. Advances in Brain Inspired Cognitive Systems, Proceedings of the 10th International Conference, BICS 2019, Guangzhou, China, 13–14 July 2019.

[88] Oksuz K., Cam B.C., Kahraman F., Baltaci Z.S., Kalkan S., Akbas E. (2021). Mask-aware IoU for Anchor Assignment in Real-time Instance Segmentation. arXiv.

[89] Pan S.J., Yang Q. (2010). A survey on transfer learning. IEEE Trans. Knowl. Data Eng..

[90] Blitzer J., Dredze M., Pereira F. (2007). Biographies, Bollywood, Boom-boxes and Blenders: Domain Adaptation for Sentiment Classification. Proceedings of the 45th Annual Meeting of the Association of Computational Linguistics.

[91] Lin T.Y., Maire M., Belongie S., Hays J., Perona P., Ramanan D., Dollár P., Zitnick C.L. (2014). Microsoft COCO: Common objects in context. Lect. Notes Comput. Sci..

[92] Franke M., Gopinath V., Reddy C., Ristić-Durrant D., Michels K. Bounding Box Dataset Augmentation for Long-Range Object Distance Estimation. Proceedings of the IEEE/CVF International Conference on Computer Vision (ICCV) Workshops.

[93] Mamdouh N., Khattab A. (2021). YOLO-Based Deep Learning Framework for Olive Fruit Fly Detection and Counting. IEEE Access.

[94] Dewi C., Chen R.C., Liu Y.T., Jiang X., Hartomo K.D. (2021). Yolo V4 for Advanced Traffic Sign Recognition with Synthetic Training Data Generated by Various GAN. IEEE Access.

[95] Freitas G.A.d.L. (2019). Aprendizagem Profunda Aplicada ao Futebol de Robôs: Uso de Redes Neurais Convolucionais para Detecção de Objetos Universidade Estadual de Londrina Centro de Tecnologia e Urbanismo Departamento de Engenharia Elétrica Aprendizagem Profunda Aplicada ao Fute.

[96] COCO (2021). Detection Evaluation Metrics Used by COCO. https://cocodataset.org/#detection-eval.

[97] Prechelt L. (1998). Early Stopping—But When?.

[98] Zhang C., Bengio S., Hardt M., Recht B., Vinyals O. (2017). Understanding deep learning requires rethinking generalization. arXiv.

[99] Srivastava N., Hinton G., Krizhevsky A., Sutskever I., Salakhutdinov R. (2014). Dropout: A Simple Way to Prevent Neural Networks from Overfitting. J. Mach. Learn. Res..

[100] Liang X., Wu L., Li J., Wang Y., Meng Q., Qin T., Chen W., Zhang M., Liu T.Y. (2021). R-Drop: Regularized Dropout for Neural Networks. Adv. Neural Inf. Process. Syst..

[101] Labach A., Salehinejad H., Valaee S. (2019). Survey of Dropout Methods for Deep Neural Networks. arXiv.

[102] Davis J., Goadrich M. The relationship between Precision-Recall and ROC curves. Proceedings of the ICML 2006—Proceedings of the 23rd International Conference on Machine Learning.

[103] Manning C.D., Raghavan P., Schütze H. (2008). Introduction to Information Retrieval.

[104] Flach P.A., Kull M. (2015). Precision-Recall-Gain curves: PR analysis done right. Adv. Neural Inf. Process. Syst..

[105] Asamoah D., Ofori E., Opoku S., Danso J. (2018). Measuring the Performance of Image Contrast Enhancement Technique. Int. J. Comput. Appl..

[106] Bodla N., Singh B., Chellappa R., Davis L.S. Soft-NMS - Improving Object Detection with One Line of Code. Proceedings of the IEEE International Conference on Computer Vision.

[107] Carratino L., Cissé M., Jenatton R., Vert J.P. (2020). On Mixup Regularization. arXiv.

[108] Castro D.J.L. (1996). Garra Servo-Controlada com Integração de Informação táCtil e de Proximidade. Master’s Thesis.

[109] Chandrasekar L., Durga G. Implementation of Hough Transform for image processing applications. Proceedings of the 2014 International Conference on Communication and Signal Processing.

[110] Claro M., Vogado L., Santos J., Veras R. (2020). Utilização de Técnicas de Data Augmentation em Imagens: Teoria e Prática. https://sol.sbc.org.br/livros/index.php/sbc/catalog/view/48/224/445-1.

[111] Li F.-F., Krishna R., Xu D. (2021). cs231n, Lecture 15—Slide 4, Detection and Segmentation. http://cs231n.stanford.edu/slides/2021/lecture_15.pdf.

[112] Li F.-F., Deng J., Li K. (2010). ImageNet: Constructing a large-scale image database. J. Vis..

[113] Dai F., Fan B., Peng Y. An image haze removal algorithm based on blockwise processing using LAB color space and bilateral filtering. Proceedings of the 2018 Chinese Control And Decision Conference (CCDC).

[114] Dai J., Li Y., He K., Sun J. (2016). R-FCN: Object Detection via Region-Based Fully Convolutional Networks. arXiv.

[115] dos Santos J.R.V. (2016). Avaliação de Técnicas de Realce de Imagens Digitais Utilizando Métricas Subjetivas e Objetivas. Master’s Thesis.

[116] Dvornik N., Mairal J., Schmid C. (2018). Modeling Visual Context is Key to Augmenting Object Detection Datasets. arXiv.

[117] Dwibedi D., Misra I., Hebert M. (2017). Cut, Paste and Learn: Surprisingly Easy Synthesis for Instance Detection. arXiv.

[118] Erfurt J., Helmrich C.R., Bosse S., Schwarz H., Marpe D., Wiegand T. A Study of the Perceptually Weighted Peak Signal-To-Noise Ratio (WPSNR) for Image Compression. Proceedings of the 2019 IEEE International Conference on Image Processing (ICIP).

[119] Fardo F.A., Conforto V.H., de Oliveira F.C., Rodrigues P.S. (2016). A Formal Evaluation of PSNR as Quality Measurement Parameter for Image Segmentation Algorithms. arXiv.

[120] Faria D. (2010). Trabalhos Práticos Análise e Processamento de Imagem.

[121] Ghiasi G., Cui Y., Srinivas A., Qian R., Lin T.Y., Cubuk E.D., Le Q.V., Zoph B. (2021). Simple Copy-Paste is a Strong Data Augmentation Method for Instance Segmentation. arXiv.

[122] Girshick R., Donahue J., Darrell T., Malik J. Rich feature hierarchies for accurate object detection and semantic segmentation. Proceedings of the IEEE Computer Society Conference on Computer Vision and Pattern Recognition.

[123] Girshick R. Fast R-CNN. Proceedings of the IEEE International Conference on Computer Vision, ICCV 2015.

[124] Gonzalez R.C., Woods R.E., Eddins S.L. (2003). Digital Image Processing Using MATLAB.

[125] Guo H., Mao Y., Zhang R. (2019). Augmenting Data with Mixup for Sentence Classification: An Empirical Study. arXiv.

[126] Guo H., Mao Y., Zhang R. (2018). MixUp as Locally Linear Out-Of-Manifold Regularization. arXiv.

[127] Hao R., Namdar K., Liu L., Haider M.A., Khalvati F. (2020). A Comprehensive Study of Data Augmentation Strategies for Prostate Cancer Detection in Diffusion-weighted MRI using Convolutional Neural Networks. arXiv.

[128] Hawas A.R., Ashour A.S., Guo Y., Guo Y., Ashour A.S. (2019). 8—Neutrosophic set in medical image clustering. Neutrosophic Set in Medical Image Analysis.

[129] Huynh-The T., Le B.V., Lee S., Le-Tien T., Yoon Y. (2014). Using weighted dynamic range for histogram equalization to improve the image contrast. EURASIP J. Image Video Process..

[130] Illingworth J., Kittler J. (1987). The Adaptive Hough Transform. IEEE Trans. Pattern Anal. Mach. Intell..

[131] Kim J.H., Choo W., Jeong H., Song H.O. (2021). Co-Mixup: Saliency Guided Joint Mixup with Supermodular Diversity. arXiv.

[132] Liu Z., Chen W., Zou Y., Hu C. Regions of interest extraction based on HSV color space. Proceedings of the IEEE 10th International Conference on Industrial Informatics.

[133] Ma J., Fan X., Yang S.X., Zhang X., Zhu X. (2018). Contrast Limited Adaptive Histogram Equalization-Based Fusion in YIQ and HSI Color Spaces for Underwater Image Enhancement. Int. J. Pattern Recognit. Artif. Intell..

[134] Marroni L.S. (2002). Aplicação da Transformada de Hough Para Localização dos Olhos em Faces Humanas. Master’s Thesis.

[135] McREYNOLDS T., BLYTHE D., McReynolds T., Blythe D. (2005). CHAPTER 12—Image Processing Techniques. Advanced Graphics Programming Using OpenGL.

[136] Mukhopadhyay S., Mandal S., Pratiher S., Changdar S., Burman R., Ghosh N., Panigrahi P.K. (2015). A comparative study between proposed Hyper Kurtosis based Modified Duo-Histogram Equalization (HKMDHE) and Contrast Limited Adaptive Histogram Equalization (CLAHE) for Contrast Enhancement Purpose of Low Contrast Human Brain CT scan images. arXiv.

[137] Nixon M.S., Aguado A.S., Nixon M.S., Aguado A.S. (2020). 5—High-level feature extraction: Fixed shape matching. Feature Extraction and Image Processing for Computer Vision.

[138] Paris S., Durand F., Leonardis A., Bischof H., Pinz A. (2006). A Fast Approximation of the Bilateral Filter Using a Signal Processing Approach. Proceedings of the Computer Vision—ECCV 2006.

[139] Park G.H., Cho H.H., Choi M.R. (2008). A contrast enhancement method using dynamic range separate histogram equalization. IEEE Trans. Consum. Electron..

[140] Peixoto C.S.B. (2003). Estudo de Métodos de Agrupamento e Transformada de Hough para Processamento de Imagens Digitais. Master’s Thesis.

[141] Pujari J., Pushpalatha S., Padmashree D. Content-Based Image Retrieval using color and shape descriptors. Proceedings of the 2010 International Conference on Signal and Image Processing.

[142] Rong F., Du-wu C., Bo H. A Novel Hough Transform Algorithm for Multi-objective Detection. Proceedings of the 2009 Third International Symposium on Intelligent Information Technology Application.

[143] Schettini R., Gasparini F., Corchs S., Marini F., Capra A., Castorina A. (2010). Contrast image correction method. J. Electron. Imaging.

[144] Setiawan A.W., Mengko T.R., Santoso O.S., Suksmono A.B. Color retinal image enhancement using CLAHE. Proceedings of the International Conference on ICT for Smart Society 2013: “Think Ecosystem Act Convergence”, ICISS 2013.

[145] Shene C.K. (2018). Geometric Transformations. https://pages.mtu.edu/~shene/COURSES/cs3621/NOTES/geometry/geo-tran.html.

[146] Shiao Y.H., Chen T.J., Chuang K.S., Lin C.H., Chuang C.C. (2007). Quality of compressed medical images. J. Digit. Imaging.

[147] Shorten C., Khoshgoftaar T.M. (2019). A survey on Image Data Augmentation for Deep Learning. J. Big Data.

[148] Singh P.K., Tiwari V. Normalized Log Twicing Function for DC Coefficients Scaling in LAB Color Space. Proceedings of the International Conference on Inventive Research in Computing Applications, ICIRCA 2018.

[149] Sun K., Wang B., Zhou Z.Q., Zheng Z.H. (2011). Real time image haze removal using bilateral filter. Trans. Beijing Inst. Technol..

[150] Unel F.O., Ozkalayci B.O., Cigla C. The Power of Tiling for Small Object Detection. Proceedings of the 2019 IEEE/CVF Conference on Computer Vision and Pattern Recognition Workshops (CVPRW).

[151] Wang K., Fang B., Qian J., Yang S., Zhou X., Zhou J. (2020). Perspective Transformation Data Augmentation for Object Detection. IEEE Access.

[152] Wang S., Zheng J., Hu H.M., Li B. (2013). Naturalness Preserved Enhancement Algorithm for Non-Uniform Illumination Images. IEEE Trans. Image Process..

[153] Warner R., Dikeman M., Devine C. (2014). Measurement of Meat Quality | Measurements of Water-holding Capacity and Color: Objective and Subjective. Encyclopedia of Meat Sciences.

[154] Yadav G., Maheshwari S., Agarwal A. Contrast limited adaptive histogram equalization based enhancement for real time video system. Proceedings of the 2014 International Conference on Advances in Computing, Communications and Informatics (ICACCI).

[155] Yang Q., Tan K.H., Ahuja N. Real-time O(1) bilateral filtering. Proceedings of the 2009 IEEE Conference on Computer Vision and Pattern Recognition.

[156] Ye H., Shang G., Wang L., Zheng M. A new method based on hough transform for quick line and circle detection. Proceedings of the 2015 8th International Conference on Biomedical Engineering and Informatics (BMEI).

[157] Ye Z., Mohamadian H., Ye Y. Discrete Entropy and Relative Entropy Study on Nonlinear Clustering of Underwater and Arial Images. Proceedings of the 2007 IEEE International Conference on Control Applications.

[158] Yuen H., Princen J., Illingworth J., Kittler J. (1990). Comparative study of Hough Transform methods for circle finding. Image Vis. Comput..

[159] Zhang H., Cisse M., Dauphin Y.N., Lopez-Paz D. MixUp: Beyond empirical risk minimization. Proceedings of the 6th International Conference on Learning Representations, ICLR 2018—Conference Track Proceedings.

[160] Zhao H., Li Q., Feng H. (2008). Multi-Focus Color Image Fusion in the HSI Space Using the Sum-Modified-Laplacian and a Coarse Edge Map. Image Vis. Comput..

[161] Silva A.D.D., Carneiro M.B.P., Cardoso C.F.S. (2018). Realce De Microaneurimas Em Imagens De Fundo De Olho Utilizando Clahe. Anais do V Congresso Brasileiro de Eletromiografia e Cinesiologia e X Simpósio de Engenharia Biomédica.

