# Supplementary material for: A New Approach for Detecting Fundus Lesions Using Image Processing and Deep Neural Network Architecture Based on YOLO Model
Source: Sensors (Basel). 2022 Aug 26;22(17):6441. doi: 10.3390/s22176441 (PMC9460625; doi:10.3390/s22176441)
Supplement: Supplementary file 1 [file sensors-22-06441-s001.zip › sensors-1809688-supplementary.pdf]

Supplementary Materials: Pre-Processing, Image Preparation, and Data Augmentation in Details

Carlos Santos [1,2], Marilton Aguiar [2], Daniel Welfer [3] and Bruno Belloni [4]

1. Pre-Processing and Image Preparation in details

1.1. The Median Filter

The median filter is used in image processing to remove noise while preserving edges [1]. The advantage of this filter is that it maintains the spatial resolution and the main details of the image while isolated points are removed [2]. This filter provides noise reduction with less blurring compared to similar linear smoothing filters [3]. In Figure S1 we present the results obtained with the application of the Median Filter with a filter of size 5×5 in a fundus image of the DDR dataset after the artificial introduction of noise *Salt & Pepper, Gaussian* and *Speackle* [1], to validate the effectiveness of the filter.

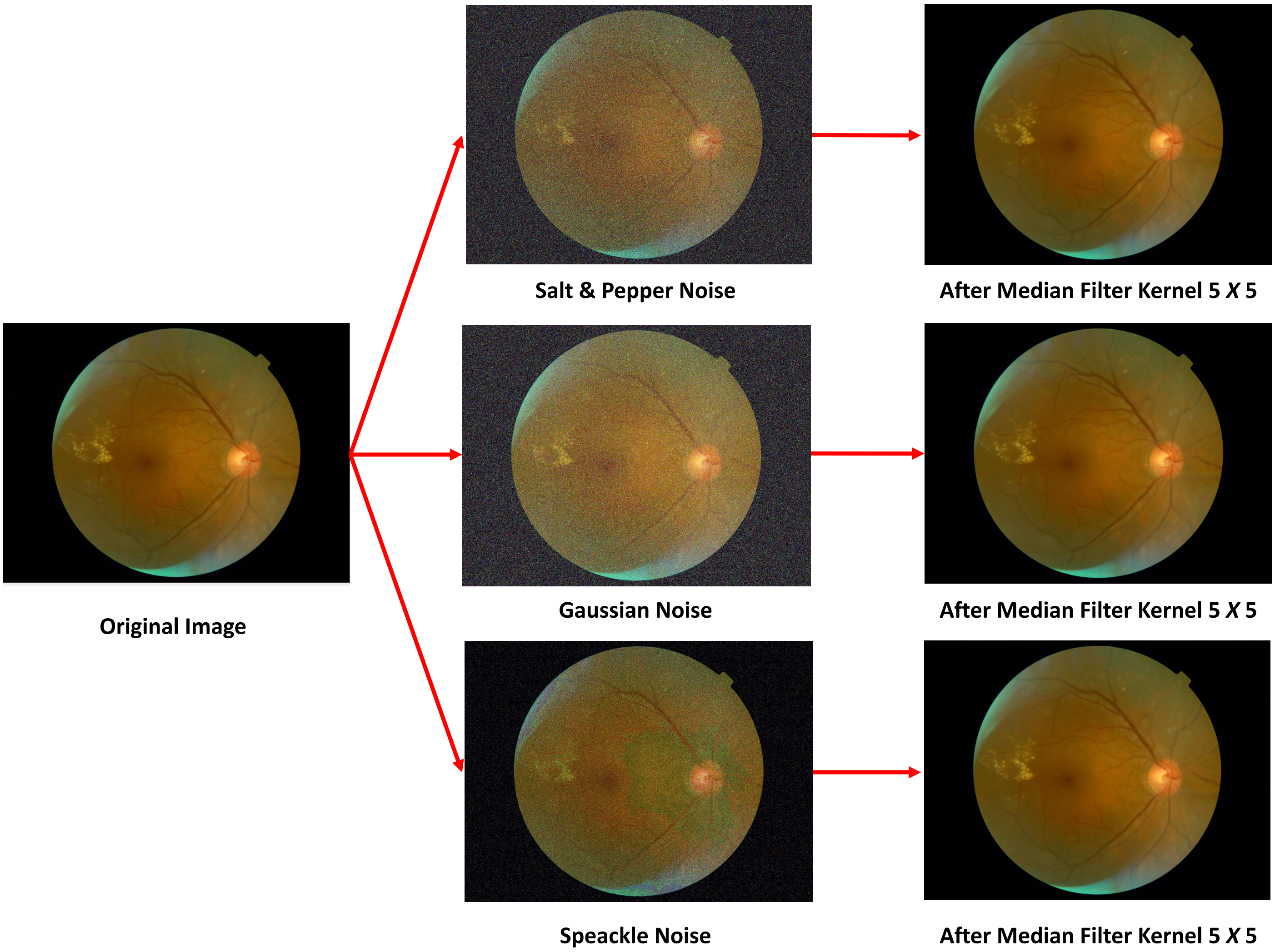

Figure S1. Removing noise like *Salt & Pepper*, *Gaussian* and *Speackle* with the application of Median Filter with *kernel* of size 5×5 in a fundus image of the DDR dataset.

We analyzed the images using the metric *PSNR* (*Peak Signal-To-Noise Ratio*) [4,5], which calculates in decibels the peak signal-to-noise ratio between two images, whose measurement measures the quality between the original image and a compressed image,

such that the higher the $PSNR$ the better the quality of the compressed or reconstructed image [6]. The *Mean Squared Error* (MSE) and the peak signal-to-noise ratio ($PSNR$) are used to compare the compression quality of the image. The MSE represents the cumulative square error between the compressed image and the original image [7], while the $PSNR$ represents a measure of the peak error [5]. The smaller the value of MSE, the smaller the error. To calculate $PSNR$, first calculate MSE using Equation 1.

$$MSE = \frac{\sum_{m,n} [I_1(m,n) - I_2(m,n)]^2}{M * N} \tag{1}$$

where M and N are the number of rows and columns in the input images. Then the $PSNR$ is calculated using Equation 2.

$$PSNR = 10log_{10}\left(\frac{R^2}{MSE}\right) \tag{2}$$

where, R is the maximum fluctuation in the input image data type. For example, if the input image has a double-precision floating-point data type, R will be 1. If it has an 8-bit unsigned integer data type, R will be 255.

Table S1 presents the results obtained with the metric $PSNR$ after removing noises like *Salt & Pepper*, *Gaussian* and *Speackle* by applying the Median Filter of size 5×5 on a fundus image of the DDR dataset. Through the objective analysis obtained with $PSNR$, we verified that the Median Filter obtained good results in reducing the three main types of noise, mainly in *Gaussian* and *Salt & Pepper*.

Table S1. Results obtained with the metric $PSNR$ after removing noises like *Salt & Pepper*, *Gaussian* and *Speackle* with the application of the Median Filter with *kernel* of size 5×5 in a fundus image of the DDR dataset.

**Noise types**	$PSNR$ **- Image with noise**	$PSNR$ **- Median Filter *kernel*** 5×5
Salt & Pepper	8.0949	12.9978
Gaussian	7.9777	12.9978
Speackle	11.4344	12.9978

1.2. Contrast-Limited Histogram Adaptive Equalization and Colos Spaces

The contrast-limited histogram adaptive equalization technique (CLAHE) [8,9] was used to enhance the images. This technique was initially developed for low contrast image enhancement and is an evolution of the histogram equalization method [10], and has been used as part of preprocessing *pipeline* to improve quality medical images [11]. The algorithm divides the image into small regions (blocks) and applies the histogram equalization in each of these regions, as illustrated in Figure S2. The advantage of this technique is to consider the local equalization of the image, with the definition of a gray level threshold.

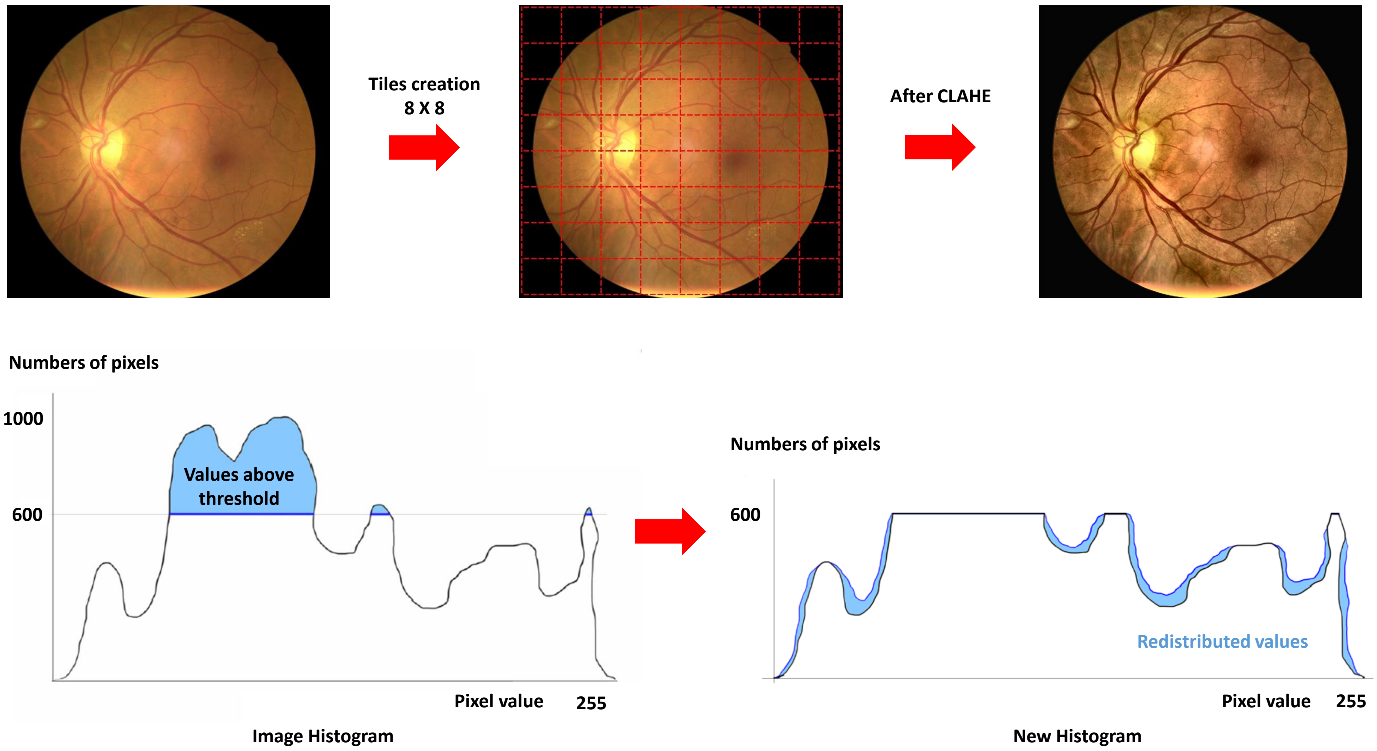

Figure S2. CLAHE divides the image into blocks (*tiles*) of size 8 × 8 and applies histogram equalization in each of these regions so that the values of *pixels* that are above a threshold preset are redistributed into a new histogram.

Differently conventional histogram equalization techniques that operate on an image as a whole, CLAHE is a method that processes small regions of the image and combines them with bilinear interpolation to avoid generating artifacts [12]. As in the works of Park *et al.* [13], Setiawan *et al.* [14], Yadav *et al.* [15], and D. D. Silva *et al.* [16], we apply the CLAHE with blocks of size 8 × 8 to perform the histogram equalization in each of these regions causing the values of *pixels* that are above a predefined threshold to be redistributed into a new histogram. The CLAHE pseudocode is described in Algorithm 1 [15].

Algorithm 1 CLAHE pseudocode

1: Process image acquisition.
2: Defines the parameters configuration ▷ Number of *tiles*; number of *bins* (histogram bins used to construct a contrast-enhancing transformation) used in the histogram; *Clip Limit* (contrast enhancement limit); distribution type (histogram presentation form), etc.
3: Split the original image into *tiles*.
4: Pre-process each *tile* obtained in the previous step.
5: Generate the gray level mapping and cut the histogram. ▷ In *tile*, the number of *pixels* are divided equally in each gray level, so the average number of *pixels* in gray level is obtained according to Equation 3.
6: Interpolate the gray level mapping to create the enhanced image.

$$N_{avg} = \frac{N_{CR-Xp} * N_{CR-Yp}}{N_{gray}} \tag{3}$$

where: N_{avg} indicates the average number of *pixels*; N_{gray} is the gray level number of *tiles*; N_{CR-Xp} is the number of *pixels* in the x direction of *tiles*; N_{CR-Yp} is the number of *pixels* in the y direction of *tiles*.

Afterwards, the calculation of the current *Clip Limit* is performed according to Equation 4.

$$N_{CL} = N_{CLIP} * N_{avg} \tag{4}$$

However, before applying the CLAHE algorithm to the fundus images of the dataset, it was necessary to define the most suitable color space to perform the image enhancement. Figure S3 presents an illustration of the different color spaces used in the experiments of the proposed work, where on the left is the RGB model (in the form of a cube); in the center, the HSV model (in the form of a cone); and on the right, the LAB model (in the form of a sphere).

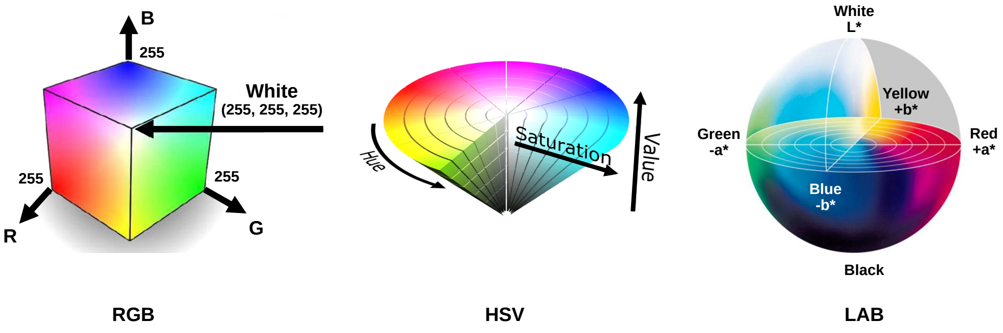

Figure S3. Illustration of the different color spaces used in the experiments of the proposed work. On the left, the RGB model (cubic format); in the center, the HSV model (conical shape); and on the right, the LAB model (spherical shape).

DDR dataset images are in the RGB color space. In an RGB model, a digital image consists of three image planes, each of which stores the values of R (*Red*), G (*Green*) and B (*Blue*) [17]. RGB is ideal for generating images, for monitors or cameras, for example, but the enhancement effects in this color space, such as contrast and entropy information, are very limited [17]. The three RGB channels have a strong correlation because they all contain brightness in their formation, so applying highlighting directly to these components often fails to achieve the desired results [18]. There is a difficulty in specifying a color through three primary colors, since the information of colors and light intensity (brightness) are together, making it difficult for processes in which these components need to be analyzed separately. To get around this problem, first, the original images were split into three independent R, G, and B images. Next, we applied the CLAHE algorithm only to the G channel, the lesions such as microaneurysms present better contrast in this channel compared to the R and B channels [16].

The HSV color space is based on the theory of human visual perception and is suitable for describing and interpreting colors [19]. The HSV model defines a color in space in terms of H (*Hue*), S (*Saturation*) and V (*Value*) components. This color space decouples achromatic information (V component, value related to brightness), from chromatic information (H and S components), in a color image [20]. Compared with the RGB color space, the HSV color space is closer to the color perception of the human eye. Another advantage is that the component related to brightness can be modified independently of the other components, being less sensitive to noise [21]. Its importance is given by the possibility of analyzing and/or modifying the brightness intensity levels present in the image independent of the H and S channels. To apply the CLAHE algorithm in this color space, first, we convert the original image to the HSV space, and then we highlight only the V channel, related to the brightness intensity of the image.

The LAB color space has a wide color variety, can express all the colors perceived by the human eye, and compensates for the distribution problem of RGB [18]. The LAB color space, also known as CIE-LAB, was defined by the *International Commission on Illumination* (CIE)

in 1976, whose composition has a dimension (L), related to luminosity, and dimensions A and B related to colors [22].

LAB is based on the opposing color model of human vision, where red and green form an opposing pair and blue and yellow form another opposing pair [22,23]. Also, this color space is very close to the human visual system, so there is more information compared to RGB, for example [24]. Its main advantage is that it allows tones and colors to be balanced interactively and independently. The LAB color space has the colors evenly distributed, with the Luminosity (L) channel being separated from the chromaticity (A and B). The use of this color space is the most accurate way to arrive at an exact color, as it is possible to reproduce all existing colors in the visible spectrum. To apply the CLAHE algorithm in this color space, we first convert the original image to LAB format, and then we only enhance the L channel, related to the image luminosity.

In the experiments, as in the work proposed by Alyoubi *et al.* [9], we used a size of *tiles* 8×8 [9] with *Clip Limit* equal to 6 in all spaces of investigated colors. We also use *bins* equal to 300 and *distribution* equal to *rayleigh*. The purpose of enhancing an image is to increase the contrast of objects that have low contrast, aiming at a better visualization of them. However, qualitatively measuring the enhancement obtained in an image is not simple [16], as this type of evaluation varies from person to person, including subjective criteria [25]. Thus, there are objective metrics that can help to estimate the contrast quantitatively [26]. However, these metrics do not necessarily determine whether the image is of good or bad quality concerning enhancement, but they often explain important characteristics of the image [27].

To quantitatively measure the best contrast obtained in the images after enhancement, we used the metrics *Measure of Enhacement* (EME) [28], and Entropy (E) [29]. Initially, we calculated the Entropy of the fundus images to assess the enhancement improvement after the application of the CLAHE algorithm. Entropy is a statistical measure of randomness that measures the average information of a random outcome. In the case of image evaluation, high values mean that all gray levels have the same probability [25]. Therefore, this measurement indicates the image's contrast level, as this information is related to the distribution of *pixels* along with the histogram. Thus, being $P(x_i)$ the probability of the gray level i, the entropy $E(x)$ is defined by Equation 5 [29].

$$E(x) = -\sum_{i=0}^{255} P(x_i) \log P(x_i) \tag{5}$$

Therefore, this metric indicates the overall contrast of the image, where high values portray the widening of the histogram and the increase in frequency uniformity of each *pixel* [25]. After verifying the Entropy of the images, we calculated the EME of the highlighted images to verify the best results obtained in the RGB, HSV, and LAB color spaces. EME was a metric originally proposed in the work of Lentz and Grigoryan [28], to calculate the quality of highlighting in digital images.

The method used by the EME metric consists of dividing the image into a matrix of sub-images w, where each sub-image is a square matrix. Ideally, this sub-matrix should have the same dimension as the *tiles* (sub-matrixes into which the CLAHE method splits the image to apply the highlighting). After dividing the highlighted image into sub-matrixes, for each sub-matrix, the ratio between the *pixels* that have higher and lower gray intensity is calculated. Then, the magnitude of the ratio is calculated, thus generating a value EME for each sub-matrix. Finally, the EME value of the image is obtained by calculating the average of the EME values obtained in each sub-matrix, thus quantifying the contrast improvement of the image after the enhancement, according to the Equation 6 [16].

$$EME = \frac{1}{K_1 K_2} \sum_{l=1}^{K_2} \sum_{k=1}^{K_1} 20 \log \left(\frac{I^W_{MAX}; k, l}{I^W_{MIN}; k, l} \right) \tag{6}$$

where: K_1 indicates the number of lines in the sub-picture matrix; K_2 the number of columns in the sub-picture array; I^W_{MAX} the maximum gray level intensity in the subpic w; and, I^W_{MIN} the minimum gray level intensity in the subpicture w.

This measure is related to local contrast so that high values indicate high local contrast, while values close to zero indicate homogeneous regions [25]. To apply the EME metric, we used the size of 8×8 as the size of sub-blocks (sub-images), the same value presented in the works of Huynh-The *et al.* [30], dos Santos [25] and D. D. Silva *et al.* [16]. After enhancing the fundus images with CLAHE in the RGB(G), HSV(V), and LAB(L) color spaces, we had to convert these images to gray levels to apply the EME metric and evaluate the contrast improvement obtained. in each color space. These grayscale images were used only for objective evaluation purposes with the EME metric.

With the CLAHE algorithm applied to enhance the fundus images in the RGB, HSV, and LAB color spaces, we verified improvements in the contrast of the lesions, both through the subjective evaluation of the images and their histograms, as well as through the objective analysis performed using the Entropy metrics. and *Measure of Enhancement*. We noticed that the significant increase in Threshold (*Clip Limit*) in the CLAHE parameterization generated artifacts in some regions of the images. The objective evaluation of the contrast obtained in 15 fundus images of the DDR set is presented in Tables S2 and S3.

Table S2. Results obtained with the Entropy metric after applying the CLAHE algorithm to the fundus images of the DDR dataset in the RGB(G), HSV(V), and LAB(L) color spaces.

**Images**	E **CLAHE + RGB(G)**	E **CLAHE + HSV(V)**	E **CLAHE + LAB(L)**
#007-1974-100	5.3406	4.8777	4.7159
#007-3319-200	4.5547	4.5681	4.1461
#007-3336-200	6.2273	5.8598	5.9846
#007-3338-200	6.2343	5.5323	5.9828
#007-3387-200	7.2854	6.7330	5.9985
#007-3396-200	5.4818	5.4452	4.9015
#007-3427-200	6.0415	5.7405	5.0914
#007-3939-200	6.2916	5.8231	5.7162
#007-4273-200	5.4263	5.7404	4.5563
#007-6335-400	5.9840	5.7604	4.7208
#007-6361-400	5.2315	4.8563	4.5155
#007-6686-400	6.8045	6.3330	6.2147
#007-6717-400	6.7205	6.3416	5.7634
#007-6722-400	6.6873	6.4085	6.1214
#007-6926-400	5.3639	5.3364	5.1495

Table S3. Results obtained with the *EME* metric after applying the CLAHE algorithm to the fundus images of the DDR dataset in the RGB(G), HSV(V), and LAB(L) color spaces.

Images	*EME* CLAHE + RGB(G)	*EME* CLAHE + HSV(V)	*EME* CLAHE + LAB(L)
#007-1974-100	3.5948	3.2152	2.1602
#007-3319-200	2.1958	2.0634	1.1713
#007-3336-200	3.0931	2.9379	1.9840
#007-3338-200	3.5287	2.6052	2.2821
#007-3387-200	3.7507	3.6807	2.0064
#007-3396-200	3.9257	3.5753	2.5867
#007-3427-200	3.9591	4.2571	3.2447
#007-3939-200	2.9237	2.7346	1.6858
#007-4273-200	2.1647	1.9182	1.1121
#007-6335-400	3.7807	4.5371	2.5995
#007-6361-400	3.1811	3.0705	1.7046
#007-6686-400	6.1646	6.0830	4.3246
#007-6717-400	7.0138	6.9563	4.2578
#007-6722-400	6.7473	6.5823	4.0060
#007-6926-400	4.2441	3.9220	3.2276

Based on these results, in the analysis of the histograms and the images generated after the application of CLAHE, it is possible to affirm that the best enhancement results were obtained in the RGB(G) and LAB(L) color spaces, respectively, and the images in the LAB color space produced the best results, maintaining a good level of highlighting, as can be seen in Figure S4.

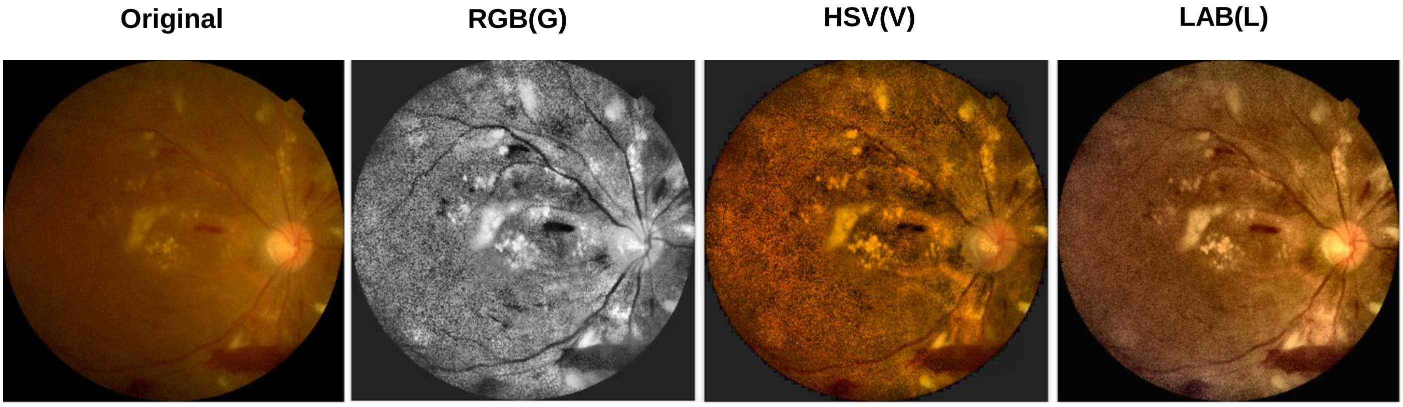

Figure S4. Example of contrasts obtained with the CLAHE algorithm on channels (G) of RGB, (V) of HSV, and (L) of LAB, using *tile* size of 8 × 8 and Clip Limit equal to 6 .

Thus, we opted for the LAB color space to perform the enhancement because the color images present characteristics of fundus lesions that could be lost if we opted for the images in the (G) RGB channel. The application of pre-processing with the CLAHE algorithm-generated better quality images, adjusting the contrast and reducing noise. However, even with the most adequate threshold adjustment for the fundus images, the generation of some artifacts was observed after the application of CLAHE. In this sense, the next pre-processing step was the application of a filter to smooth the images, with the purpose however of creating after minimizing the artifacts that were without, losing, important characteristics of the images, such as the of the images. lesions we intend to detect.

After enhancement the fundus images with CLAHE, we convert the images to the RGB standard and apply the Bilateral Filter to provide the smoothing of the images. The Bilateral Filter [31–33] is a non-linear smoothing filter that preserves edge detail. The filter

takes into account the distance between *pixels* in space (called spatial proximity similarity) and also considers the degree of similarity between *pixels* (called grayscale similarity) [18]. This filter is similar to the Gaussian Filter in that it finds the Gaussian weighted average in the neighborhood, but takes into account the difference in *pixel* by blurring the nearby *pixels*. The Bilateral Filter is represented by Equation 7 [18].

$$BF(I) = \left\{ \sum_{n \in \Omega} w(x,n)g(n) \right\} * \left\{ \sum_{n \in \Omega} w(x,n) \right\}^{-1} \tag{7}$$

where: $BF(I)$ is an image using two-sided filtering; $w(x,n)$ is a two-sided filtering weight function; $g(n)$ is the *haze image*; and, Ω is the local area with x as the center. The weight function can be expressed as:

$$w(x,n) = w_{\sigma_d} * w_{\sigma_r} \tag{8}$$

where: w_{σ_d} is the kernel function of space; w_{σ_r} is the kernel value of the domain function; σ_d is the variation of the influence of spatial proximity similarity; and, σ_r is the variance of the similarity factors influencing in gray scale. w_{σ_d} and w_{σ_r} can be expressed as:

$$w_{\sigma_d} = \exp\left[-\frac{d_d^2(x,(x-n))}{2\sigma_d^2} \right] = \exp\left[-\frac{n^2}{2\sigma_d^2} \right] \tag{9}$$

$$w_{\sigma_r} = \exp\left[-\frac{d_d^2(x,(x-n))}{2\sigma_r^2} \right] = \exp\left[-\frac{(g(x)-g(x-n))^2}{2\sigma_r^2} \right] \tag{10}$$

Although the Bilateral Filter has a higher processing time than other smoothing filters, it has the advantage of ensuring that only *pixels* with an intensity similar to the central *pixel* are blurred, while the other *pixels* with different values are not blurred. By doing this, the edges that have the greatest variation in intensity are preserved. Therefore, the Bilateral Filter preserves the edges, as the *pixels* at the edges will vary in intensity.

We used a filter size $d = 9$ (diameter of each neighborhood of *pixel* during filtering) to perform a deeper noise filtering and $\sigma = 75$ so that the lesions present in the images fundus are not blurred. For measurement purposes, we checked the $PSNR$ of a CLAHE-enhanced fundus image where we got the result of 18.9808. After smoothing this same image with the Bilateral Filter, we obtained a $PSNR$ of 19.0772. We verified the $PSNR$ of the other images and found that after smoothing with the Bilateral Filter we obtained an improvement in the peak signal-to-noise ratio of the images, as well as a reduction in *outliers* from the enhancement process.

In Figure S5 we present fundus images from the DDR dataset, before and after the pre-processing and preparation stage, where on the left is the original image and its gray level histogram, and on the right is the preprocessed image with its histogram in gray level.

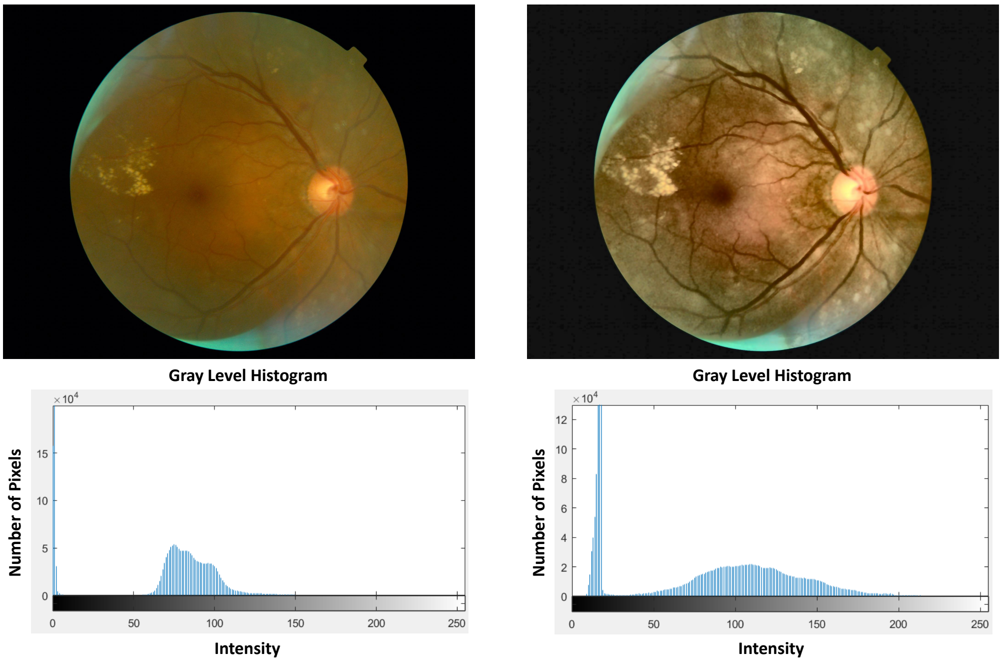

Figure S5. Fundus image of the DDR dataset before and after the Pre-Processing and Preparation step. On the left the original image and its gray level histogram and on the right the pre-processed image with its gray level histogram.

1.3. Useless Background Suppression

Even as the works by El abbadi and Hammod [34] and Alyoubi *et al.* [9], we performed a pre-processing step for partial *cropping* of the black background of the retinal images, as illustrated in Figure S7. According to El abbadi and Hammod [34], the importance of removing the black background from the retinal image is related to the generation of false positives during the detection of lesions, especially at the border of the retina, where there is a similarity of the retinal border with the blood vessels. In the case of fundus images, only the *pixels* of the retina have significant information, the rest is considered the background, therefore, it is important to locate the area of interest and remove unwanted features related to the background of the image.

First, we perform retinal detection in fundus images using the Hough Transform (HT), a technique that locates shapes in images, being used to extract lines, circles, and ellipses (or conic sections) [35]. HT is a widely used method for curve detection and recognition due to its robustness and processing power [36], being typically used to detect or segment geometric objects from images [37]. HT is a popular method in Computer Vision [38], for detecting shapes that are easily parameterized (lines, circles, ellipses, etc) in computer images. In general, this transform is applied after the image goes through pre-processing, commonly edge detection [37].

The Hough Transform applies a transformation to the image such that all points belonging to the same curve are mapped to a single point in parameter space, also called Hough space [39]. Each edge of an image is transformed by mapping to determine cells in the parameter space, indicated by the primitives defined through the analyzed point. These cells are incremented and the local maximum of the accumulator at the end of the process will indicate the parameters corresponding to the specified form [37]. The HT is applied to the data of an edge map obtained in the image segmentation step and allows detecting practically any type of curve, even those that are not very visible and very noisy [40].

Although HT was introduced for the detection of lines, it is possible to generalize it to detect circles, ellipses or even any parameterizable curve in the form $h(v, c)$, where v is the vector of coordinates and c is the parameter array. The disadvantage of using the HT is the computational effort resulting from the increase in the dimensionality of the parameter

space, as these curves can no longer be represented in a two-dimensional space [39]. Let λ be a circle with center (a, b) and radius r of the plane $x - y$ represented by Equation 11:

$$\lambda : (x - a)^2 + (y - b)^2 = r^2 \quad (11)$$

Through the HT a point (x_i, y_i) belonging to λ can be transformed into a conic surface λ' of the three-dimensional parametric plane a, b, r , defined by Equation 12:

$$\lambda : (a - x_i)^2 + (b - y_i)^2 = r^2 \quad (12)$$

Even as the parametric space, the accumulators will also be three-dimensional for the circular configurations in the image. Thus, the matrix of accumulators will be constructed, where a value N in a cell (a_i, b_j, r_k) will indicate that N points belong to the circle $lambda'$ in the plan x, y defined by $(x - a_i)^2 + (y - b_j)^2 = (r_k)^2$. The extraction of existing circles in the image is carried out through the heavy weight accumulators, which represent the points with the highest number of intersections between the parametric cones [39].

Before identifying the retinal contour, we performed the pre-processing, in which we applied the Median Filter to smooth the images and eliminate irrelevant details for the detection of the retinal circumference. Afterward, we performed the detection of the contours through the HT. When the shape of the retina is found in the fundus image, then the mapping of all of its points in the parameter space group around the parameter values correspond to its shape. It should be noted that before applying HT, it was necessary to convert the images to grayscale and detect the edges using the Sobel Filter [41,42].

We use the *Hough Gradient 2-1 Hough Transform* (21HT) [43] detection method, which runs Hough in two stages [44]. In the first stage, a transform is accumulated to find the center coordinates. In the second stage, a radius histogram is constructed for each candidate center obtained in the first stage [43]. The choice of 21HT was due to the method having good performance and requiring little space for storage, having the disadvantage of low performance in the detection of relatively small circular shapes [45], which did not impact the detection of the retina due to your size.

Using the 21HT method, it was possible to find the retina, which corresponded to the largest circumference present in the fundus images. After locating the retina, we transform its circumference into its equivalent rectangle using the coordinates x_{min} and y_{min} – referring to the upper left position in the image – and the coordinates x_{max} and y_{max} – referring to the lower right position in the image, as shown in Figure S6. The area outside the red rectangle corresponds to the crop area performed on the fundus image.

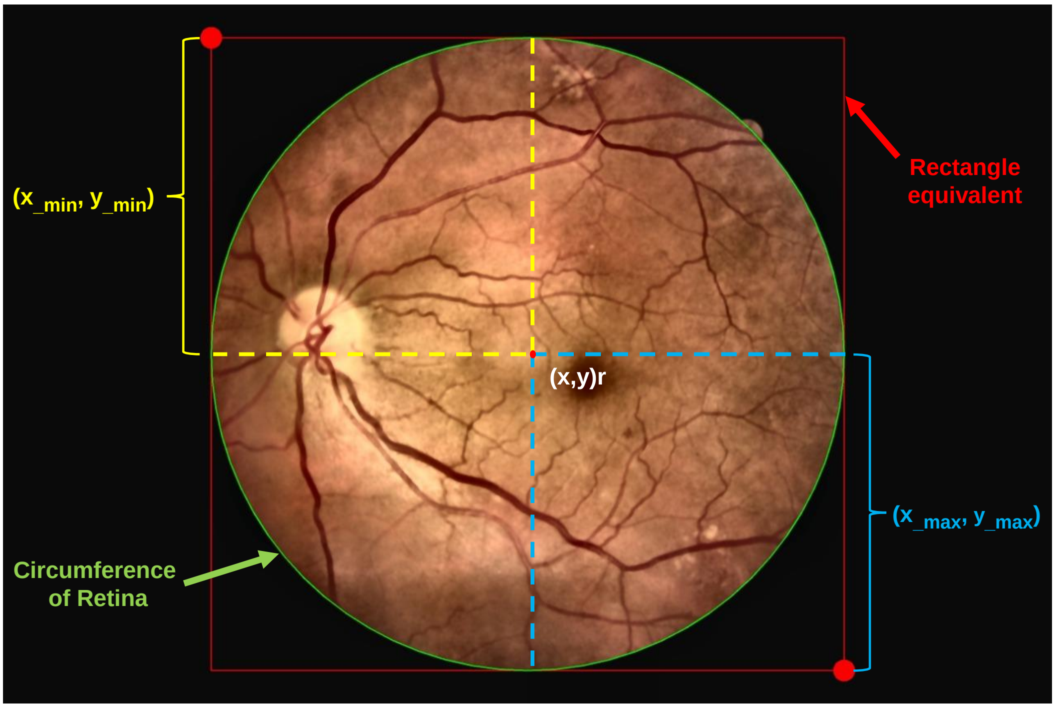

Figure S6. Transforming the retinal circumference into its equivalent rectangle using the coordinates x_{min} and y_{min} (top left position), and the coordinates x_{max} and y_{max} (bottom right position).

In Figure S7, we present an illustration of the results obtained during the *Cropping* step applied to the fundus images, in which it is possible to verify the resulting image after identifying the retinal contour and partial cropping of the black background of the image.

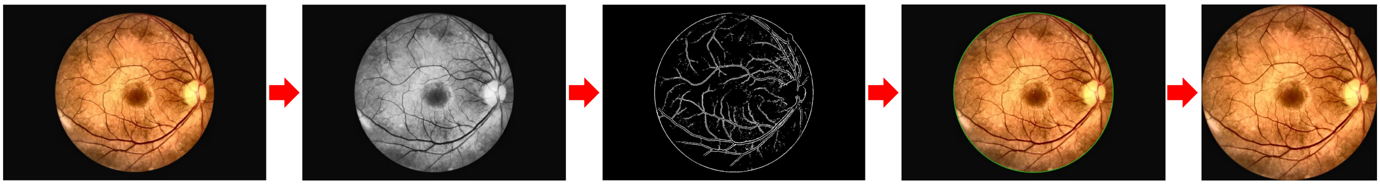

Figure S7. *Pipeline* to perform the *Cropping* process. First the images are pre-processed for smoothing with the Median Filter with *kernel* of size 5×5. Then the images are converted to grayscale for edge detection with Sobel Filter. Afterwards, the retinal contour is demarcated (in green) by means of the Hough Transform (21HT). Finally, the black background of the images is partially cropped.

1.4. Tilling the original images

The last step of the pre-processing block of the proposed approach (as illustrated in Figure 1 in the original manuscript) is the realization of *Tilling*. Deep neural networks that perform single-stage object detection are suitable for use in edge devices due to their low computational cost and inference speed, especially when compared to architectures based on region proposals. Even so, the detection of very small objects still remains a challenge for deep neural networks that perform single-stage detection [46].

According to Unel *et al.* [46], Fei-Fei Li [47], object detection methods based on deep neural networks can be categorized into single-stage and two-stage. Two-stage methods are based on region proposals, which involves generating region proposals followed by classification, such as models R-CNN [48], Fast R-CNN [49], Faster R-CNN [50], SPP-net [51], R-FCN [52], FPN [53] and Mask R-CNN [54]. Single-stage methods are based on regression/classification in a unified framework for detection and classification, such

as SSD [55] and YOLO [56] models. Although methods of the first category (based on proposed regions) provide more accurate results, they have high computational costs and high inference time. On the other hand, methods of the second category (based on regression/classification in a single network structure) are faster and less computationally expensive compared to two-stage models but have difficulty detecting very small objects.

In general, models that perform object detection are trained and evaluated on datasets with a large number of examples, such as ImageNet [57] and COCO [58]. Datasets typically involve low resolution images (256 × 256, for example), including these relatively large objects with large *pixel* coverage. Thus, models trained under these assumptions are often successful in detecting objects with this type of input image. However, these assumptions are not always reflected in the detection of objects in medical images, especially when the objects are very small and the images are not generated by high definition cameras [46].

The small coverage of *pixels* due to the low resolution of the images and/or a limited sampling of images affects the ability to train and generalize models based on deep learning. However, deep neural networks also have problems dealing with high-resolution images due to the computational cost required to process these images. Then, these images must be resized to be passed on to the input layer of the neural network, responsible for reading these images. When performing this resizing process, the images are reduced in resolution, impairing the extraction of features from retinal microlesions. Bochkovskiy *et al.* [59] state that when optimizing a deep neural network the objective is to find the balance between the resolution of the input image of the network, the number of convolutional layers, and the number of network parameters and the number of output layers (filters).

In fundus images, the detection of very small lesions (microlesions) is a challenge, as in the case of microaneurysms. If the lesion area is not large enough, the signal propagated in the convolutional layers will be small while the model training is performed, leading to gradient dissipation. In addition, very small objects are more susceptible to data labeling errors, where lesion identification can be impaired. In the DDR dataset the fundus lesions are made available with their bounding box annotations (*Ground Truth*) in *eXtensible Markup Language* (XML) format. For purposes of exemplification, in the DDR dataset approximately 10,978 lesion annotations, out of a total of 38,012, are smaller than 3 *pixels*.

The approach we initially adopted was to train the deep neural network models by reducing the images to sizes like 416 × 416, 512 × 512, and 640 × 640 *pixels*, taking into account the maximum limits defined in the model architectures used in the experiments. Furthermore, resolutions above the aforementioned values made it impossible to carry out the due to the high computational experiments and the time for training the models. However, the strategy of reducing the size of the images resulted in problems in approaching and arising from the loss of lesion details and the consequent reduction of the precision of the proposed approach

After reducing the resolution of the images, some microlesions practically disappeared, given the small size of these lesions. During the experiments, we also noticed that when using higher resolution images, such as 640 × 640 *pixels*, there was an increase in the precision of lesion detection. Therefore, the increase in the resolution of the images used in the neural network input layer provides a larger receptive field around the lesion and a greater number of features are accessible for training of the network..

The solution we adopted to avoid the need to reduce the resolution of the original images of the DDR dataset, without using GPUs with greater computational power and without making significant changes in the architecture of the neural network, was the implementation of the method *Tilling* (slicing), where the original images are cut into blocks (*tiles*). Then, the blocks resulting from this operation are used in the input layer of the deep neural network to train the model.

The images were split into smaller sub-images, where the size of the resulting sub-images varied according to the original size of each image in the DDR dataset. An example of *Tilling* created from a fundus image is illustrated in Figure S8. It is important to note that in the proposed approach, *tiles* of size 2 × 2 were created. Each sub-image generated

in this process remained with its respective lesions and annotations (*Ground Truth*), with no loss of information. After the application of *Tilling*, a greater amount of information was preserved after the application of Tilling around the lesions of the sub-images created compared to the images originals that had their resolution reduced (Pixels Per Inch) to be used in the input layer of the neural network, causing a greater loss of information around the lesions.

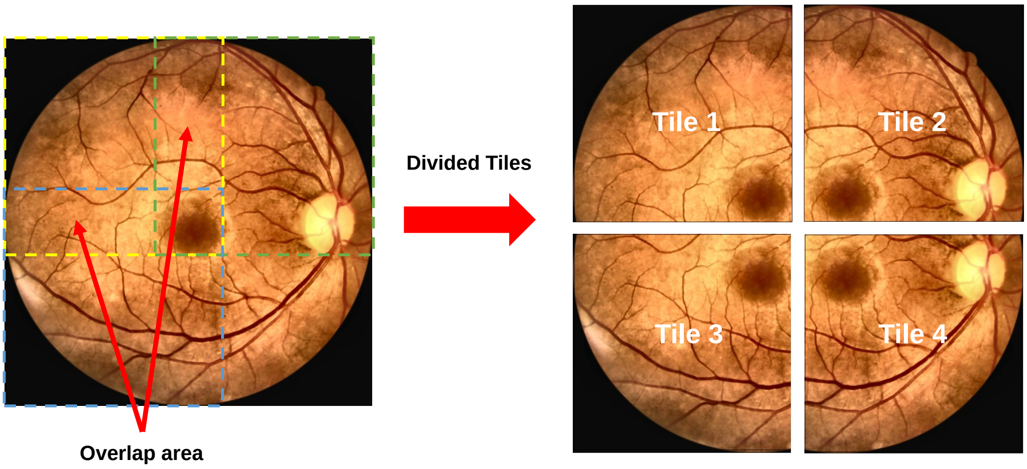

Figure S8. On the left a fundus image of the DDR dataset and on the right an example of sub-images (*tiles*) created from the original image with *Tilling* of size 2 × 2, and 15% *overlap* area between blocks.

When image partitioning is performed to create *tiles*, lesions may be present at the clipping location of these blocks. To minimize the risk of information loss from these lesions, we define an *overlap* area, in which each block will have an overlap area of 15% with its neighboring blocks, as illustrated in Figure S8. Thus, the information of the lesions that are present in the place of clipping of the blocks is not lost, as this information will be replicated in different blocks. To solve the problem of information redundancy of the replicated lesions, we used the technique of *Non-Max Suppression* (NMS) [60], in which only the bounding box with the highest *IoU* is considered, from so that the other bounding boxes predicted for the same object are discarded [61].

After using the *Tilling* method, we verified an increase in the precision of lesion detection, as shown in Tables 4–7 (in the original manuscript), in which we demonstrate the results obtained by the proposed approach in the Validation and Testing steps using *Tilling* of size 2 × 2 and without *Tilling*. However, in the experiments we verified that the increase in the amount of *tiles* did not provide a significant increase in the precision of the proposed approach and, for this reason, we chose to use blocks of size 2 × 2.

We performed *Tilling* to reduce the loss of lesion details due to the reduction of image resolution for training the neural network architecture. Thus, we divided the images into blocks to be used in the input layer of the neural network to increase the receptive field of the microlesions present in the fundus images, since the lesions present in the original high-resolution images are treated as objects larger after block creation.

To improve the performance of the proposed approach, especially in the detection of microlesions, we explored pre-processing techniques for better smoothing and enhancement of the fundus images. In addition, we partially removed the black background that caused the generation of false positives, and *Tilling* the original images to use the resulting image blocks in the training of the deep neural network.

2. Data Augmentation

A frequently encountered impediment to training deep neural networks is the unavailability of public datasets with large amounts of labeled objects [62]. This problem is related to the cost of acquiring the images and the need for specialists to annotate the objects, espe-

cially when dealing with medical images. By artificially increasing the number of examples in a dataset, the possibility of underfitting the model is reduced, in addition to improving its generalization ability, since the model is trained to predict objects in situations that were not initially present. in the original dataset. Classic approaches to data augmentation in object classification problems include applying basic geometric transformations and color space transformations [63,64].

2.1. The Mosaic

The first data augmentation method applied was *Mosaic*, initially proposed in the work of Bochkovskiy *et al.* [59], in which we combine four random fundus sub-images to create a new image. It should be noted that the four sub-images that form the resulting image have different proportions. With this data augmentation method, it was possible to extract more features of the lesions in the same fundus image. Another advantage was the reduction in the need for very large batch sizes since more images were trained in the same batch. Furthermore, the new images created with *Mosaic* allowed the neural network to learn characteristics of the lesions that were not present in the original images, given the randomness and asymmetry of the sub-images that make up the new images, consequently increasing the ability to generalize the proposed approach. Next, Figure S9 presents an example of data augmentation from the *Mosaic* method applied to the original images. This made it possible to improve the precision of the proposed approach, even training the neural network with smaller batch sizes.

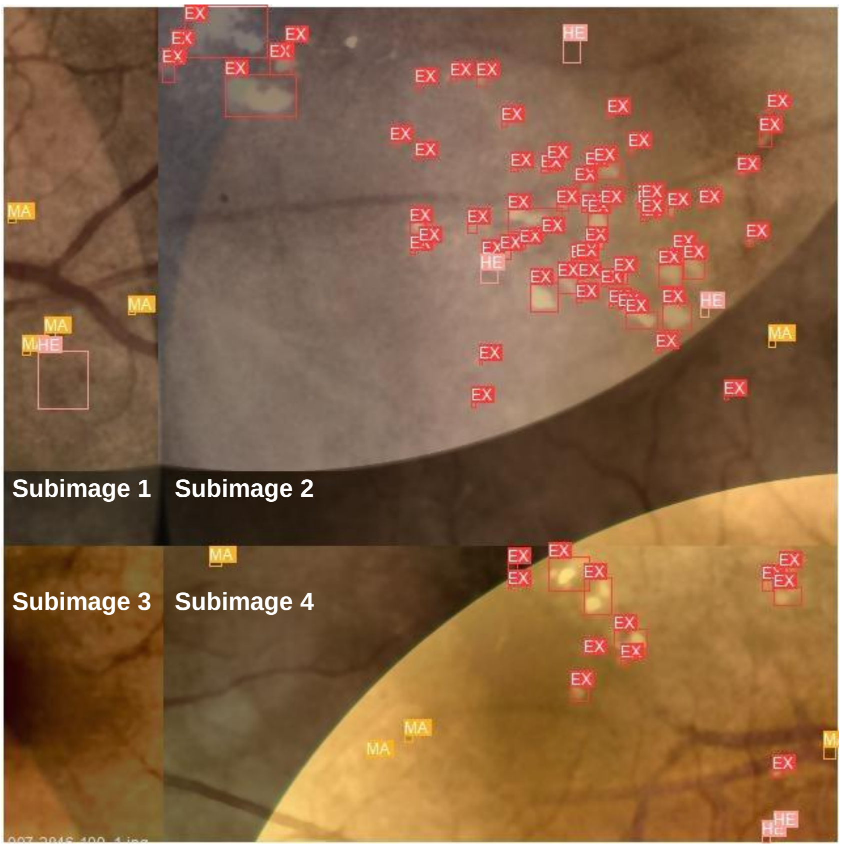

Figure S9. Example of data augmentation obtained with the *Mosaic* method, in which four random sub-images, with different proportions, are combined to form a new image.

2.2. The Copy-Paste

Another technique applied to increase data was *Copy-Paste*, based on the work of Dwibedi *et al.* [62], Dvornik *et al.* [63] and Ghiasi *et al.* [65], in which artificial images with lesions and labels were copied from other fundus images in the same dataset. With this

method, it is possible to create images with larger amounts of annotated lesions, on different *backgrounds*. The objective is to enable the neural network to extract more characteristics from replicated lesions, under different contexts, increasing the ability to predict these lesions. Using the *Copy-Paste* method it is also possible to mitigate the problem associated with the reduced amount of labeled lesions available in the public Diabetic Retinopathy datasets. In addition, as we apply *Copy-Paste* using the segmentation mask of each lesion available in the dataset as a basis, both the lesion and its label are fully copied to the new image, ensuring the integrity of the copied information.

According to Dwibedi *et al.* [62], in a cross-domain setup, where synthetic data is combined with only 10% of real data, the *Copy-Paste* outperforms the results method obtained by models trained on only real data. According to the authors, the performance improvement was over 21% on *benchmark* datasets. Ghiasi *et al.* [65] also report an improvement in segmentation and detection of instances of the COCO dataset, having achieved 49.1 of AP in the detection of masks and 57.3 of AP in the detection of bounding boxes, an improvement of $+0.6$ of AP in masks and $+1.5$ of AP in bounding boxes over the prior art.

The *Copy-Paste* method was initially designed to work with the instance segmentation task [62] and not for object detection since its purpose is to copy an object in its entirety and paste it into another image. How YOLO works with annotations in the form of bounding boxes ($[class\ x, y, width, height]$) and non-polygons ($[class\ x_1, y_1, x_2, y_2, x_3, y_3, \ldots, x_n, y_n]$), commonly used for instance segmentation, it is not appropriate to copy all the content (background) contained in a bounding box together with the lesion and paste it into a new image, because during training a large number of False Positives, impairing the performance regarding the detection of lesions. To workaround this problem, we generate the lesion annotations in the form of polygons from the segmentation masks of these lesions that are available together with the images of the DDR dataset.

To perform the process of creating fundus lesion annotations in the form of polygons, first, the lesion annotations (*Ground Truth*) are imported from the DDR dataset. Then, the information from the images and annotations are obtained so that the image files with the binary masks of the lesions that are available in the dataset are then loaded. Thus, with the help of the OpenCV function `find_contours()`, the binary masks of the lesions are used to capture the contour of the lesions. After identifying the contour of the lesions, annotations are created using the `create_annotation_format()` function. Finally, the previously created annotations are used to generate annotations in the standard COCO JSON format through the `get_coco_json_format()` function.

With the annotations of the fundus lesions in the form of polygons, it was possible to copy only the lesions (excluding the background around the lesion) and paste them into the images generated using the *Copy-Paste* technique. However, it is important to note that when training the neural network, the copied lesions must have an annotation in the bounding box format so that it is possible to perform the training of the deep neural network. For this purpose, the `segment2box()` function of YOLOv5 was used, which converts the label in the form of a segment $(xy1, xy2, \ldots)$ to a label in the form of a box $(xyxy)$.

Figure S10 presents an example of a fundus image of the DDR dataset with the annotations of the fundus lesions, where (a) are examples of the annotations generated in the format of bounding boxes that are used to train the proposed approach and (b) are examples of the annotations generated in the polygon format that is used to apply the *Copy-Paste* method.

With the *Copy-Paste* method we augment the dataset by generating additional training data by copying lesions from an image to new fundus images. We randomly copy lesions and their bounding box annotations and paste them on random backgrounds, as proposed in the work by Dwibedi *et al.* [62]. In Figure S11, we present an example of data augmentation performed with *Copy-Paste* on the fundus images of the DDR dataset, in which the flagged lesions were randomly copied along with its bounding box to a new image.

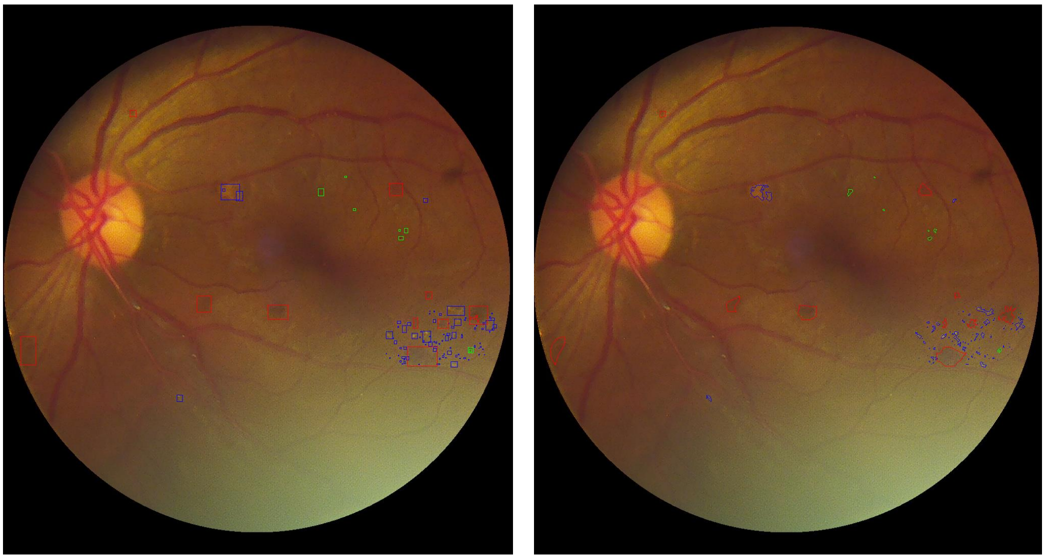

(a) (b)

Figure S10. Example of fundus image of the DDR dataset with annotations of fundus lesions: (a) annotations in the format of bounding boxes that are used for training the neural network; and, (b) polygon format annotations that are used to apply the *Copy-Paste* method. Fundus lesions can be identified by colors: Hard Exudates (EX), in blue; Microaneurysms (MA), in green; and, Hemorrhages (HE), in red.

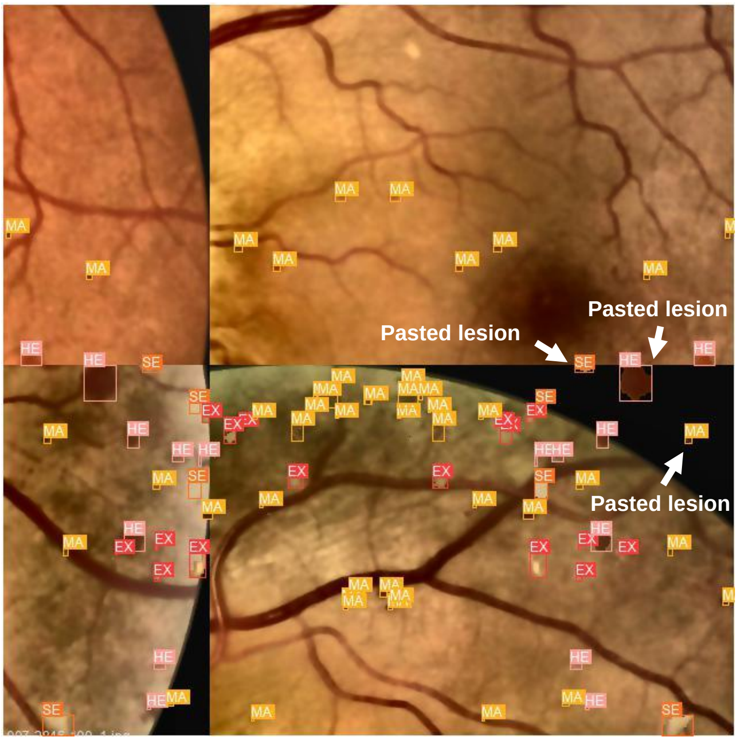

Figure S11. Example of data augmentation performed with the *Copy-Paste* method applied to fundus images. Three retinal lesions are flagged with their bounding boxes that were randomly copied from other fundus images in the DDR dataset and randomly pasted into a new image.

2.3. The MixUp

The next data augmentation method we used was *MixUp* [66–68], responsible for randomly creating new examples by combining images. The main motivation of this

method is to minimize the overfitting of deep neural networks during the training stage [66]. Despite its simplicity, *MixUp* has been shown to substantially improve the generalizability of models across a wide range of Computer Vision tasks [66,69].

Very dense deep neural networks can present memory problems and high sensitivity to adversarial examples. Improving generalizability and sensitivity to perturbations in the input data remains a challenge [70]. The main motivation of this method is to minimize overfitting in training deep neural networks [66]. Despite its simplicity, the *MixUp* method has been shown to substantially improve the ability to generalize models across a wide range of Computer Vision tasks [66,69].

Figure S12 shows an example of data augmentation resulting from the use of *MixUp* in the fundus images of the DDR dataset. The purpose is to combine characteristics of different images, as well as the annotations of bounding boxes of the lesions present in the images used in the combination. Therefore, the objective of *MixUp* is to make the neural network not very confident about the relationship between lesion characteristics and annotations, making the proposed approach more sensitive to learning from adversarial examples and, consequently, improving its generalization ability. We configured the proposed approach to applying the *MixUp* method randomly to 50% of the images because during the experiments values above this percentage caused a decrease in the predictive capacity of the model.

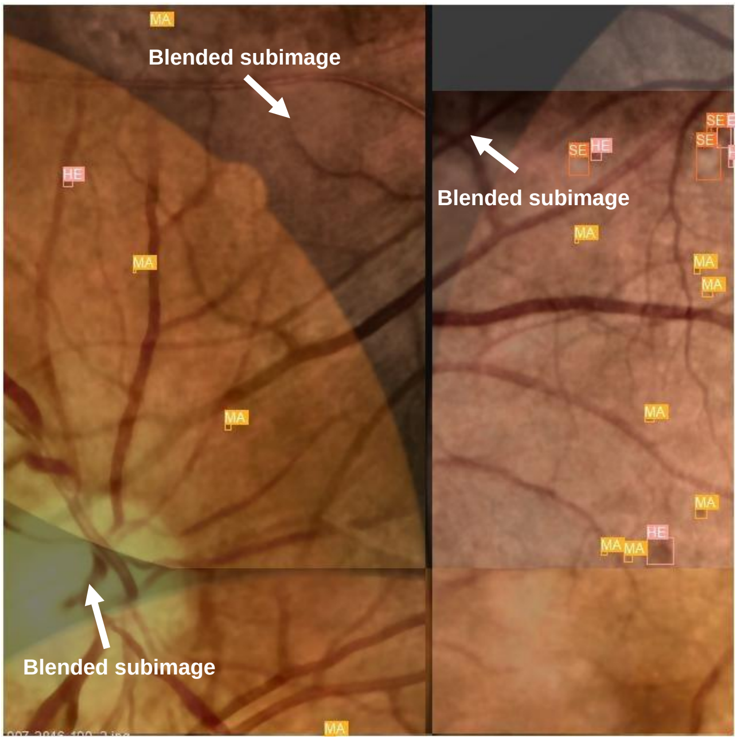

Figure S12. Example of image resulting from the application of the methods *Mosaic+MixUp* on fundus images of the DDR dataset, in which the different sub-images combined can be observed according to the flags.

2.4. The Geometric Transformations

We also performed data augmentation through geometric transformations that were applied to the original images. Geometric transformations are easily implemented and provide good solutions for positional biases present in training data [64]. Some of its drawbacks, however, include the computational cost in the transformation and the additional training time [64]. In addition, some geometric transformations, such as translation or random shear, must be applied carefully so that the original labels of an object present in the image are not altered.

We applied six geometric transformations to the fundus images of the DDR dataset: *vertical flip up-down*, *horizontal flip left-right*, *scale*, *perspective*, *translation* and *shear*. It is possible to verify in Figure S13 the transformations on the lesions and their bounding boxes. While creating the artificial images, we preserved the annotations (bounding boxes) of the lesions so that the original annotations of these lesions were not lost after we performed the geometric transformations.

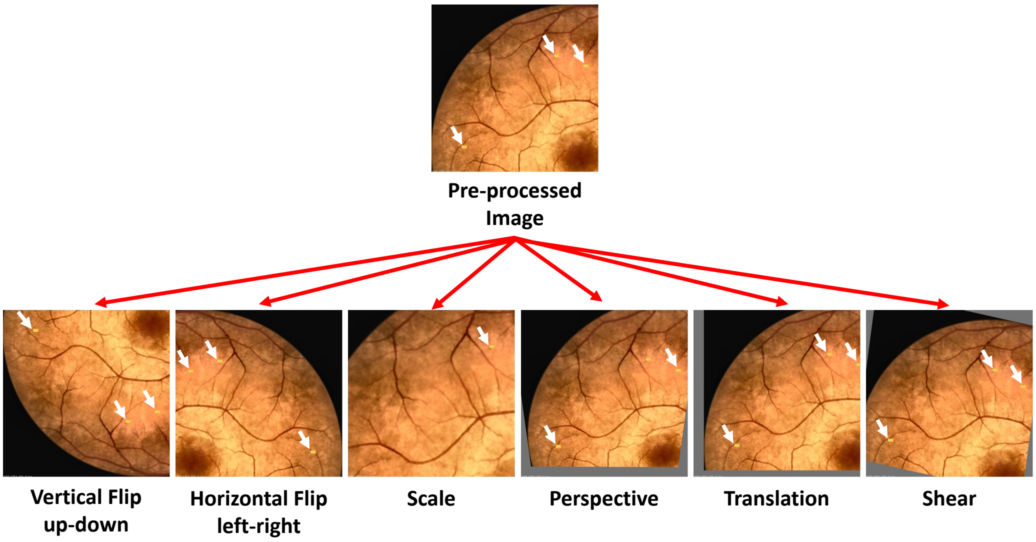

Figure S13. Example of increasing data on images through geometric transformations: *Vertical flip up-down*, *Horizontal flip left-right*, *Scale*, *Perspective*, *Translation*, and *Shear*.

In the *flipping* geometric transformation the image is flipped horizontally or vertically. Inversion rearranges *pixels* while protecting image features. It is one of the simplest techniques to implement, being used in datasets such as CIFAR-10 and ImageNet [64]. Second Hao *et al.* [71], *vertical flip* flips the input image along its x axis (top to bottom), while *horizontal flip* flips the input image along along its y axis (from left to right).

In the *scaling* geometric transformation the image is scaled outwards (*zoom out*), or inwards (*zoom in*). Scale transformations increase or decrease a given object and, as a result, change lengths and angles. The meaning of the scale transformation is to increase the xp coordinate times. This requirement satisfies $x' = xp$ and therefore $x = x'/p$ [72].

When the human eye views a scene, objects at a distance appear smaller than objects closer. This phenomenon is known as perspective. The camera works on the same principle as human vision [73]. The use of the *perspective* geometric transformation is inspired by the phenomenon of camera perspective. In this transformation, parallelism, length, and angle are not preserved, but collinearity and incidence. This means that straight lines will remain straight even after the transformation. It can also be applied to projectively distort an image to another image plane. For example, instead of looking at a scene directly ahead, we can see it from another point of view through the *perspective* transformation.

In the geometric transformation *translation* the image is shifted to various areas along the x axis or y axis. Shifting images left, right, up, or down can be a very useful transformation to avoid positional bias in the data [64]. A translation slides an object a fixed distance in a certain direction. The original object and its translation have the same shape and size, and they are facing the same direction, which means just moving an image without rotating or resizing it, for example.

The *shearing* geometric transformation gives the impression of "pushing" a geometric object in a direction parallel to a coordinate plane (3d) or a coordinate axis (2d), moving one side of the image and transforming it from a square shape to a trapezoid shape [72]. This transformation is different from rotation in that an axis is fixed and the image is stretched

to a certain angle called the shear angle [74]. Table S4 presents the parameters used to apply the geometric transformations on the fundus images.

Table S4. Parameters used to apply geometric transformations to fundus images

Method	Description	Parameter
Vertical flip	Flips the input vertically around the axis x	0.5
Horizontal flip	Flip the input vertically around axis y	0.5
Scale	Zooms in or zooms out the transformed image	$[-0.5 : 1.5]$
Perspective	Applies random four-point perspective input according to a predefined scale	$[0 : 0.0005]$
Translation	Applies translation in the horizontal and vertical direction according to a range	$[0.9 : 1.1]$
Shear	Applies 15º shear on the horizontal or vertical axis according to a range	$[0.05 : 1.05]$

References

1. Jasim, M.K.; Najm, R.; Kanan, E.H.; Alfaar, H.E.; Otair, M. Image Noise Removal Techniques: A Comparative Analysis, 2019.
2. Faria, D. Trabalhos Práticos Análise e Processamento de Imagem, 2010.
3. Gonzalez, R.; Woods, R. *Processamento Digital de Imagens*, 3ª ed.; Pearson Prentice Hall: São Paulo, 2010.
4. Fardo, F.A.; Conforto, V.H.; de Oliveira, F.C.; Rodrigues, P.S. A Formal Evaluation of PSNR as Quality Measurement Parameter for Image Segmentation Algorithms, 2016, [arXiv:cs.CV/1605.07116].
5. Erfurt, J.; Helmrich, C.R.; Bosse, S.; Schwarz, H.; Marpe, D.; Wiegand, T. A Study of the Perceptually Weighted Peak Signal-To-Noise Ratio (WPSNR) for Image Compression. In Proceedings of the 2019 IEEE International Conference on Image Processing (ICIP), 2019, pp. 2339–2343. https://doi.org/10.1109/ICIP.2019.8803307.
6. Shiao, Y.H.; Chen, T.J.; Chuang, K.S.; Lin, C.H.; Chuang, C.C. Quality of compressed medical images. *Journal of Digital Imaging* **2007**, *20*, 149–159. https://doi.org/10.1007/s10278-007-9013-z.
7. Asamoah, D.; Ofori, E.; Opoku, S.; Danso, J. Measuring the Performance of Image Contrast Enhancement Technique. *International Journal of Computer Applications* **2018**, *181*, 6–13. https://doi.org/10.5120/ijca2018917899.
8. Santos, C.; De Aguiar, M.S.; Welfer, D.; Belloni, B. Deep Neural Network Model based on One-Stage Detector for Identifying Fundus Lesions. In Proceedings of the 2021 International Joint Conference on Neural Networks (IJCNN), 2021, pp. 1–8. https://doi.org/10.1109/IJCNN52387.2021.9534354.
9. Alyoubi, W.L.; Abulkhair, M.F.; Shalash, W.M. Diabetic Retinopathy Fundus Image Classification and Lesions Localization System Using Deep Learning. *Sensors* **2021**, *21*. https://doi.org/10.3390/s21113704.
10. Rai, R.; Gour, P.; Singh, B. Underwater Image Segmentation using CLAHE Enhancement and Thresholding. *International Journal of Emerging Technology and Advanced Engineering* **2012**, *2*, 118–123.
11. Horry, M.J.; Chakraborty, S.; Paul, M.; Ulhaq, A.; Pradhan, B.; Saha, M.; Shukla, N. COVID-19 Detection Through Transfer Learning Using Multimodal Imaging Data. *IEEE Access* **2020**, *8*, 149808–149824. https://doi.org/10.1109/ACCESS.2020.3016780.
12. Mukhopadhyay, S.; Mandal, S.; Pratiher, S.; Changdar, S.; Burman, R.; Ghosh, N.; Panigrahi, P.K. A comparative study between proposed Hyper Kurtosis based Modified Duo-Histogram Equalization (HKMDHE) and Contrast Limited Adaptive Histogram Equalization (CLAHE) for Contrast Enhancement Purpose of Low Contrast Human Brain CT scan images. *CoRR* **2015**, *abs/1505.06219*, [1505.06219].
13. Park, G.H.; Cho, H.H.; Choi, M.R. A contrast enhancement method using dynamic range separate histogram equalization. *IEEE Transactions on Consumer Electronics* **2008**, *54*, 1981–1987. https://doi.org/10.1109/TCE.2008.4711262.
14. Setiawan, A.W.; Mengko, T.R.; Santoso, O.S.; Suksmono, A.B. Color retinal image enhancement using CLAHE. *Proceedings - International Conference on ICT for Smart Society 2013: "Think Ecosystem Act Convergence", ICISS 2013* **2013**, pp. 215–217. https://doi.org/10.1109/ICTSS.2013.6588092.
15. Yadav, G.; Maheshwari, S.; Agarwal, A. Contrast limited adaptive histogram equalization based enhancement for real time video system. In Proceedings of the 2014 International Conference on Advances in Computing, Communications and Informatics (ICACCI), 2014, pp. 2392–2397. https://doi.org/10.1109/ICACCI.2014.6968381.
16. D. D. Silva, A.; B. P. Carneiro, M.; F. S. Cardoso, C. Realce De Microaneurimas Em Imagens De Fundo De Olho Utilizando Clahe. In Proceedings of the Anais do V Congresso Brasileiro de Eletromiografia e Cinesiologia e X Simpósio de Engenharia Biomédica, 2018, pp. 772–775. https://doi.org/10.29327/cobecseb.78909.
17. Ma, J.; Fan, X.; Yang, S.X.; Zhang, X.; Zhu, X. Contrast Limited Adaptive Histogram Equalization-Based Fusion in YIQ and HSI Color Spaces for Underwater Image Enhancement. *International Journal of Pattern Recognition and Artificial Intelligence* **2018**, *32*, 1–26. https://doi.org/10.1142/S0218001418540186.
18. Dai, F.; Fan, B.; Peng, Y. An image haze removal algorithm based on blockwise processing using LAB color space and bilateral filtering. *2018 Chinese Control And Decision Conference (CCDC)* **2018**, pp. 5945–5948.

19. Liu, Z.; Chen, W.; Zou, Y.; Hu, C. Regions of interest extraction based on HSV color space. In Proceedings of the IEEE 10th International Conference on Industrial Informatics, 2012, pp. 481–485. https://doi.org/10.1109/INDIN.2012.6301214.
20. Gonzalez, R.C.; Woods, R.E.; Eddins, S.L. *Digital Image Processing Using MATLAB*; Prentice-Hall, Inc.: USA, 2003.
21. Zhao, H.; Li, Q.; Feng, H. Multi-Focus Color Image Fusion in the HSI Space Using the Sum-Modified-Laplacian and a Coarse Edge Map. *Image Vision Comput.* **2008**, *26*, 1285–1295. https://doi.org/10.1016/j.imavis.2008.03.007.
22. Warner, R. Measurement of Meat Quality | Measurements of Water-holding Capacity and Color: Objective and Subjective. In *Encyclopedia of Meat Sciences (Second Edition)*, 2 ed.; Dikeman, M.; Devine, C., Eds.; Academic Press: Oxford, 2014; pp. 164–171. https://doi.org/https://doi.org/10.1016/B978-0-12-384731-7.00210-5.
23. Pujari, J.; Pushpalatha, S.; Padmashree, D. Content-Based Image Retrieval using color and shape descriptors. In Proceedings of the 2010 International Conference on Signal and Image Processing, 2010, pp. 239–242. https://doi.org/10.1109/ICSIP.2010.5697476.
24. Singh, P.K.; Tiwari, V. Normalized Log Twicing Function for DC Coefficients Scaling in LAB Color Space. *Proceedings of the International Conference on Inventive Research in Computing Applications, ICIRCA 2018* **2018**, pp. 333–338. https://doi.org/10.1109/ICIRCA.2018.8597293.
25. dos Santos, J.R.V. Avaliação de técnicas de realce de imagens digitais utilizando métricas subjetivas e objetivas. Dissertação de mestrado (engenharia de teleinformática), Universidade Federal do Ceará, Fortaleza, 2016.
26. Schettini, R.; Gasparini, F.; Corchs, S.; Marini, F.; Capra, A.; Castorina, A. Contrast image correction method. *J. Electronic Imaging* **2010**, *19*, 023005. https://doi.org/10.1117/1.3386681.
27. Wang, S.; Zheng, J.; Hu, H.M.; Li, B. Naturalness Preserved Enhancement Algorithm for Non-Uniform Illumination Images. *IEEE Transactions on Image Processing* **2013**, *22*, 3538–3548. https://doi.org/10.1109/TIP.2013.2261309.
28. Lentz, K.; Grigoryan, A. A New Measure of Image Enhancement. *IASTED International Conference on Signal Processing & Communication* **2000**, pp. 19–22.
29. Ye, Z.; Mohamadian, H.; Ye, Y. Discrete Entropy and Relative Entropy Study on Nonlinear Clustering of Underwater and Arial Images. In Proceedings of the 2007 IEEE International Conference on Control Applications, 2007, pp. 313–318. https://doi.org/10.1109/CCA.2007.4389249.
30. Huynh-The, T.; Le, B.V.; Lee, S.; Le-Tien, T.; Yoon, Y. Using weighted dynamic range for histogram equalization to improve the image contrast, 2014. https://doi.org/10.1186/1687-5281-2014-44.
31. Yang, Q.; Tan, K.H.; Ahuja, N. Real-time O(1) bilateral filtering. In Proceedings of the 2009 IEEE Conference on Computer Vision and Pattern Recognition, 2009, pp. 557–564. https://doi.org/10.1109/CVPR.2009.5206542.
32. Paris, S.; Durand, F. A Fast Approximation of the Bilateral Filter Using a Signal Processing Approach. In Proceedings of the Computer Vision – ECCV 2006; Leonardis, A.; Bischof, H.; Pinz, A., Eds.; Springer Berlin Heidelberg: Berlin, Heidelberg, 2006; pp. 568–580.
33. Sun, K.; Wang, B.; Zhou, Z.Q.; Zheng, Z.H. Real time image haze removal using bilateral filter. In Proceedings of the Transactions of Beijing Institute of Technology, 2011, Vol. 31, pp. 810–814.
34. El abbadi, N.; Hammod, E. Automatic Early Diagnosis of Diabetic Retinopathy Using Retina Fundus Images Enas Hamood Al-Saadi-Automatic Early Diagnosis of Diabetic Retinopathy Using Retina Fundus Images. *EUROPEAN ACADEMIC RESEARCH* **2014**, *2*.
35. Nixon, M.S.; Aguado, A.S. 5 - High-level feature extraction: fixed shape matching. In *Feature Extraction and Image Processing for Computer Vision (Fourth Edition)*, 4 ed.; Nixon, M.S.; Aguado, A.S., Eds.; Academic Press, 2020; pp. 223–290. https://doi.org/https://doi.org/10.1016/B978-0-12-814976-8.00005-1.
36. Rong, F.; Du-wu, C.; Bo, H. A Novel Hough Transform Algorithm for Multi-objective Detection. In Proceedings of the 2009 Third International Symposium on Intelligent Information Technology Application, 2009, Vol. 3, pp. 705–708. https://doi.org/10.1109/IITA.2009.387.
37. Ye, H.; Shang, G.; Wang, L.; Zheng, M. A new method based on hough transform for quick line and circle detection. In Proceedings of the 2015 8th International Conference on Biomedical Engineering and Informatics (BMEI), 2015, pp. 52–56. https://doi.org/10.1109/BMEI.2015.7401472.
38. Chandrasekar, L.; Durga, G. Implementation of Hough Transform for image processing applications. In Proceedings of the 2014 International Conference on Communication and Signal Processing, 2014, pp. 843–847. https://doi.org/10.1109/ICCSP.2014.6949962.
39. Peixoto, C.S.B. Estudo de Métodos de Agrupamento e Transformada de Hough para Processamento de Imagens Digitais. Dissertação de mestrado (curso de mestrado em matemática), Universidade Federal da Bahia, Bahia, 2003.
40. Castro, D.J.L. Garra servo-controlada com integração de informação táctil e de proximidade. Dissertação de mestrado (curso de mestrado em engenharia eletrotécnica), Universidade de Coimbra, Coimbra, 1996.
41. McREYNOLDS, T.; BLYTHE, D. CHAPTER 12 - Image Processing Techniques. In *Advanced Graphics Programming Using OpenGL*; McREYNOLDS, T.; BLYTHE, D., Eds.; The Morgan Kaufmann Series in Computer Graphics, Morgan Kaufmann: San Francisco, 2005; pp. 211–245. https://doi.org/https://doi.org/10.1016/B978-155860659-3.50014-7.
42. Hawas, A.R.; Ashour, A.S.; Guo, Y. 8 - Neutrosophic set in medical image clustering. In *Neutrosophic Set in Medical Image Analysis*; Guo, Y.; Ashour, A.S., Eds.; Academic Press, 2019; pp. 167–187. https://doi.org/https://doi.org/10.1016/B978-0-12-818148-5.00008-4.

43. Yuen, H.; Princen, J.; Illingworth, J.; Kittler, J. Comparative study of Hough Transform methods for circle finding. *Image and Vision Computing* **1990**, *8*, 71–77. https://doi.org/https://doi.org/10.1016/0262-8856(90)90059-E.
44. Illingworth, J.; Kittler, J. The Adaptive Hough Transform. *IEEE Transactions on Pattern Analysis and Machine Intelligence* **1987**, *PAMI-9*, 690–698. https://doi.org/10.1109/TPAMI.1987.4767964.
45. Marroni, L.S. Aplicação da transformada de Hough para localização dos olhos em faces humanas. Dissertação de mestrado (curso de mestrado em engenharia elétrica), Universidade de São Paulo, São Carlos, 2002.
46. Unel, F.O.; Ozkalayci, B.O.; Cigla, C. The Power of Tiling for Small Object Detection. In Proceedings of the 2019 IEEE/CVF Conference on Computer Vision and Pattern Recognition Workshops (CVPRW), 2019, pp. 582–591. https://doi.org/10.1109/CVPRW.2019.00084.
47. Fei-Fei Li, R.K..D.X. cs231n, Lecture 15 - Slide 4, Detection and Segmentation. http://cs231n.stanford.edu/slides/2021/lecture_15.pdf, 2021. [Online; accessed 26-December-2021].
48. Girshick, R.; Donahue, J.; Darrell, T.; Malik, J. Rich feature hierarchies for accurate object detection and semantic segmentation. *Proceedings of the IEEE Computer Society Conference on Computer Vision and Pattern Recognition* **2014**, pp. 580–587, [1311.2524]. https://doi.org/10.1109/CVPR.2014.81.
49. Girshick, R. Fast R-CNN. *Proceedings of the IEEE International Conference on Computer Vision* **2015**, *2015 International Conference on Computer Vision, ICCV 2015*, 1440–1448, [1504.08083]. https://doi.org/10.1109/ICCV.2015.169.
50. Ren, S.; He, K.; Girshick, R.; Sun, J. Faster R-CNN: Towards Real-Time Object Detection with Region Proposal Networks. *IEEE Transactions on Pattern Analysis and Machine Intelligence* **2017**, *39*, 1137–1149, [1506.01497]. https://doi.org/10.1109/TPAMI.2016.2577031.
51. He, K.; Zhang, X.; Ren, S.; Sun, J. Spatial Pyramid Pooling in Deep Convolutional Networks for Visual Recognition. *Lecture Notes in Computer Science* **2014**, p. 346–361. https://doi.org/10.1007/978-3-319-10578-9_23.
52. Dai, J.; Li, Y.; He, K.; Sun, J. R-FCN: Object Detection via Region-based Fully Convolutional Networks, 2016, [arXiv:cs.CV/1605.06409].
53. Li, X.; Lai, T.; Wang, S.; Chen, Q.; Yang, C.; Chen, R. Feature Pyramid Networks for Object Detection. *Proceedings - 2019 IEEE Intl Conf on Parallel and Distributed Processing with Applications, Big Data and Cloud Computing, Sustainable Computing and Communications, Social Computing and Networking, ISPA/BDCloud/SustainCom/SocialCom 2019* **2019**, pp. 1500–1504, [arXiv:1612.03144v2]. https://doi.org/10.1109/ISPA-BDCloud-SustainCom-SocialCom48970.2019.00217.
54. He, K.; Gkioxari, G.; Dollár, P.; Girshick, R. Mask R-CNN. *IEEE Transactions on Pattern Analysis and Machine Intelligence* **2020**, *42*, 386–397, [1703.06870]. https://doi.org/10.1109/TPAMI.2018.2844175.
55. Konishi, Y.; Hanzawa, Y.; Kawade, M.; Hashimoto, M. SSD: Single Shot MultiBox Detector. *Eccv* **2016**, *1*, 398–413. https://doi.org/10.1007/978-3-319-46448-0.
56. Redmon, J.; Divvala, S.; Girshick, R.; Farhadi, A. You only look once: Unified, real-time object detection. In Proceedings of the Proceedings of the IEEE Computer Society Conference on Computer Vision and Pattern Recognition, 2016, Vol. 2016-Decem, pp. 779–788. https://doi.org/10.1109/CVPR.2016.91.
57. Fei-Fei, L.; Deng, J.; Li, K. ImageNet: Constructing a large-scale image database. *Journal of Vision* **2010**, *9*, 1037–1037. https://doi.org/10.1167/9.8.1037.
58. Lin, T.Y.; Maire, M.; Belongie, S.; Hays, J.; Perona, P.; Ramanan, D.; Dollár, P.; Zitnick, C.L. Microsoft COCO: Common objects in context. *Lecture Notes in Computer Science (including subseries Lecture Notes in Artificial Intelligence and Lecture Notes in Bioinformatics)* **2014**, *8693 LNCS*, 740–755, [1405.0312]. https://doi.org/10.1007/978-3-319-10602-1_48.
59. Bochkovskiy, A.; Wang, C.Y.; Liao, H.Y.M. YOLOv4: Optimal Speed and Accuracy of Object Detection. *ArXiv e-prints* **2020**, [2004.10934]. arXiv:2004.10934.
60. Bodla, N.; Singh, B.; Chellappa, R.; Davis, L.S. Soft-NMS - Improving Object Detection with One Line of Code. *Proceedings of the IEEE International Conference on Computer Vision* **2017**, *2017-October*, 5562–5570, [1704.04503]. https://doi.org/10.1109/ICCV.2017.593.
61. Mamdouh, N.; Khattab, A. YOLO-Based Deep Learning Framework for Olive Fruit Fly Detection and Counting. *IEEE Access* **2021**, *9*, 84252–84262. https://doi.org/10.1109/access.2021.3088075.
62. Dwibedi, D.; Misra, I.; Hebert, M. Cut, Paste and Learn: Surprisingly Easy Synthesis for Instance Detection, 2017, [arXiv:cs.CV/1708.01642].
63. Dvornik, N.; Mairal, J.; Schmid, C. Modeling Visual Context is Key to Augmenting Object Detection Datasets, 2018, [arXiv:cs.CV/1807.07428].
64. Shorten, C.; Khoshgoftaar, T.M. A survey on Image Data Augmentation for Deep Learning. *Journal of Big Data* **2019**, *6*. https://doi.org/10.1186/s40537-019-0197-0.
65. Ghiasi, G.; Cui, Y.; Srinivas, A.; Qian, R.; Lin, T.Y.; Cubuk, E.D.; Le, Q.V.; Zoph, B. Simple Copy-Paste is a Strong Data Augmentation Method for Instance Segmentation, 2021, [arXiv:cs.CV/2012.07177].
66. Zhang, H.; Cisse, M.; Dauphin, Y.N.; Lopez-Paz, D. MixUp: Beyond empirical risk minimization. *6th International Conference on Learning Representations, ICLR 2018 - Conference Track Proceedings* **2018**, pp. 1–13, [1710.09412].
67. Guo, H.; Mao, Y.; Zhang, R. Augmenting Data with Mixup for Sentence Classification: An Empirical Study, 2019, [arXiv:cs.CL/1905.08941].
68. Carratino, L.; Cissé, M.; Jenatton, R.; Vert, J.P. On Mixup Regularization, 2020, [arXiv:cs.LG/2006.06049].
69. Guo, H.; Mao, Y.; Zhang, R. MixUp as Locally Linear Out-Of-Manifold Regularization, 2018, [arXiv:cs.LG/1809.02499].

70. Kim, J.H.; Choo, W.; Jeong, H.; Song, H.O. Co-Mixup: Saliency Guided Joint Mixup with Supermodular Diversity, 2021, [arXiv:cs.LG/2102.03065].
71. Hao, R.; Namdar, K.; Liu, L.; Haider, M.A.; Khalvati, F. A Comprehensive Study of Data Augmentation Strategies for Prostate Cancer Detection in Diffusion-weighted MRI using Convolutional Neural Networks, 2020, [arXiv:q-bio.QM/2006.01693].
72. Shene, C.K. Geometric Transformations. https://pages.mtu.edu/~shene/COURSES/cs3621/NOTES/geometry/geo-tran.html, 2018. Online; accessed 01-Nov-2021.
73. Wang, K.; Fang, B.; Qian, J.; Yang, S.; Zhou, X.; Zhou, J. Perspective Transformation Data Augmentation for Object Detection. *IEEE Access* **2020**, *8*, 4935–4943. https://doi.org/10.1109/ACCESS.2019.2962572.
74. Claro, M.; Vogado, L.; Santos, J.; Veras, R. Utilização de Técnicas de Data Augmentation em Imagens: Teoria e Prática. https://sol.sbc.org.br/livros/index.php/sbc/catalog/view/48/224/445-1, 2020. Online; accessed 01-Nov-2021.
